# Stereocontrolled and Adaptable Synthesis of (−)-Aspergilone A via the Key Intermediate (+)-Phenol A

**DOI:** 10.1021/acs.joc.5c01650

**Published:** 2025-09-11

**Authors:** Manuel K. Langer, Annette Bayer

**Affiliations:** Department of Chemistry, 8016 UiT The Arctic University of Norway, Tromsø NO-9037, Norway

## Abstract

We report the first total synthesis of (−)-aspergilone A, a citrinin-type azaphilone, and the determination of the absolute configuration of naturally occurring (+)-aspergilone A. The synthesis is centered around phenol A, a substructure of citrinin-type azaphilones, as the key intermediate. (+)-Phenol A was constructed using two sequential 1,2-boronate rearrangements with exceptional stereocontrol. Formally, this approach allows for the construction of all stereoisomers of phenol A without the need to change the synthetic procedure. The synthesis was completed in 11 steps with a total yield of 15.7% and >99% ee. The absolute configuration of (+)-aspergilone A was determined to be 3*R*, 4*S*.

## Graphical abstract

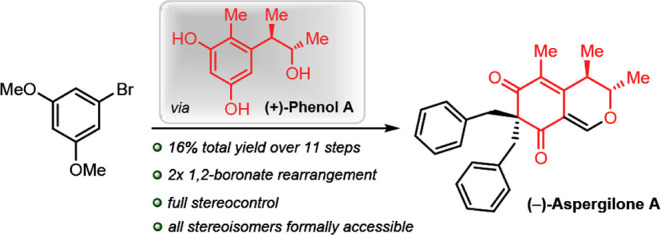

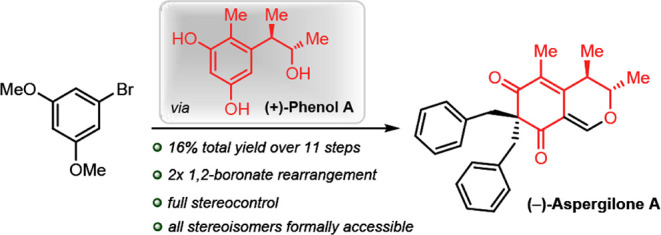

## 1 Introduction

Azaphilones are a class of fungal polyketides, featuring an isochroman core and exerting a wide range of biological activities including antimicrobial, cytotoxic, antifungal, antiviral, antioxidant, anticancer, antifouling, and antiinflammatory.^1–4^ Citrinin-type azaphilones are a structural subclass consisting of around 60 members^1,4^ and a selection of them is shown in Figure 1. The subclasses eponym, (−)-citrinin (**1**), was first isolated in 1931 ^5^ and it was not before 1963 that the absolute configuration was determined (Figure 1).^6,7^ Albeit recognized for its antimicrobial activity, its general cytotoxicity limits its use for medical applications.^8,9^

**Figure 1:**
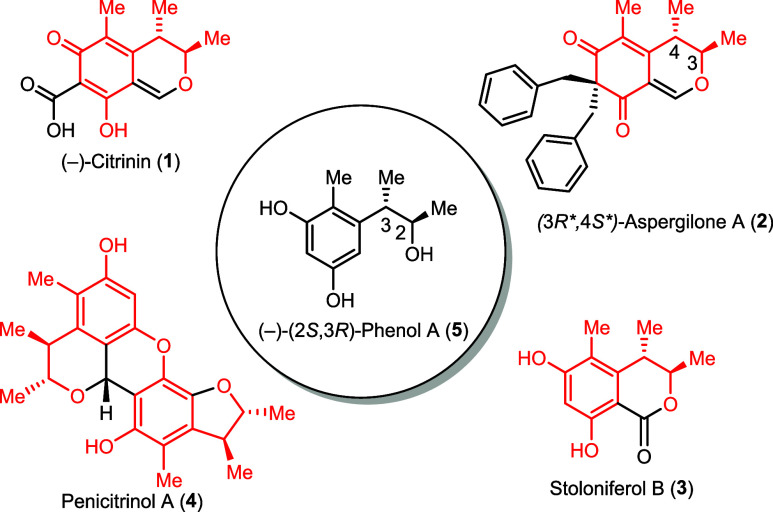
Chemical structures of (−)-citrinin (**1**), (3R*,4S*)-aspergilone A (**2**), stoloniferol B (**3**), penicitrinol A (**4**), (−)-(2*S*,3*R*) phenol A (**5**). The phenol A (**5**) substructure is highlighted in red in the natural products.

(−)-Phenol A (**5**) can be obtained by chemical degradation of (−)-**1**,^5^ but is no direct intermediate in the biosynthesis of (−)-citrinin.^10,11^ The former is a substructure found in many members of the citrinin subclass such as aspergilone A (**2**), stoloniferol B (**3**) and penicitrinol A (**4**). Phenol A (**5**) and its stereoisomers have been used as the key intermediates in a number of total syntheses of (+)- and (−)-citrinin (**1**).^12–15^ To introduce the stereogenic centers, Regan and Staunton^13^ combined an *ortho*-toluate carbanion, generated with a chiral amide base **7**, and ethanal as an electrophile (Scheme 1A). They obtained a mixture of the lactones (3*R*,4*R*)-**8** and (3*S*,4*R*)-**8**, the former of which one could be transformed into the desired product (3*S*,4*R*)-**8** via a three-step process. Unfortunately, they later discovered that they had made the unnatural (+)-citrinin (**1**). When Rödel and Gerlach^14^ employed an aryl Grignard and (2*S*)-*trans*-(−)-2,3-dimethyloxirane **10**, they obtained alcohol (2*S*,3*S*)-**11**, which needed to be converted to the desired configuration (2*R*,3*S*)-**11** by means of a Mitsunobu reaction (Scheme 1B). Ohashi and Hosokawa^16^ synthesized stoloniferol B (**3**), employing a stereoselective Mukayama aldol reaction to introduce the *trans*-1,2-dimethyl structure and then used several manipulations to first build up the lactone and then the aromatic system. None of the above approaches, while delivering the enantiopure natural products, allows facile synthesis of all the respective stereoisomers.

**Scheme 1:**
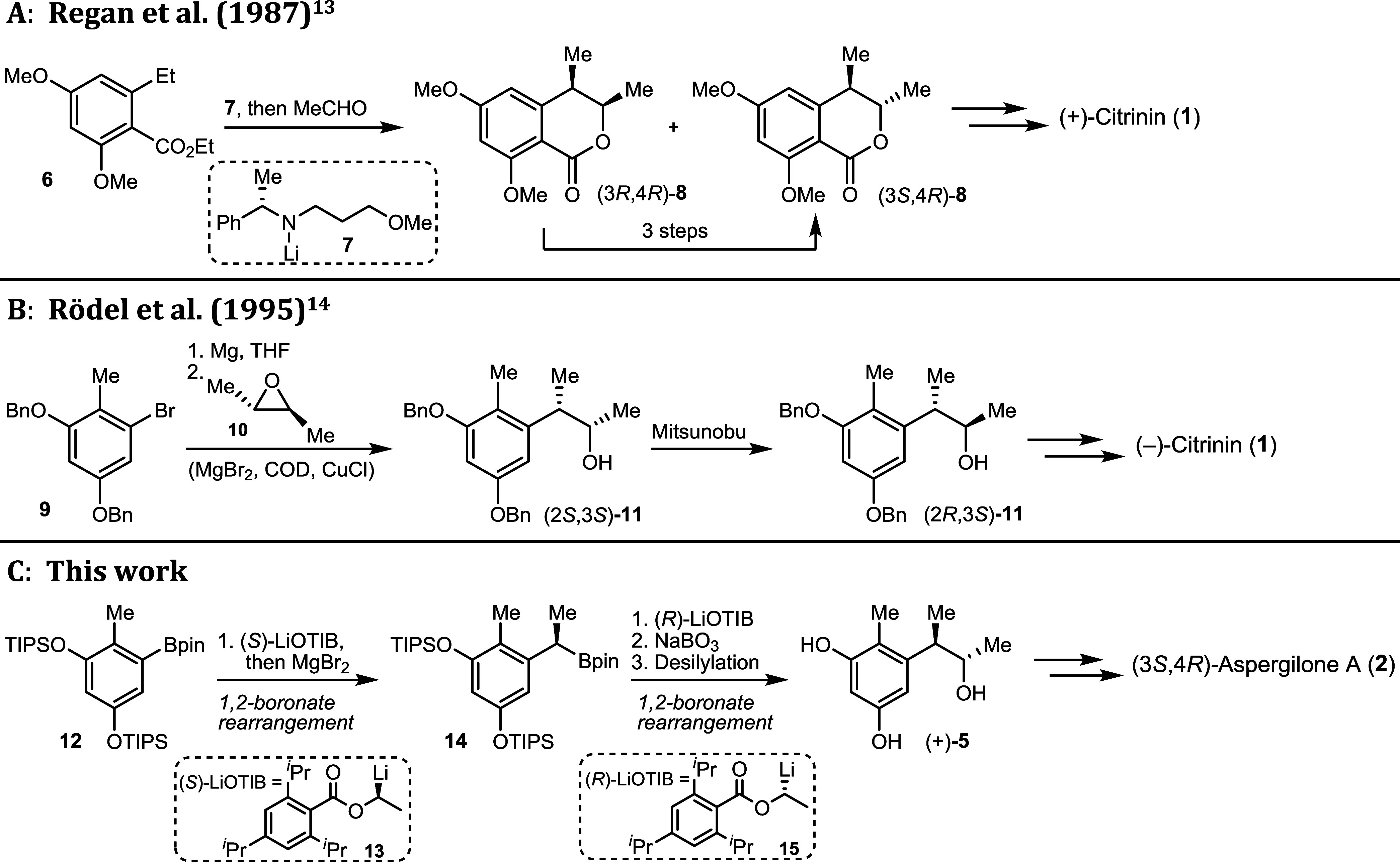
Introduction of the Stereogenic Centers in the Synthesis Towards (+)/(−)-Citrinin (**1**) and (*3S,4R*)-Aspergilone A (**2**)

Spurred by our interest in aspergilone A (**2**) due to its antifouling and anticancer activity,^3,17^ we wanted to develop a synthetic route with access to all its stereoisomers. Choosing phenol A (**5**) as the key intermediate, its stereocontrolled construction would presumably allow for the synthesis of a range of citrinin-type natural products and related structure–activity relationship studies.

Herein, we report the enantioselective synthesis of (3*S*,4*R*)-aspergilone A (**2**). The key steps were two successive stereospecific anionic 1,2-boronate rearrangements^18–20^ to furnish the privileged intermediate (+)-(2*R*,3*S*)-phenol A (**5**) (Scheme 1C). Due to reagent control, all stereoisomers are accessible without adjustments to the synthetic procedure potentially enabling access to all four stereoisomers of aspergilone A (**2**). For the naturally occurring (+)-**2**, the relative configuration had already been assigned as (3S*,4R*)^3^ but the absolute configuration was unknown. From the optical rotation of synthetic (3*S*,4*R*)-**2** we determine the absolute configuration of (+)-**2**.

## 2 Results and Discussion

### 2.1 Retrosynthesis

Our retrosynthetic analysis is shown in Scheme 2. We envisioned to synthesize (3*S*,4*R*)-aspergilone A (**2**) from (+)-(2*R*,3*S*)-phenol A (**5**) by formylation/condensation with an orthoformate and subsequent double benzylation. (+)-(2*R*,3*S*)-phenol A (**5**) could be derived from the aryl boronic acid pinacol ester **12**, using two sequential 1,2-boronate rearrangements, followed by oxidation of the boronic ester to the alcohol and removal of the silyl groups. Aryl boronic acid pinacol ester **12** should be attainable from the corresponding 1-bromo-3,5-dimethoxybenzene **16** in a five-step process consisting of formylation, deoxygenation, bis-*O*-demethylation, bis-*O*-silylation, and borylation with a boronic acid pinacol ester.

**Scheme 2:**
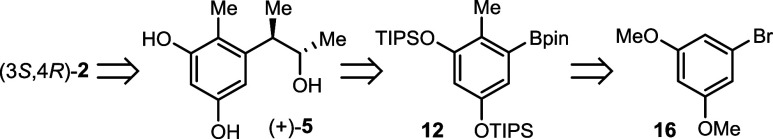
Retrosynthetic Analysis of (*3S,4R*)-Aspergilone A (**2**) and (*2R,3S*)-Phenol A (+)-(**5**)

### 2.2 Synthesis

We started our endeavor from commercially available 1-bromo-3,5-dimethoxybenzene **16** and employed a modified literature protocol for the Vilsmeier–Haack formylation (Scheme 3).^21^ We obtained the formylated product **17** in 90% yield. In the following Wolff–Kishner reduction, when employing toluene or PEG-400 ^22^ as solvents, either KOH or the starting material were insoluble, respectively, and low to no conversion was observed. Eventually we first made the hydrazone by mixing hydrazine and aldehyde **17** in toluene and then added KOH and PEG-400. Deoxygenated **18** was obtained in 99% yield. The amount of hydrazine and the reaction time could be greatly reduced by employing μ-wave irradiation.^23^ Standard bis-*O*-demethylation with BBr_3_ in DCM and installment of the TIPS groups toward **20**, using TIPS-Cl and imidazole in dry DCM, proceeded in high to excellent yields. Attempts to start the synthesis from 5-bromoresorcinol instead of **16**, thus avoiding the demethylation step, were unsuccessful. Formylation reactions in the presence of the free hydroxyl groups were low yielding or did not proceed at all when the TIPS groups were introduced first. Next, standard Miyaura borylation conditions of Pd­(dppf)­Cl_2_, bis­(pinacolato)­diboron (B_2_pin_2_) and potassium acetate were used to yield the desired aryl boronic ester **12** in 72%.^24^

**Scheme 3:**
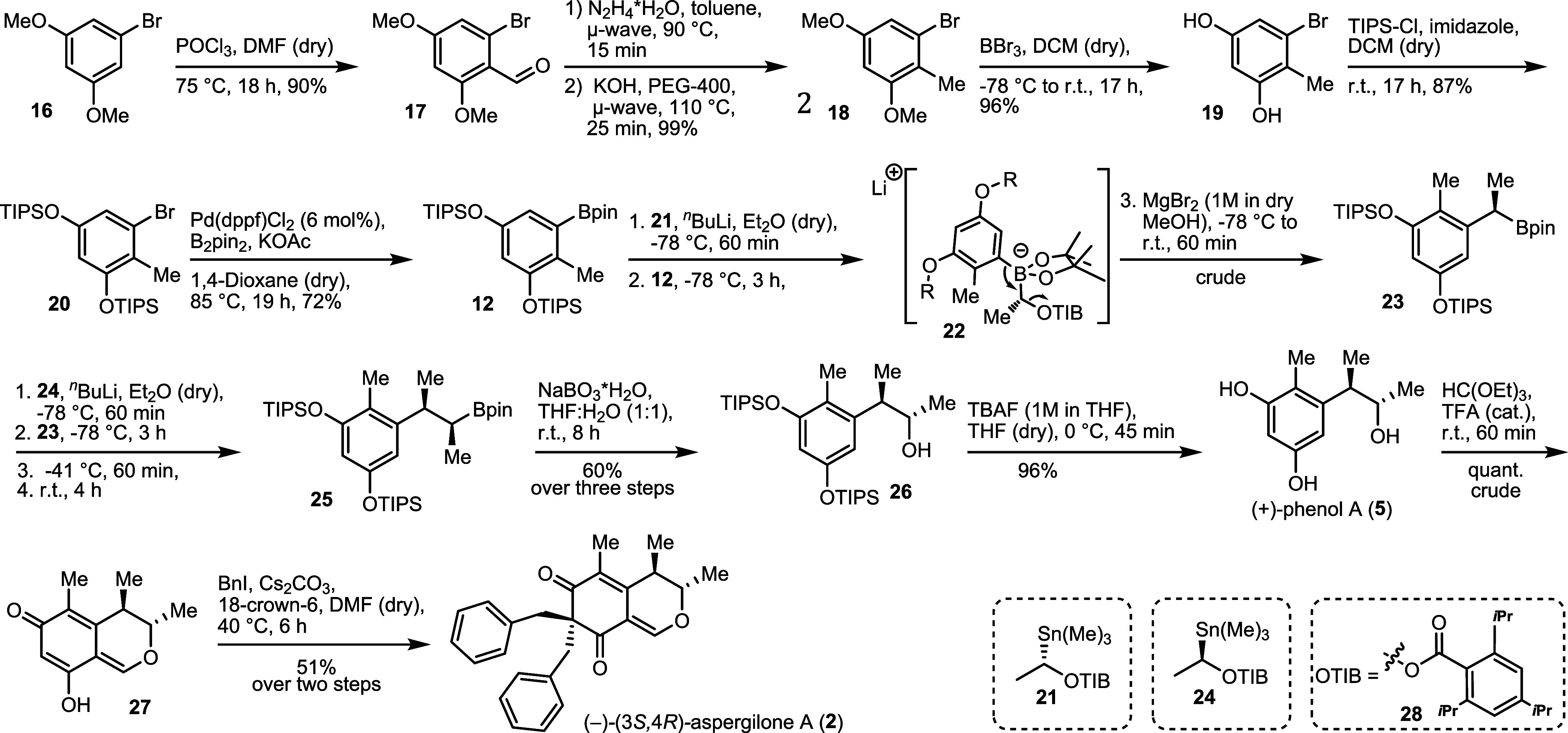
Synthetic Strategy Towards *ent*-Aspergilone A (**2**)

In the following key step, the stepwise, stereocontrolled introduction of the stereocenters, (*S*)-tin benzoate **21** was treated with *n*-BuLi at cryogenic temperatures to deliver the chiral secondary lithium alkyl **13** (Scheme 1).^25,26^ Upon addition of aryl boronic acid pinacol ester **12**, boronate complex **22** was formed. The latter underwent an anionic 1,2-aryl shift, eliminating the benzoate leaving group **28** and forming alkylboronic pinacol ester **23**. The necessary antiperiplanar arrangement of the migrating group and the benzoate dictates the stereochemical outcome.^27^ Aryl groups are known to have poor migration capabilities and sometimes require elevated temperatures and/or Lewis acids to promote the reaction.^20^ While the rearrangement took place at ambient temperature without any additive, the crude reaction mixtures were more complex, possibly due to partial decomposition of the boronate complex, rendering the step not fully reproducible. The addition of MgBr_2_ resolved this problem, while reducing the reaction time as well.^18^ Suspending the crude multiple times in *n-*heptane facilitated the removal of the Lewis acid and no further purification was necessary. Repeating the rearrangement procedure with (*R*)-tin benzoate **24** as a reagent, the second ethyl group was incorporated, delivering compound **25**. Since alkylboronic pinacol esters readily undergo the 1,2-rearrangement, no additional Lewis acid was needed for the second rearrangement.^20^ Oxidation of the boronic acid ester to the secondary alcohol with NaBO_3_ yielded pure *O*-TIPS protected (+)-phenol A **26** in 60% over three steps starting from **12**. During optimization of the conditions for the 1,2-boronate rearrangement, we prepared a mixture of all diastereomers of **26**. It is noteworthy to mention that the diastereomeric pairs of enantiomers (*S*,*R*)/(*R*,*S*)-**26** and (*S*,*S*)/(*R*,*R*)-**26** are separable by column chromatography on silica gel.

Removal of the silyl groups with tetrabutylammonium fluoride (TBAF) proceeded with excellent yields, delivering (+)-phenol A (**5**) in a total yield of 31% over 9 steps and with >99% ee of the single stereoisomer as determined by chiral SFC. Three enantioselective syntheses of phenol A (**5**) have been reported before, yielding 6% over 9 steps,^13^ 11% over 8 steps^14^ and 32% over 11 steps.^28^ While the latest synthesis provided the same yield,^28^ the stereocenters were installed in the first step, thus offering much less flexibility. Our compound exhibited a specific rotation of +34.5, which is close in value to phenol A: [α] = −36.4, but opposite in sign, as one would expect. We originally attempted to avoid introducing silyl protecting groups and proceed with the aryl methyl ethers present in compound **16** to shorten the synthesis, but the final removal of the methyl groups proved to be difficult (see Supporting Information).

To obtain the desired 3,4-dihydro-6*H*-isochromen derivative **27**, we used an excess of triethyl orthoformate and TFA. Similar conditions had been reported for the synthesis of citrinin (**1**),^29–31^ for which additional acid was not required, presumably due to their substrate bearing a carboxylic acid moiety. Compound **27** was obtained as a TFA adduct, purification of which proved to be difficult, thus the crude compound was used directly in the next step. Interestingly, (+)-phenol A (**5**) readily cyclized to the isochromane upon exposure to acetone, without the need of an acid catalyst, in contrast to previous reports.^29^

The final dialkylation was surprisingly challenging. Standard alkylation conditions using BnBr with carbonate bases in polar, aprotic solvents (DMF, acetone, 1,4-dioxane, DCM) led to low yields of 25% at best. We further explored addition of silver salts, phase transfer catalysts, hydroxide bases, hindered amine bases and Mitsunobu conditions, but to no avail. Stronger bases, such as LiOH and tetrabutylammonium hydroxide (TBAOH) yielded a strong signal of the target mass in HRMS, but no target compound could be isolated. Higher temperatures and longer reaction times also seemed to be counterproductive, presumably due to the relative instability of the deprotonated 3,4-dihydro-6*H*-isochromen **27**. We consequently theorized that the substrate needed to be consumed rather quickly at moderate temperatures to obtain acceptable yields. Eventually, using benzyl iodide, Cs_2_CO_3_ and 18-crown-6 in dry DMF at slightly elevated temperatures, (3*S*,4*R*)-aspergilone A (**2**) was isolated in 51% over two steps. Additionally, a minor amount of *O*-benzylated **27** was obtained in the last step.

The analytical data of (3*S*,4*R*)-(**2**) matched reported values, except for optical rotation, where we observed the same magnitude, yet the opposite sign.^3^ Based on these findings, we can establish that we synthesized the enantiomer (−)-aspergilone A of the natural product and that (+)-aspergilone A has an absolute configuration of (3*R*,4*S*).

## 3 Conclusion

In conclusion, we have developed the first enantioselective total synthesis of (−)-*ent-*aspergilone A (**2**) with a total yield of 15.7% and >99% ee. Additionally, we established the absolute configuration of naturally occurring (+)-aspergilone A (**2**) to be (3*R*,4*S*). The key transformation, a double anionic 1,2-boronate rearrangement, permits full stereocontrol and formal access to all stereoisomers of aspergilone A (**2**). Furthermore, this approach allows for the stereoselective synthesis of other citrinin type azaphilones, where phenol A (**5**) can be conceived as an intermediate.

## Supplementary Material



## Data Availability

The data underlying this study is available in the published article, in its Supporting Information, and openly available in DataverseNO at 10.18710/NWIK1U

## Notes

NMR data comparison for natural and synthetic aspergilone A, a description of additional synthetic attempts, experimental and analytical data for synthesized compounds, and copies of NMR and IR spectra (PDF)
