# Supplementary material for: Stereocontrolled and Adaptable Synthesis of (−)-Aspergilone A via the Key Intermediate (+)-Phenol A
Source: J Org Chem. 2025 Sep 11;90(38):13597–601. doi: 10.1021/acs.joc.5c01650 (PMC12481563; doi:10.1021/acs.joc.5c01650)
Supplement: Supplementary file 1 [file jo5c01650_si_001.pdf]

Supporting Information
for
Stereocontrolled and Adaptable Synthesis of (–)-Aspergilone A via the Key Intermediate (+)-Phenol A

Manuel K. Langer[a], Annette Bayer[a]*
[a] *Department of Chemistry, UiT – The Arctic University of Norway, NO-9037 Tromsø, NORWAY.*
**Corresponding author; annette.bayer@uit.no*

Table of Contents

1. Spectral comparison of aspergilone A (**2**) S2
2. Screening of conditions S3
3. Experimental Procedures S5
   3.1 General Information S5
   3.2 Materials and reagents S6
   3.3 Synthesis of building blocks S6
   3.4 Synthesis of (–)-(3*S*,4*R*)-aspergilone A (**2**) S8
   3.5 Synthesis of other compounds and racemic mixtures S15
4. NMR spectra S19
5. IR Spectra S36
6. References S40

1. Spectral comparison of aspergilone A (**2**)

Table S1. Comparison of the ^{1}H NMR data of natural (+)-(3*S*,4*R*)-aspergilone A (**2**) (acetone-*d6*)[1] and our synthetic (–)-(3*R*,4*S*)-aspergilone A (**2**) (chloroform-*d*).

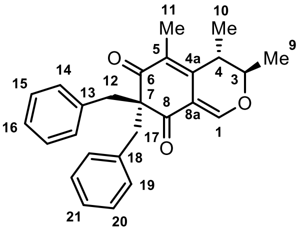

(+)-(3*S*,4*R*)-Aspergilone A

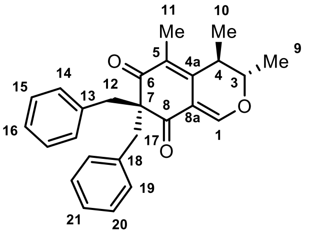

(–)-(3*R*,4*S*)-Aspergilone A

Position	^{1}H NMR		^{13}C{^{1}H} NMR	
	Natural (600 MHz) δ$_H$ [ppm, mult, *J* (Hz)]	Synthetic (400 MHz) δ$_H$ [ppm, mult, *J* (Hz)]	Natural (150 MHz) δ$_C$ [ppm, mult]	Synthetic (101 MHz) δ$_C$ [ppm, mult]
1	7.48, s	7.50, s	155.1, –C	154.6, –C
2	–	–	–	–
3	4.32, dq (6.6, 0.8)	4.19, q (6.6)	79.3, CH	78.5, CH
4	2.58, dq (7.0, 0.8)	2.35, q (7.1)	30.1, CH	34.7, CH
4a	–	–	145.2, –C	144.2, –C
5	–	–	126.5, –C	126.1, –C
6	–	–	200.3, –C	200.4, –C
7	–	–	66.5, –C	66.3, –C
8	–	–	199.3, –C	199.5, –C
8a	–	–	111.8, –C	111.0, –C
9	0.71, d (6.6)	0.68, d (6.6)	18.0, CH_3	18.0, CH_3
10	0.55, d (7.0)	0.54, d (7.1)	16.9, CH_3	16.8, CH_3
11	1.59, s	1.60, s	9.5, CH_3	9.6, CH_3
12	3.28, d (12.0)	3.37, d (12.4)	47.0, CH_2	46.1, CH_2
	3.14, d, (12.0)	3.23, d (12.4)		
13	–	–	138.0, –C	136.6, –C
14	6.89-6.87, m	6.97-6.91, m	130.5, CH	129.8, CH
15	7.11-7.06, m	7.13-7.02, m	127.2, CH	128.0, CH
16	7.11-7.06, m	7.13-7.02, m	128.6, CH	126.5, CH
17	3.36, d (12.6)	3.46, d (12.6)	45.6, CH_2	45.1, CH_2
	3.22, d (12.6)	3.30, d (12.6)		
18	–	–	137.6, –C	137.0, –C
19	6.84-6.82, m	7.02-6.97, m	130.3, CH	130.0, CH
20	7.11-7.06, m	7.13-7.02, m	127.1, CH	128.0, CH
21	7.11-7.06, m	7.13-7.02, m	128.8, CH	126.4, CH

2. Screening of conditions

In an earlier attempt to use methyl as the protecting group we screened various conditions for its removal. Commonly, cleavage of phenolic *O*-methyl ethers is achieved with strong Lewis acids, such as BBr_3, $AlCl_3$ with NaI or TMSI.[2, 3] However, acidic conditions can induce racemization of bis-*O*-methylate (+)-phenol A (**S1**) by means of a Wagner-Meerwein rearrangement proceeding through phenonium ion **S14** formed through water elimination.[4, 5] To get an understanding of the rate of racemization, we treated alcohol (+)-**S1** with a 2 N H_2SO_4 solution at ambient temperature (Scheme S1). After 3 h the optical purity was reduced to 74% ee, confirming that acidic conditions should be avoided in the presence of the secondary alcohol.

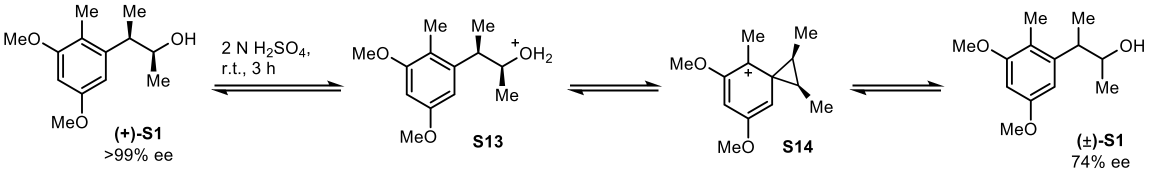

Scheme S1. Racemization studies of (+)-**S1**.

Therefore, we considered to do the acidic conditions before the oxidation of the boronic acid pinacol ester to the secondary alcohol and decided to employ aryl boronic ester **S2** as a model system (Table S2). We employed a wide variety of conditions, some of which are summarized in Table S2. Standard reagents such as BBr_3, $AlCl_3$ and TMSI (entries 1-5) either yielded complex mixtures or only affected the boronic acid ester, not cleaving the phenyl methyl ethers. Earlier reports showed that the $AlCl_3$ – *N,N*-dimethylaniline (*N,N*-DMA) system is effective in cleaving benzyl and allyl ethers[6], which was later extended to (poly) aryl methyl ethers.[7]

Table S2. Screening of acidic rection conditions for simultaneous bis-*O*-demethylation and boronic acid ester cleavage.

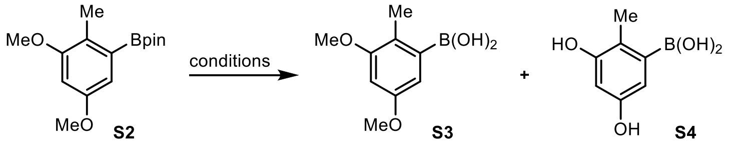

Entry	Reagents (eq)	Solvent	Δ [°C]	Time [min]	Observation
1	BBr_3 (4.0)	DCM, dry	-78	150	Complex mixture
2	$AlCl_3$ (3.5)	DCM, dry	25-40	23 h	Only **S3**[a]
3	$AlCl_3$ (4.0), NaI (4.0)	DCM, dry	40	10 h	Only **S3**[a]
4	TMSI (5.0)	MeCN, dry	120 (µ-wave)	30	Complex mixture
5	TMSI (5.0)	MeCN, dry	80	120	Only **S3**[a]
6	$AlCl_3$ (9.0), *N,N*-DMA (9.0)	Toluene, dry	115	18 h	**S3** in a complex mixture
7	$AlCl_3$ (9.0), *N,N*-DMA (9.0)	Toluene, dry	115	1 h	**S4** in small amounts
8	$AlCl_3$ (9.0), *N,N*-DMA (9.0)	Toluene, dry	160 (µ-wave)	15	~33% **S4**[a]
9	$AlCl_3$ (5.0), *N,N*-DMA (5.0)	Toluene, dry	160 (µ-wave)	10	Not fully converted
10	$AlCl_3$ (9.0), *N,N*-DMA (9.0)	Toluene, dry	160 (µ-wave)	5	~40% **S4**[a]
11	$AlCl_3$ (9.0), *N,N*-DMA (9.0)	Toluene, dry	100 (µ-wave)	5	No pdct formation
12	$AlCl_3$ (9.0), *N,N*-DMA (9.0)	Toluene, dry	120 (µ-wave)	5	~30% **S4**[a]
13	$AlCl_3$ (9.0), DIPEA (9.0)	Toluene, dry	160 (µ-wave)	5	No pdct formation
14	$AlCl_3$ (9.0), pyridine (9.0)	Toluene, dry	160 (µ-wave)	5	~61% **S3**[a]
15	$AlCl_3$ (9.0), 4-DMAP (9.0)	Toluene, dry	160 (µ-wave)	5	~91% **S3**[a]
16	$AlCl_3$ (9.0), *N*-MA (9.0)	Toluene, dry	160 (µ-wave)	5	~35% **S4**[a]
17	$AlCl_3$ (9.0), Aniline (9.0)	Toluene, dry	160 (µ-wave)	10	~28% **S4**[a]

N,N-DMA: *N,N*-dimethylaniline. *N*-MA: *N*-methylaniline. [a]Yield estimated by qNMR with methyl-3,5-dinitrobenzoate.

Our first tries led to a complex mixture (entry 6) and only small amounts of the desired product **S4** (entry 7). But increasing the temperature and switching to microwave irradiation, thus reducing the reaction time led to 33% of crude yield (entry 8). Reducing either the amounts of the reagents (entry 9) or the temperature (entries 11 and 12) led to lower crude yields or no conversion at all. The best results were obtained when the reaction time was shortened to 5 min (entry 10), but the crude yield did not exceed 40%. Changing the amine (entries 13-17) did not improve the crude yields.

As acidic conditions did not work satisfactorily on our model system, we wanted to investigate basic ether cleavage and chose benzyl alcohol **S5** as a model system (Table S3). First, we investigated L-selectride[8] and a mixture of 2-diethylaminoethanethiol hydrochloride/KO^{t}Bu[9] (both not shown), but obtained only the starting material and mono *O*-demethylated **S6**. Both reagents suffer from the same drawback, namely, that after full deprotection a tri-anionic species would be obtained, which did not seem feasible. We were intrigued when coming across a report about counterattack reagents for bis-*O*-demethylation[10] employing bis(trimethylsilyl)sulfide [S(TMS)$_2$]. Mechanistically, a hydroxyl group in the substrate is deprotonated and the hydroxylate can then attack one of the silicon atoms in S(TMS)$_2$, leading to a TMS protected alcohol and liberation of a sulfur nucleophile. The latter can remove a methyl group from the substrate and the resulting phenolate can in turn attack another TMS group, yielding a multiple TMS protected product, thereby avoiding multiple negative charges on one molecule. Using reported conditions, we obtained an isolated yield of 48% in our first test reaction (Table 3, entry 1). Due to the required high temperatures we decided to switch again to microwave irradiation, leading to similar crude yields, but much shorter reaction times (entries 3-5). Adjusting the amounts of the reagents (entries 6-7) did not improve the crude yields and neither did using LDA (entry 8) as a base. But when NaHMDS was employed (entry 9) the crude yield rose to 67%. Adjusting the reaction time and the amount of base we obtained **S7** as the sole product in 76% crude yield (entry 11). Elated by those findings, we used the optimized conditions on (+)-**S1** but could only obtain 33% (55% brsm) of (+)-phenol A (**5**), alongside with starting material and mono-*O*-demethylated product. Based on these findings, we decided to change our protecting group strategy.

Table S3. Screening of reaction conditions for bis-*O*-demethylation with bis(trimethylsilyl)sulfide [S(TMS)$_2$].

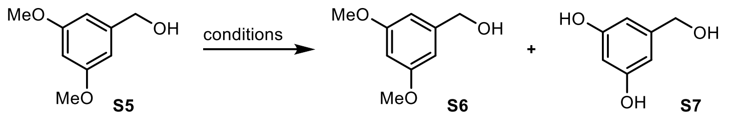

Entry	Base (eq)	S(TMS)$_2$ [eq]	Solvent	Δ [°C]	Time [min]	S6:S7[a]
1	NaH (1.0)	2.0	DMI, dry	190	36 h	48%[b]
2	NaH (1.5)	1.5	DMI, dry	190	21 h	32%[b]
3	NaH (1.8)	1.75	DMI, dry	220 (μ-wave)	10	1.0:3.0, 53%[c]
4	NaH (1.8)	1.75	DMI, dry	190 (μ-wave)	30	1.0:1.4, 50%[d]
5	NaH (1.8)	1.75	DMI, dry	210 (μ-wave)	22	1.0:5.0, 53%[d]
6	NaH (1.4)	1.5	DMI, dry	220 (μ-wave)	20	1.0:2.0, 32%[d]
7	NaH (2.2)	2.2	DMI, dry	220 (μ-wave)	15	1.0:4.4, 35[d]
8	LDA (1.5)	1.75	DMI, dry	220 (μ-wave)	10	1.0:0.3, 15%[d]
9	NaHMDS (1.35)	1.75	DMI, dry	220 (μ-wave)	20	1.0:10.2, 67%[d]
10	NaHMDS (1.35)	2.0	DMI, dry	220 (μ-wave)	20	1.0:20, 77%[d]
11	NaHMDS (1.10)	2.0	DMI, dry	220 (μ-wave)	25	0:100, 76%[d]

S(TMS)$_2$: Bis(trimethylsilyl)sulfide. LDA: Lithium diisopropylamide. NaHMDS: Sodium bis(trimethylsilyl)amide. DMI: 1,3-Dimethyl-2-imidazolidinone. [a]Percentages relate to the yield of **S7** in the mixtures, as determined by qNMR. [b]isolated yield. [c]qNMR with (2,4,6-triisopropylbenzoic acid). [d]qNMR with methyl-3,5-dinitrobenzoate.

3. Experimental Procedures

3.1 General Information

Air and water sensitive reactions were carried out in heat-dried glassware under an argon atmosphere using standard Schlenk techniques. Anhydrous solvents were prepared from commercially available solvents and dried over molecular sieves of appropriate pore size.

Normal phase flash chromatography was carried out on VWR Silica Gel 40 – 63 µm. Automated flash chromatography was carried on an interchim® PuriFlash XS420 flash system with the sample preloaded on a Samplet® cartridge belonging to a Biotage SP-1 system or on a Biotage® Isolera One system with the sample preloaded on Celite. For both systems Biotage Sfär Silica columns were used. Thin layer chromatography was carried out using Merck TLC Silica gel 60 F_{254} and visualized by short-wavelength ultraviolet light or by treatment with an appropriate stain.

NMR spectra were obtained on a Bruker Avance 400 MHz spectrometer at 20 °C. The chemical shifts (δ) are reported in parts per million (ppm) relative to the solvent residual peak (Chloroform-*d*: δH 7.26 and δC 77.16; Acetone-*d6*: δH 2.05 and δC 29.84 or 206.26). ^{13}C-NMR spectra were obtained with ^{1}H decoupling. Data is represented as follows: chemical shift, multiplicity (s = singlet, d = doublet, t = triplet, q = quartet, dd = doublet of doublet, dq = doublet of quartet, bs = broad singlet, m = multiplet), coupling constant (J, Hz) and number of protons.

High-resolution mass (HRMS) spectra were recorded from methanol solutions on a LTQ Orbitrap XL (Thermo Scientific) with electrospray ionization (ESI), a Waters Xevo G2-XS QTof with electrospray ionization (ESI), Thermo Scientific Q Exactive GC Orbitrap with electron ionization (EI) and chemical ionization (CI) or a ThermoScientific Vanquish UHPLC system coupled to a ThermoScientific Orbitrap Exploris 120 with electrospray ionization (ESI). Ultrapure MilliQ Water and HPLC grade solvents were used for the Vanquish UHPLC system. Water (A) and a 90/10/0.1 (v/v) mixture of MeCN/H_2O/formic acid (B) were used as solvents. Gradient: 0-70% (B) over 7 min, 100% (B) for 2 min, 0% (B) for 2 min. The molecular ion peaks are reported as molecular ion plus hydrogen $[M+H]^+$ or molecular ion $[M]^+$.

Infra-red (IR) spectra of the pure compounds were recorded on an Agilent Technologies Cary 630 FTIR. Selected absorption maxima (ν_{max}) are reported in wavenumbers (cm^{-1}).

Melting points were measured in degree Celsius (°C) using a Stuart SMP50 automatic melting point detector and are reported uncorrected.

Optical rotations were determined on an Optical Activity LTD AA-10R Automatic Polarimeter at 20 °C in a cuvette of 100 mm length with the sodium D-line (589 nm). The concentrations and solvents (HPLC grade) were adapted for each compound individually. The polarimeter was calibrated against the pure solvent. Concentrations are given in g/(100 mL).

Chiral HPLC measurements were performed on a Shimadzu apparatus. The different modules were SIL-10ADVP (auto injector), LC-10ATVP (liquid chromatograph), FCV-10ALVP (pump), DGU-14A S2 (degasser), CTO-10ASVP (column oven), SCL-10AVP (system controller) and SPD-M10A (diode array detector). HPLC grade *n*-hexane and isopropanol were used as solvents. Separation was achieved on a Chiralcel OD-H S-5µm (0.46 cm Ø×25 cm) column.

Chiral SFC was performed on a Waters ACQUITY UPC^2 system equipped with a Trefoil™ CEL2, 2.5 µm, 3.0 x 150mm or a Trefoil™ CEL1, 2.5 µm, 3.0 x 150mm column coupled to a Waters ACQUITY PDA detector spanning from wavelengths 205 to 650 nm. HPLC grade solvents and supercritical CO_2 were used as the mobile phase. Solvent mixtures and gradients are specified for each compound individually.

3.2 Materials and reagents

Unless otherwise noted, purchased chemicals were used as received without further purification. Anhydrous Et_2O was dried over two columns of activated alumina[11] in a solvent purification system (SPS) and stored over 3 Å molecular sieves. Commercially available MCPE (anhydrous, inhibitor free) was stored over 4 Å molecular sieves for at least 3 days before usage. Other anhydrous solvents were prepared according to standard procedures, using the appropriate size of molecular sieves. Solvents were degassed by three rounds of the freeze-pump-thaw method. TMEDA, (–)-sparteine and (+)-sparteine were distilled over CaH_2 and stored under N_2 at -20 °C. The 1 M solution of $MgBr_2$ in anhydrous MeOH was prepared according to literature procedures[12] and stored at -20 °C.

3.3 Synthesis of building blocks

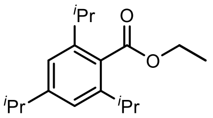

Ethyl 2,4,6-triisopropylbenzoate **S8**.[13]

A biphasic mixture of 2,4,6-isopropylbenzoic acid (10 g, 40.3 mmol, 1.0 eq), tetrabutylammonium iodide (892 mg, 2.42 mmol, 0.06 eq), NaOH (3.54 g, 88.6 mmol, 2.20 eq) and 1-bromoethane (15.8 mL, 221 mmol, 5.25 eq) in $CHCl_3$ (200 mL) and water (160 mL) was stirred vigorously for 5 d at ambient temperature. The layers were separated and the aqueous layer was extracted with DCM twice. The combined organics were dried over $MgSO_4$, filtered and the solvent was removed under reduced pressure. *n*-Pentane was added to the resulting clear liquid, upon which a white precipitated formed. The solids were filtered off and washed with *n*-pentane twice. The filtrates were combined and the solvent was removed under reduced pressure to yield **S8** (11.0 g, 40.0 mmol, 99%) as a clear colourless liquid.

^{1}H NMR (400 MHz, Chloroform-*d*) δ 7.00 (s, 2H), 4.37 (q, *J* = 7.1 Hz, 2H), 2.88 (dq, *J* = 13.7, 7.0 Hz, 3H), 1.37 (t, *J* = 7.2 Hz, 3H), 1.25 (d, *J* = 6.8 Hz, 12H), 1.24 (d, *J* = 7.0 Hz, 6H).

^{13}C{^{1}H} NMR (101 MHz, Chloroform-*d*) δ 171.0, 150.2, 144.9 (2C), 130.8, 121.0 (2C), 60.9, 34.6, 31.6 (2C), 24.3 (4C), 24.1 (2C), 14.4.

HRMS: (ESI): calcd for $C_{18}H_{29}O_2^+$ $[M+H]^+$ 277.2162, found: 277.2158.

IR (ν_{max}/cm^{-1}, neat): 2960, 1724, 1250, 1075, 765.

R$_f$: 0.58 (10% EtOAc in heptane).

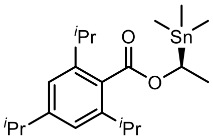

(S)-1-(trimethylstannyl)ethyl 2,4,6-triisopropylbenzoate **21**.[13]

S8 (5.00 g, 18.1 mmol, 1.0 eq) and freshly distilled (–)-sparteine (5.4 mL, 23.5 mmol, 1.3 eq) were added to a flame dried reaction vessel under argon atmosphere. Anhydrous Et_2O (72 mL) was added, and the mixture was cooled to -78 °C. After 10 min equilibration time, *sec*-BuLi (18.1 mL, 25.3 mmol, 1.4 eq) was added dropwise *via* a syringe over 10 min. The mixture turned red and then brown. Stirring was continued for 4 h at that temperature, during which a precipitate formed. Then Me_3SnCl (23.5 mL, 23.5 mmol, 1.3 eq, 1 M in hexanes) was added dropwise over 30 min and stirring was continued at -78 °C for 20 min. The solution was allowed to warmed to ambient temperature and stirred for 80 min at that temperature. A 5% $H_3PO_{4(aq)}$ solution was added and the biphasic mixture was stirred for 10 min. The layers were separated and the organic layer was washed twice with a 5% $H_3PO_{4(aq)}$ solution. The combined aqueous layers were extracted with Et_2O thrice and the combined organics were dried over $MgSO_4$, filtered and the solvent was removed under reduced pressure. (*S*)-Stannane **21** (5.3 g, 12.1 mmol, 67%) was obtained as a white solid after recrystallization.

> ***Caution!** Trimethyltin chloride is classified as being Category 1 acutely toxic (H310: Fatal in contact with skin). It must be handled with extreme care and the appropriate PPE to avoid direct exposure.*

Caution! Sec-butyllithium is extremely pyrophoric. It must be handled using proper needle and syringe techniques.

The crude was recrystallized from MeOH (2-3 mL/g crude). After solvent addition the mixture was heated until all solids dissolved. After crystallization, the solids were collected and the residual MeOH left for further crystallization. Two crops were obtained. The crops were combined and recrystallized once more from MeOH to yield 3 crops. Each crop was kept and used individually.

^{1}H NMR (400 MHz, Chloroform-*d*) δ 7.00 (s, 2H), 5.04 (q, J = 7.6 Hz and q, J = 7.6 Hz, 1H), 2.94 – 2.79 (m, 3H), 1.59 (d, J = 7.6 Hz and dd, J = 54.7, 7.7 Hz and dd, J = 57.8, 7.7 Hz, 2H), 1.24 (d, J = 6.8 Hz, 18H), 0.18 (s, 9H and d, J = 51.7 Hz and J = 54.1 Hz).

^{13}C{^{1}H} NMR (101 MHz, Chloroform-*d*) δ 171.4, 150.1, 145.0 (2C), 130.9, 120.9 (2C), 67.2, 34.5, 31.5, 24.5 (2C), 24.2 (2C), 24.1 (2C), 19.4, -8.1, -8.2, -9.8 (s and d, J = 320.3 Hz and d, J = 335.4 Hz, 1C).

HRMS (ESI): calcd for $C_{21}H_{37}O_2{}^{120}Sn^+$ $[M+H]^+$ 441.1810, found: 441.1802.

IR (ν_{max}/cm^{-1}, neat): 2959, 1696, 1259, 1074, 765.

M$_p$: 48-51 °C.

R$_f$: 0.40 (5% EtOAc in heptane).

$[\alpha]_D^{20}$: +37.1° (*c* 2.4, $CHCl_3$ /w 0.6% EtOH).

Chiral HPLC (Daicel Chiralcel OD-H, hexane, 0.9 mL/min, room temperature, 211 nm): t_R = 4.4 min (*S*-**21**), e.r. = 99.9:0.1; t_R = 7.9 min (*R*-**24**), e.r. = 99.8:0.2 for the first crop.

For the 2nd crop: *not determined.*
For the 3rd crop: (*S*), e.r. = 98.9:1.1, (*R*), e.r. = 98.2:1.8.

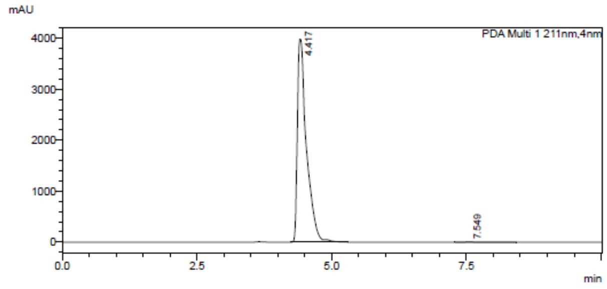

Peak Table

PDA Ch1 211nm

Peak#	Ret. Time	Area	Height	Area%
1	4.417	46886107	3984164	99.875
2	7.549	58530	3375	0.125
Total		46944636	3987539	100.000

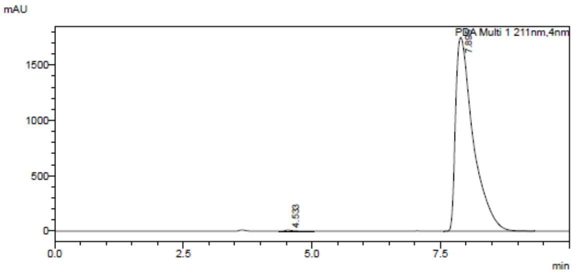

Peak Table

PDA Ch1 211nm

Peak#	Ret. Time	Area	Height	Area%
1	4.533	88342	9341	0.211
2	7.895	41714152	1748893	99.789
Total		41802494	1758234	100.000

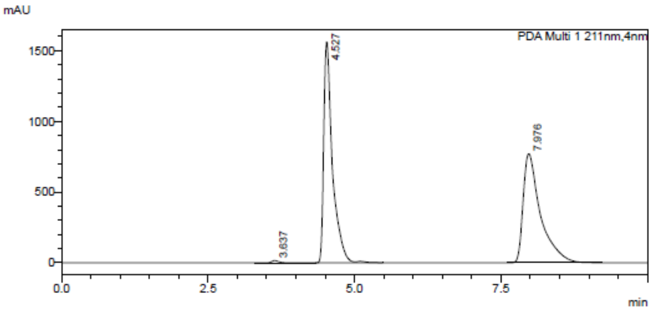

(*R*)-1-(trimethylstannyl)ethyl 2,4,6-triisopropylbenzoate **24** and the racemic compound **S9** were obtained by the same procedure using (+)-sparteine and TMEDA, respectively.

Preparation of $MgBr_2$ in anhydrous MeOH[12]

Commercially available anhydrous $MgBr_2$ (Sigma-Aldrich) was placed in a heat dried round bottom flask together with a stirring bar. The flask was evacuated and backfilled with argon. The solids were heated to 200 °C under vacuum (1 mbar) for 3 h. The solids were allowed to cool to ambient temperature and anhydrous MeOH was added until a final concentration of 1 M was reached. Caution: Methanol starts boiling at the beginning of the addition due to strong heat evolution. A slightly grey solution was obtained with some precipitation. The solution was stored at -20 °C. Before usage, the flask was allowed to warm to room temperature and only the clear solution was used, carefully avoiding the solids.

3.4 Synthesis of (−)-(3*S*,4*R*)-aspergilone A (**2**)

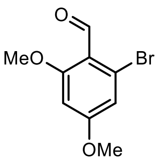

2-bromo-4,6-dimethoxybenzaldehyde **17**[14]

$POCl_3$ (13.30 mL, 142.6 mmol, 6.0 eq) was added to anhydrous DMF (27.6 mL, 357 mmol, 15.0 eq) at 0 °C to yield a white slurry. Solid 1-bromo-3,5-dimethoxybenzene **16** (5.16 g, 23.8 mmol, 1.0 eq) was added in one portion and the mixture was warmed to 75 °C in an oil bath for 18 h. The colour changed to dark red during the course of the reaction. The solution was cooled to 0 °C and a 2 N $NaOH_{(aq)}$ solution was added slowly. Upon neutralization a grey/white solid formed. The suspension was filtered and the residue was washed with water. The solids were collected and dried under high vacuum to yield **17** (5.23 g, 21.4 mmol, 90%) as a slightly grey solid.

^{1}H NMR (400 MHz, Chloroform-*d*) δ 10.28 (s, 1H), 6.75 (d, *J* = 2.3 Hz, 1H), 6.41 (d, *J* = 2.3 Hz, 1H), 3.87 (s, 3H), 3.85 (s, 3H).

^{13}C{^{1}H} NMR (101 MHz, Chloroform-*d*) δ 189.4, 164.6, 163.8, 127.7, 117.1, 111.7, 98.4, 56.3, 56.0.

HRMS (ES): calcd for $C_9H_{10}{}^{79}BrO_3^+$ $[M+H]^+$ 244.9808, found: 244.9802.

IR (ATR, v_{max}/cm^{-1}): 2872, 2780, 1082, 1590, 1556, 1450, 1413, 1229, 1203, 1136, 1051, 1026, 933, 826, 780.

M_p: 89 – 90 °C.

R_f: 0.53 (50% EtOAc in *n*-heptane).

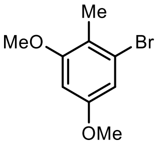

1-bromo-3,5-dimethoxy-2-methylbenzene **18**[15]

The following reaction was divided into six equal batches. The numbers refer to the total amount of reactants and reagents used.

To aldehyde **17** (5.21 g, 21.2 mmol, 1.0 eq) in toluene (48 mL) hydrazine hydrate (3.24 mL, 53.1 mmol, 2.50 eq; 80% in water) was added and the resulting mixture was heated to 90 °C for 15 min under μ-wave irradiation. Then PEG-400 (48 mL) and KOH (7.0 g, 127.5 mmol, 6.0 eq) were added and the mixture was heated to 110 °C for 25 min under μ-wave irradiation. Water was added and the layers were separated. The aqueous layer was extracted with toluene twice more. The combined organics were dried over Na_2SO_4, filtered and the solvent was removed under reduced pressure. The title compound **18** (4.86 g, 21.0 mmol, 99%) was obtained as a slightly yellow solid.

> ***Caution!*** *Nitrogen gas is released during the reaction. Microwave reactions proceed in a close system and the amount of nitrogen gas released should by calculated for each batch, to not exceed 50% of the maximum pressure rating for the given set-up.*

^{1}H NMR (400 MHz, Chloroform-*d*) δ 6.70 (d, *J* = 2.4 Hz, 1H), 6.38 (d, *J* = 2.6 Hz, 1H), 3.79 (s, 3H), 3.77 (s, 3H), 2.22 (s, 3H).

^{13}C{^{1}H} NMR (101 MHz, Chloroform-*d*) δ. 158.9, 158.7, 125.6, 119.4, 108.4, 98.1, 55.9, 55.7, 15.1.

HRMS (EI): calcd for $C_9H_{11}{}^{79}BrO_2^+$ $[M]^+$ 229.9937, found: 229.9939.

IR (ATR, v_{max}/cm^{-1}): 2921, 2835, 1606, 1571, 1487, 1406, 1372, 1320, 1219, 1148, 1036, 933, 828, 815, 793, 747, 699.

M$_p$: 29 – 31 °C.

R$_f$: 0.50 (15% EtOAc in *n*-heptane).

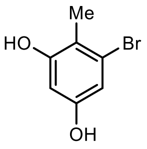

5-bromo-4-methylbenzene-1,3-diol **19**

Aryl bromide **18** (3.70 g, 16.0 mmol, 1.0 eq) was dissolved in dry DCM (30 mL) under inert atmosphere. The obtained solution was cooled to -78 °C and BBr_3 (38.5 mL, 38.5 mmol, 2.4 eq, 1 M solution in DCM) was added *via* a syringe pump (1 mL/min). The solution darkened during the addition and a solid formed, which dissolved by the end of the addition process. The resulting mixture was stirred for 17 h, during which it reached ambient temperature. The red/brown solution was then cooled to 0 °C and water was added slowly. After no more reaction was observed, the mixture was basified with a 2 N $NaOH_{(aq)}$ solution. The layers were separated and the organic layer was extracted with 1 N $NaOH_{(aq)}$ twice more. The combined aqueous layers were acidified to pH = 1 with $HCl_{(conc)}$ and then extracted with Et_2O thrice. The combined organics were dried over Na_2SO_4, filtered and the solvent was removed under reduced pressure. The title compound **19** (3.12 g, 15.4 mmol, 96%) was obtained as a red-brown solid.

^{1}H NMR (400 MHz, Acetone-*d6*) δ 8.54 (s, 1H), 8.31 (s, 1H), 6.60 (d, *J* = 2.3 Hz, 1H), 6.41 (d, *J* = 2.4 Hz, 1H), 2.17 (s, 3H).

^{13}C{^{1}H} NMR (101 MHz, Acetone-*d6*) δ 157.4, 157.3, 126.0, 116.2, 111.4, 102.8, 15.0.

HRMS (ESI): calcd for $C_7H_6{}^{79}BrO_2^-$ $[M\text{-}H]^-$ 200.9557, found: 200.9557.

IR (ATR, v_{max}/cm^{-1}): 3232, 1602, 1507, 1439, 1382, 1287, 1193, 1124, 1017, 977, 822, 759.

M$_p$: 117 – 120 °C.

R$_f$: 0.26 (35% EtOAc in *n*-heptane).

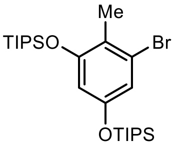

((5-bromo-4-methyl-1,3-phenylene)bis(oxy))bis(triisopropylsilane) **20**

Compound **19** (703 mg, 3.46 mmol, 1.0 eq) and imidazole (943 mg, 13.8 mmol, 4.0 eq) were dissolved in dry DCM (17 mL) under inert atmosphere. To the orange/red solution TIPS-Cl (1.85 mL, 8.66 mmol, 2.5 eq) was added and the resulting mixture was stirred at ambient temperature for 17 h. The mixture was diluted with DCM and the organic layer was washed with water twice and with brine once. The organic layer was dried over Na_2SO_4, filtered and the solvent was removed under reduced pressure to yield a yellow liquid. The crude was purified by column chromatography on silica gel with *n*-heptane to yield the title compound **20** (1.55 g, 3.0 mmol, 87%) as a colourless liquid.

^{1}H NMR (400 MHz, Chloroform-*d*) δ 6.73 (d, *J* = 2.4 Hz, 1H), 6.33 (d, *J* = 2.4 Hz, 1H), 2.24 (s, 3H), 1.34 – 1.15 (m, 6H), 1.10 (d, *J* = 7.3 Hz, 24H), 1.08 (d, *J* = 7.1 Hz, 12H).

^{13}C{^{1}H} NMR (101 MHz, Chloroform-*d*) δ 155.0, 154.6, 125.4, 121.7, 117.0, 109.7, 18.2 (6C), 18.0 (6C), 16.1, 13.2 (3C), 12.8 (3C).

HRMS (ESI): calcd for $C_{25}H_{48}{}^{79}BrO_2{}^{28}Si_2^+$ $[M+H]^+$ 515.2371, found: 515.2375.

IR (ATR, v_{max}/cm^{-1}): 2946, 2867, 1597, 1555, 1466, 1413, 1315, 1202, 1154, 1031, 913, 884, 847, 811, 758.

R$_f$: 0.57 (*n*-heptane).

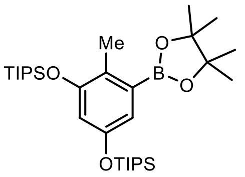

((4-methyl-5-(4,4,5,5-tetramethyl-1,3,2-dioxaborolan-2-yl)-1,3-phenylene)bis(oxy))bis(triisopropylsilane) **12**

An oven dried flask was charged with **20** (1.08 g, 2.09 mmol, 1.0 eq), $PdCl_2dppf$ (92 mg, 126 µmol, 6 mol%), KOAc (617 mg, 6.29 mmol, 3.0 eq) and B_2pin_2 (798 mg, 3.14 mmol, 1.5 eq) under argon. Anhydrous 1,4-dioxane (9 mL) was added and the mixture was heated in an oil bath to 85 °C for 19 h. The black mixture was allowed to cool to ambient temperature and filtered over Celite eluting with EtOAc. The solvent was removed under reduced pressure and the crude was purified by column chromatography on silica gel with 0-5% *n*-heptane in EtOAc. The title compound **12** (843 mg, 1.50 mmol, 72%) was obtained as a colourless solid.

^{1}H NMR (400 MHz, Chloroform-*d*) δ 6.86 (d, *J* = 2.4 Hz, 1H), 6.43 (d, *J* = 2.6 Hz, 1H), 2.35 (s, 3H), 1.33 (s, 12H), 1.31 – 1.17 (m, 6H), 1.11 (d, *J* = 4.6 Hz, 20H), 1.09 (d, *J* = 4.4 Hz, 16H).

^{13}C{^{1}H} NMR (101 MHz, Chloroform-*d*) δ 154.6, 153.8, 130.5 (broad, C-B), 127.3, 119.3, 112.4, 83.5 (2C), 25.0 (4C), 18.2 (6C), 18.1 (6C), 15.1, 13.2 (3C), 12.9 (3C). *Note: The boron bearing carbon was identified using 2D HMBC spectra.*

HRMS (ESI): calcd for $C_{31}H_{60}{}^{11}BO_4{}^{28}Si_2{}^+$ [M+H]$^+$ 563.4118, found: 563.4123.

IR (ATR, v_{max}/cm^{-1}): 2943, 2867, 1572, 1464, 1420, 1355, 1304, 1267, 1147, 1044, 1008, 998, 937, 883, 835, 798.

M$_p$: 56 – 58 °C.

R$_f$: 0.61 (10% EtOAc in *n*-heptane).

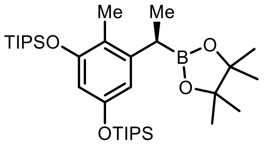

(R)-((4-methyl-5-(1-(4,4,5,5-tetramethyl-1,3,2-dioxaborolan-2-yl)ethyl)-1,3-phenylene)bis(oxy))bis(triisopropylsilane) **23**

(*S*)-stannane **21** (405.8 mg, 0.92 mmol, 1.3 eq) was placed in a heat dried flask under inert atmosphere. The flask was evacuated and backfilled with argon one time. Degassed, dry Et_2O (4.5 mL) was added and the mixture was cooled to -78 °C. *n*-BuLi (398 µL, 1.00 mmol, 1.4 eq, 2.5 M in hexanes) was added dropwise and the mixture was stirred at that temperature for 60 min to yield a clear, yellow solution after the tin-lithium exchange. Arylboronic pinacol ester **12** (400 mg, 0.71 mmol, 1.0 eq), dissolved in dry, degassed Et_2O (1.4 mL), was added to the lithium alkyl dropwise *via* a syringe pump (0.1 mL/min). The resulting mixture was stirred at -78 °C for 3 h, followed by 1 h at -41 °C. Then $MgBr_2$ (1.0 mL, 1.0 mmol, 1.4 eq, 1.0 M in dry MeOH) was added and the cooling bath was removed after 2 min. Upon warming to ambient temperature, a white precipitate appeared. The mixture was stirred at ambient temperature for 2 h. *n*-Heptane was added and the suspension was filtered over Celite eluting with *n*-heptane. The organics were collected, the solvent was removed and *n*-heptane was added to the solids. The filtration procedure was repeated two more times. The crude title compound was obtained as a clear, yellow oil. The crude alkylboronic pinacol ester (*R*)-**23** was used for the next step without further purification.

A small batch was purified on an automated flash system equipped with a silica column and a gradient of 0-10% EtOAc in *n*-heptane to yield the impure title compound as a clear, colourless oil.

^{1}H NMR (400 MHz, Chloroform-*d*) δ 6.43 (d, *J* = 2.3 Hz, 1H), 6.21 (d, *J* = 2.3 Hz, 1H), 2.55 (q, *J* = 7.4 Hz, 1H), 2.12 (s, 3H), 1.32 – 1.20 (m, 6H), 1.25 (d, *J* = 7.3 Hz, 3H), 1.20 (s, 6H), 1.18 (s, 6H), 1.14 – 1.05 (m, 36H).

^{13}C{^{1}H} NMR (101 MHz, Chloroform-*d*) δ 154.4, 153.9, 145.3, 119.2, 112.3, 107.7, 83.2 (2C), 24.8 (2C), 24.7 (2C), 21.8 (broad, C-B), 18.2 (6C), 18.1 (6C), 16.6, 13.2 (3C), 12.8 (3C), 12.1. *Note: The boron bearing carbon was identified using 2D HMBC spectra.*

HRMS (ESI): calcd for $C_{33}H_{63}{}^{11}BO_4{}^{28}Si_2Na^+$ [M+Na]$^+$ 613.4250, found: 613.4258.

IR (ATR, ν_{max}/cm^{-1}): 2948, 2869, 1603, 1581, 1469, 1383, 1328, 1160, 1074, 1019, 944, 888, 858, 795, 687.

$\mathbf{R_f}$: 0.56 (10% EtOAc in *n*-heptane)

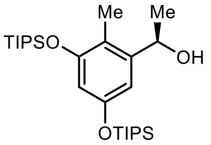

(+)-(R)-1-(2-methyl-3,5-bis((triisopropylsilyl)oxy)phenyl)ethan-1-ol **S10**

An aliquot of the alkylboronic pinacol estser (*R*)-**23** (ca 30 mg) was taken up in a THF:H_2O mixture (1 mL, 1:1) and $NaBO_3$*H_2O (91 mg, 91 µmol, ca 2.5 eq) was added. The suspension was stirred at ambient temperature for 5 h. Then a sat. $NH_4Cl_{(aq)}$ solution was added and the aqueous layer was extracted with EtOAc thrice. The combined organics were dried over Na_2SO_4, filtered and the solvent was removed under reduced pressure. The crude was purified on an automated flash system equipped with a silica column and a gradient of 0-10% EtOAc in *n*-heptane to yield the title compound (+)-(*R*)-**S10** as a clear, colourless oil. No yield was determined.

^{1}**H NMR** (400 MHz, Chloroform-*d*) δ 6.70 (d, *J* = 2.4 Hz, 1H), 6.31 (d, *J* = 2.4 Hz, 1H), 5.08 (q, *J* = 6.4 Hz, 1H), 2.13 (s, 3H), 1.65 (s, 1H), 1.40 (d, *J* = 6.4 Hz, 3H), 1.35 – 1.16 (m, 6H), 1.11 (d, *J* = 6.5 Hz, 18H), 1.09 (d, *J* = 6.5 Hz, 18H).

^{13}**C{^{1}H} NMR** (101 MHz, Chloroform-*d*) δ 154.7, 154.6, 146.0, 117.5, 109.1, 109.0, 67.2, 24.0, 18.2 (6C, 18.1 (6C), 13.2 (3C), 12.8 (3C), 11.2.

HRMS (ESI): calcd for $C_{27}H_{53}O_3{}^{28}Si_2^+$ $[M+H]^+$ 481.3528, found: 481.3529.

IR (ATR, ν_{max}/cm^{-1}): 2944, 2869, 1604, 1580, 1467, 1327, 1165, 1039, 996, 885, 826, 792.

$\mathbf{R_f}$: 0.52 (20% EtOAc in *n*-heptane).

$[\alpha]_D^{20}$: +21.0 (*c* 3.10, $CHCl_3$ /w 0.6% EtOH).

Chiral SFC: ee = 96.5%.

Sample preparation: 0.7/0.2 mL *n*-heptane/EtOH. Method: Trefoil™ CEL1, 1.2 mL/min, 40 °C, 284 nm, ABPR 2000 psi, solvent A: CO_2, solvent B: MeOH, isocratic elution for 10 min with 20% B. t_R = 6.00 min (*R*), 98.23% AUC and t_R = 7.21 min (*S*), 1.77% AUC.

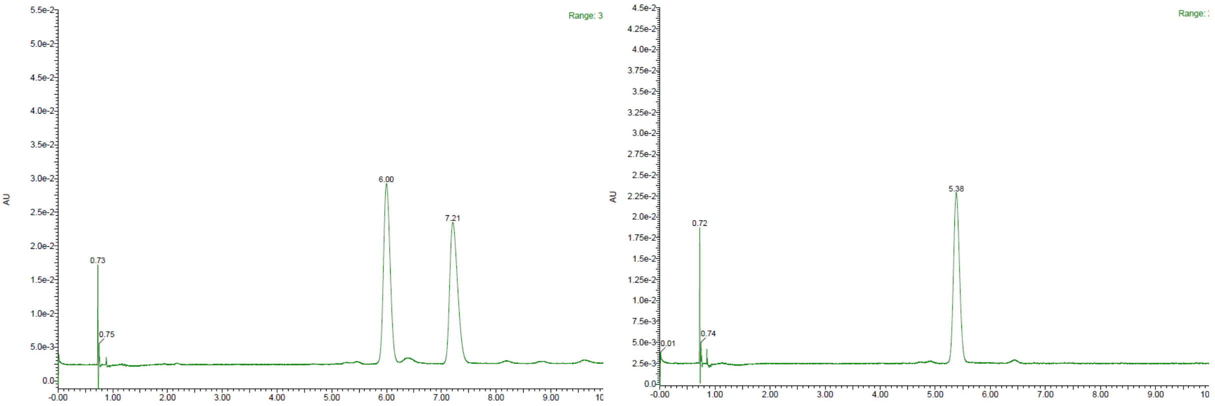

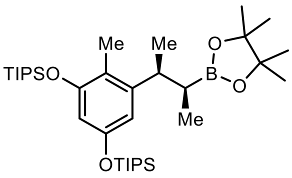

((4-methyl-5-((2S,3S)-3-(4,4,5,5-tetramethyl-1,3,2-dioxaborolan-2-yl)butan-2-yl)-1,3-phenylene)bis(oxy))bis(triisopropylsilane) **25**

(*R*)-stannane **24** (405.8 mg, 0.92 mmol, 1.3 eq) was placed in a heat dried flask under inert atmosphere. The flask was evacuated and backfilled with argon one time. Degassed, dry Et_2O (4.5 mL) was added and the mixture was cooled to -78 °C. *n*-BuLi (398 µL, 1.00 mmol, 1.4 eq, 2.5 M in hexanes) was added dropwise and the mixture was stirred at that temperature for 60 min to yield a clear, yellow solution after the tin-lithium exchange. Crude (*R*)-alkylboronic pinacol ester **23** (1.0 eq), dissolved in dry, degassed Et_2O (1.4 mL),

was added to the lithium alkyl dropwise *via* a syringe pump (0.1 mL/min). The resulting mixture was stirred at -78 °C for 3 h. The mixture was transferred to a -41 °C cooling bath for 60 min. The cooling bath was removed and the mixture was stirred at ambient temperature for 4 h. A white precipitate started to appear upon warming to room temperature. The crude was filtered over Celite eluting with *n*-heptane. The organics were collected, the solvent was removed and *n*-heptane was added to the solids. The suspension was passed over a Celite column once more, eluting with *n*-heptane. The organics were collected, the solvent was removed under reduced pressure to yield the alkylboronic pinacol ester (2*S*,3*S*)-**25** as a crude clear, yellow oil. The crude was used for next step without further purification.

The title compound can be identified by the following analytical data:

^{1}H NMR (400 MHz, Chloroform-*d*) δ 6.36 (d, *J* = 2.3 Hz, 1H), 6.20 (d, *J* = 2.3 Hz, 1H), 3.10 – 2.96 (m, 1H), 2.17 (s, 3H), 1.34 – 1.18 (m, 21H), 1.14 – 1.03 (m, 36H), 0.99 (d, *J* = 7.3 Hz, 3H).

^{13}C{^{1}H} NMR (101 MHz, Chloroform-*d*) δ ~~N/A~~ 154.3, 153.8, 144.9, 118.6, 111.1, 107.4, 82.7 (2C), 36.7, 24.3 (2C), 24.1 (2C), 23.6 (broad, C-B), 19.6, 18.2 (6C), 18.1 (6C), 13.2 (3C), 12.8 (3C), 12.8, 11.7. *Note: The boron bearing carbon was identified using 2D HMBC spectra.*

HRMS (ESI): calcd for $C_{35}H_{68}{}^{11}BO_4{}^{28}Si_2{}^+$ [M+H]$^+$ 619.4744, found: 619.4750.

R$_f$: 0.60 (10% EtOAc in *n*-heptane).

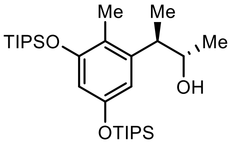

(+)-(2S,3R)-3-(2-methyl-3,5-bis((triisopropylsilyl)oxy)phenyl)butan-2-ol **26**

The crude alkylboronic pinacol ester (2*S*,3*S*)-**25** (1.0 eq) was dissolved in a THF:H_2O mixture (1:1) and $NaBO_3$*H_2O (284 mg, 2.84 mmol, 4.0 eq) was added. The resulting suspension was stirred at ambient temperature for 8 h. Then a sat. $NH_4Cl_{(aq)}$ solution was added and the aqueous layer was extracted with Et_2O thrice. The combined organics were dried over Na_2SO_4, filtered and the solvent was removed under reduced pressure. The crude was purified on an automated flash system equipped with a silica column and a gradient of 0-4% EtOAc in *n*-heptane. The title compound (+)-(2*S*,3*R*)-**26** (217 mg, 426 µmol, 60%) was obtained as a colourless oil.

A racemic sample of (2*S*,3*R*)-**26** and (2*R*,3*S*)-**26** was synthesized by the same procedure using racemic stannane for the homologation reactions. A racemic mixture of stereoisomers (2*S*,3*S*)-**26** and (2*R*,3*R*)-**26** was synthesized accordingly. For the analytical data of the latter *vide infra*.

^{1}H NMR (400 MHz, Chloroform-*d*) δ 6.43 (d, *J* = 2.4 Hz, 1H), 6.30 (d, *J* = 2.3 Hz, 1H), 3.90 – 3.76 (m, 1H), 2.98 (p, *J* = 7.2 Hz, 1H), 2.16 (s, 3H), 1.35 – 1.14 (m, 6H), 1.25 (d, *J* = 6.5 Hz, 3H), 1.14 (d, *J* = 6.9 Hz, 3H), 1.10 (d, *J* = 7.2 Hz, 18H), 1.08 (d, *J* = 7.1 Hz, 28H).

^{13}C{^{1}H} NMR (101 MHz, Chloroform-*d*) δ 155.1, 154.6, 143.8, 120.7, 110.4, 108.9, 72.4, 43.1, 20.4, 18.2 (6C), 18.04 (6C), 18.00, 13.2 (3C), 12.8 (3C), 12.0.

HRMS (ESI): calcd for $C_{29}H_{57}O_3{}^{28}Si_2{}^+$ [M+H]$^+$ 509.3841, found: 509.3840.

IR (ATR, ν_{max}/cm^{-1}): 2948, 2869, 1603, 1581, 1469, 1424, 1331, 1257, 1164, 1000, 944, 888, 791.

R$_f$: 0.37 (10% EtOAc in *n*-heptane).

$[\alpha]_D^{20}$: +20.7 (*c* 5.93, $CHCl_3$ /w 0.6% EtOH).

Chiral SFC: >99.0% ee.

Sample preparation: 0.7/0.2 mL *n*-heptane/EtOH. Method: Trefoil^{TM} CEL1, 1.2 mL/min, 40 °C, 284 nm, ABPR 2000 psi, solvent A: CO_2, solvent B: MeOH, isocratic elution for 10 min with 19% B. t_R = 5.13 min (2*S*,3*R*) and t_R = 5.44 min (2*R*,3*S*).

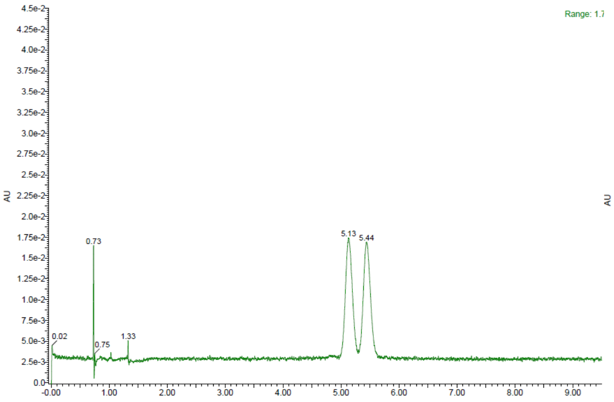

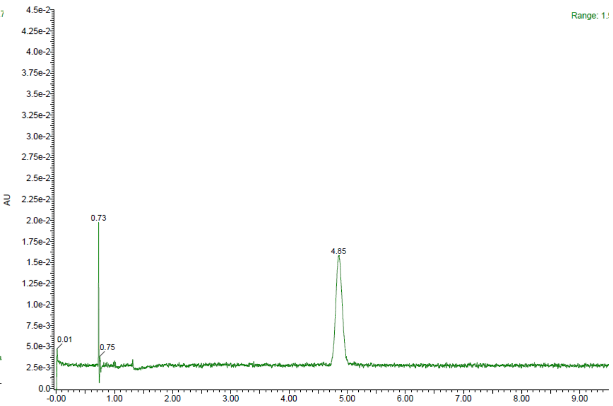

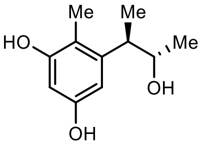

(+)-5-((2R,3S)-3-hydroxybutan-2-yl)-4-methylbenzene-1,3-diol [(+)-Phenol A (**5**)]

Alcohol (+)-(2*S*,3*R*)-**26** (398 mg, 782 µmol, 1.0 eq) was placed in a flask under inert atmosphere. Dry THF (3.0 mL) was added and the clear solution was cooled to 0 °C. TBAF (2.74 mL, 2.74 mmol, 3.5 eq, 1 M in THF) was added and the mixture was stirred at 0 °C for 45 min. Water and a 1 N $NaOH_{(aq)}$ solution were added and the aqueous layer was washed with Et_2O thrice. The aqueous layer was acidified to pH = 3-4 with a 10% citric $acid_{(aq)}$ solution and subsequently extracted with Et_2O thrice. The combined organics were dried over Na_2SO_4, filtered and the solvent was removed under reduced pressure to yield the crude title compound. The crude was purified on an automated flash system equipped with a silica column and a gradient of 25-45% EtOAc in *n*-heptane to yield (+)-phenol A (**5**) (147 mg, 749 µmol, 96%) a white solid.

The analytical data was identical to the previously reported data for synthetic phenol A.[16]

Using the same procedure racemic (2*R**,3*S**)-**26** was converted to racemic (2*R**,3*S**)-**5**, the latter also being called phenol B.

^{1}H NMR (400 MHz, Methanol-*d4*) δ 6.28 (d, *J* = 2.4 Hz, 1H), 6.17 (d, *J* = 2.4 Hz, 1H), 3.86 (p, *J* = 6.3 Hz, 1H), 3.06 (p, *J* = 6.9 Hz, 1H), 2.08 (s, 3H), 1.14 (d, *J* = 7.0 Hz, 3H), 1.12 (d, *J* = 6.3 Hz, 3H).

^{13}C{^{1}H} NMR (101 MHz, Methanol-*d4*) δ 157.0, 156.4, 145.7, 115.3, 105.9, 101.2, 72.0, 43.1, 19.5, 16.4, 10.8.

HRMS (ES): calcd for $C_{11}H_{17}O_3^+$ $[M+H]^+$ 197.1172, found: 197.1173.

IR (ATR, ν_{max}/cm^{-1}): 3276, 2971, 2933, 1596, 1458, 1303, 1267, 1136, 1019, 918, 896, 842, 729.

M_p: 120-122 °C. [Lit. for phenol A[5, 16]: 126-128 °C]

R_f: 0.47 (70% EtOAc in heptane).

$[\alpha]_D^{20}$: +34.5 (*c* 0.67, EtOH). [Lit. for phenol A: –36.8 (*c* 1.07, EtOH)[16] and –36.4 (*c* 0.8, EtOH)[17]]

Chiral SFC: >99.0% ee.

Sample preparation: MeOH. Method: Trefoil™ CEL2, 1.2 mL/min, 40 °C, 284 nm, ABPR 2000 psi, solvent A: CO_2, solvent B: EtOH /w 0.2% TFA, isocratic elution for 10 min with 18% B. t_R = 6.62 min (2*R*,3*S*), and t_R = 6.99 min (2*S*,3*R*).

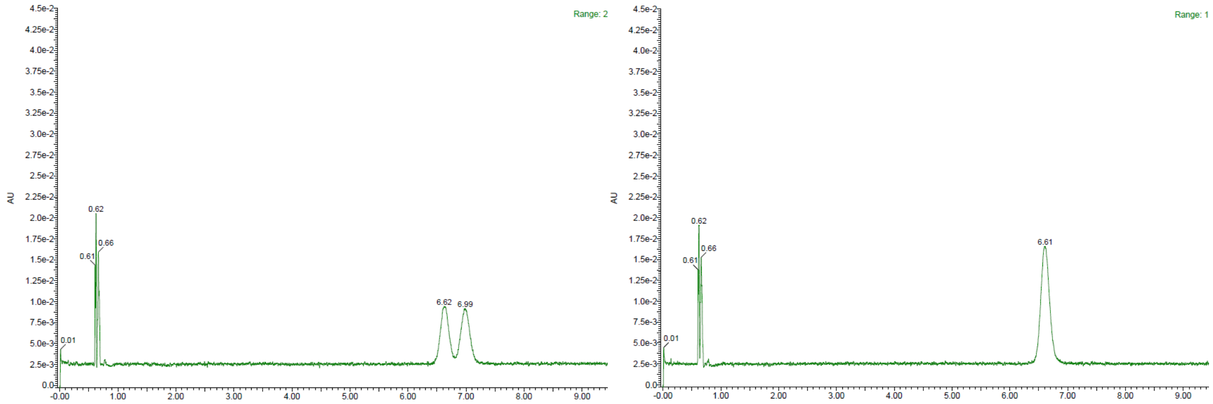

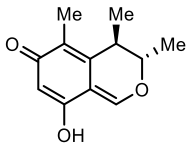

(+)-(3S,4R)-8-hydroxy-3,4,5-trimethyl-3,4-dihydro-6H-isochromen-6-one **27**

(+)-phenol A (**5**) (44.1 mg, 225 µmol, 1.0 eq) was dissolved in EtOAc (1.5 mL). Then triethyl orthoformate (748 µL, 4.49 mmol, 20 eq) and TFA (104 µL, 1.35 mmol, 6.0 eq) were added to yield a yellow solution. The mixture was stirred at ambient temperature for 60 min. Then an aqueous 0.1% TFA solution was added and the aq. layer was extracted with EtOAc thrice. The combined organics were dried over Na_2SO_4, filtered and the solvent was removed under reduced pressure. The crude was dissolved in a small amount of MeOH and water was added. The mixture was lyophilized to yield the mono-TFA adduct of the title compound (+)-(3*S*,4*R*)-**27** (quantitative) as a yellow powder. The crude was used without further purification.

Note: The amount of TFA in the product was determined with qNMR using α,α,α-trifluorotuluene as a standard.

^{1}H NMR (400 MHz, Chloroform-*d*) δ 8.51 (s, 1H), 6.69 (s, 1H), 4.89 (q, J = 6.7 Hz, 1H), 3.03 (q, J = 7.1 Hz, 1H), 2.01 (s, 3H), 1.36 (d, J = 6.7 Hz, 3H), 1.21 (d, J = 7.2 Hz, 3H).

^{13}C{^{1}H} NMR (101 MHz, Chloroform-*d*) δ 180.4, 172.8, 168.5, 139.5, 122.4, 108.6, 101.9, 83.4, 34.4, 19.0, 18.1, 9.8.

HRMS (ESI): calcd for $C_{12}H_{15}O_3^+$ $[M+H]^+$ 207.1016, found: 207.1013.

IR (ATR, ν_{max}/cm^{-1}): 2921, 1618, 1443, 1309, 1190, 1138, 989, 773, 724, 691.

M$_p$: 85 – 91 °C.

R$_f$: 0.32 (50% EtOAc in *n*-heptane /w 0.5% TFA).

$[\alpha]_D^{20}$: +42.4 (*c* 0.60, $CHCl_3$).

Chiral SFC: not obtained.

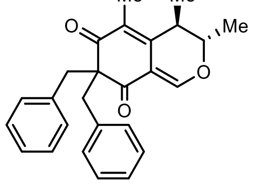

*(–)-(3S,4R)-7,7-dibenzyl-3,4,5-trimethyl-3,4-dihydro-6H-isochromene-6,8(7H)-dione [(–)-(3S,4R)-aspergilone A (**2**)]*

The isochromene (+)-(3*S*,4*R*)-**27** (46.3 mg, 225 µmol, 1.0 eq), Cs_2CO_3 (220 mg, 674 µmol, 3.0 eq) and 18-crown 6 (178 mg, 674 µmol, 3.0 eq) were placed in a vial and dry DMF (1.5 mL) was added to yield a yellow suspension. The mixture was stirred at ambient temperature for 5 min, yielding a brown suspension. Then benzyl iodide (84.4 µL, 674 µmol, 3.0 eq) was added and the mixture was heated to 40 °C using an oil bath. The colour of the liquids reverted back to yellow within 20-30 minutes. One more equivalent of Cs_2CO_3, 18-crown-6 and benzyl iodide were added twice to the reaction mixture, after 90 min and 5 h. After a total of 6 h heating was stopped. EtOAc and a 5% $LiCl_{(aq)}$ solution were added and the layers were separated. The organic layer was washed twice more with the 5% $LiCl_{(aq)}$ solution. Then a 0.1%

$TFA_{(aq)}$ solution was added and the layers were separated. The aqueous layer was extracted twice more with EtOAc. The combined organics were dried over Na_2SO_4, filtered and the solvent was removed under reduced pressure to yield a crude yellow oil. The crude was purified on an automated flash system equipped with a silica column and a gradient of 0-10% EtOAc in *n*-heptane to yield (–)-(3*S*,4*R*)-aspergilone A (**2**) (44.5 mg, 115 µmol, 51% o2s) as a white solid.

Using the same procedure racemic (2*R**,3*S**-phenol A (**5**) was converted to racemic (3*S**,4*R**)-aspergilone A (**2**).

^{1}H NMR (400 MHz, Chloroform-*d*) δ 7.43 (s, 1H), 7.07 – 6.83 (m, 10H), 4.12 (q, *J* = 6.6 Hz, 1H), 3.39 (d, *J* = 12.6 Hz, 1H), 3.31 (d, *J* = 12.4 Hz, 1H), 3.23 (d, *J* = 12.6 Hz, 1H), 3.16 (d, *J* = 12.4 Hz, 1H), 2.29 (q, *J* = 7.1 Hz, 1H), 1.53 (s, 3H), 0.61 (d, *J* = 6.6 Hz, 3H), 0.47 (d, *J* = 7.1 Hz, 3H).

^{13}C{^{1}H} NMR (101 MHz, Chloroform-*d*) δ 200.4, 199.5, 154.6, 144.2, 137.0, 136.6, 130.0 (2C), 129.7 (2C), 128.01 (2C), 127.98 (2C), 126.5, 126.5, 126.1, 111.0, 78.5, 66.3, 46.4, 45.1, 34.7, 18.0, 16.8, 9.6.

HRMS (ESI): calcd for $C_{26}H_{27}O_3^+$ $[M+H]^+$ 387.1955, found: 387.1954.

IR (ATR, ν_{max}/cm^{-1}): 3034, 2963, 2928, 1682, 1609, 1564, 1457, 1373, 1340, 1303, 1172, 1132, 1052, 1016, 989, 976, 897, 838, 750, 702.

M$_p$: 134-138 °C.

R$_f$: 0.36 (20% EtOAc in *n*-heptane).

$[\boldsymbol{\alpha}]_{\boldsymbol{D}}^{\boldsymbol{20}}$: –16.9 (*c* 0.65, $CHCl_3$ /w 0.6% EtOH). [Lit. +18.9 (*c* 0.65, $CHCl_3$)[1]]

Chiral SFC: >99.0% ee.

Sample preparation: 0.9/0.2 mL *n*-heptane/^{i}PrOH. Method: Trefoil™ CEL1, 1.2 mL/min, 40 °C, 312 nm, ABPR 2000 psi, solvent A: CO_2, solvent B: EtOH /w 0.2% TFA, isocratic elution for 10 min with 9% B. t_R = 6.77 min (3*S*,4*R*) and t_R = 7.10 min (3*R*,4*S*).

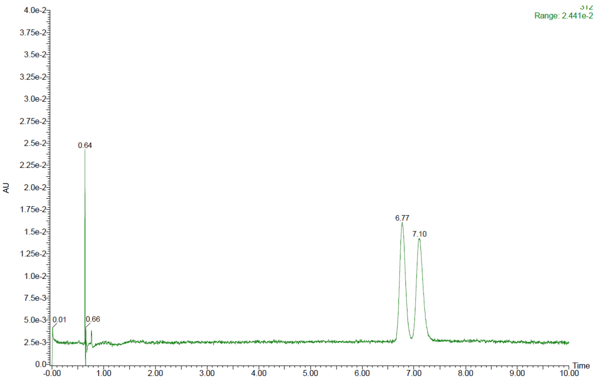

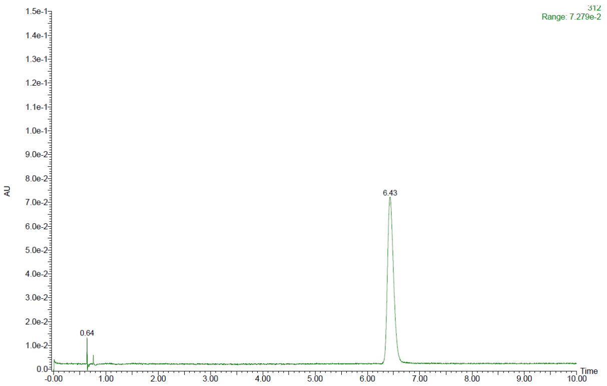

3.5 Synthesis of other compounds and racemic mixtures

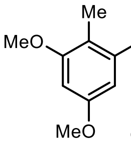

(+)-**S1**

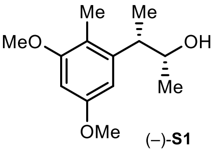

(–)-**S1**

(+)-(2S,3R)-3-(3,5-dimethoxy-2-methylphenyl)butan-2-ol and (–)-(2R,3S)-3-(3,5-dimethoxy-2-methylphenyl)butan-2-ol

To (+)-alcohol **S1** (3 mg) 2 M H_2SO_4 (1 mL) was added and the mixture was stirred for 3 h to induce racemization.[4] The acidic aqueous layer was extracted three times with EtOAc, the organics were combined and the solvent was removed under reduced pressure. The obtained compound was analysed again by chiral SFC.

Sample preparation: MeOH. Method: Trefoil™ CEL2, 1.2 mL/min, 40 °C, 280 nm, ABPR 2000 psi, solvent A: CO_2, solvent B: EtOH ^{i}PrOH:TFA 1:1:0.2%. Gradient: 0 min: 10% B, 7.5 min: 17% B, 8 min 60% B, 9 min 60% B, 9.3 min 10% B, 11 min 10% B.

t_R = 5.45 min (2*S*,3*R*), e.r. = 87.1% and t_R = 5.65 (2*R*,3*S*), e.r. = 12.9%, ee = 74.2%.

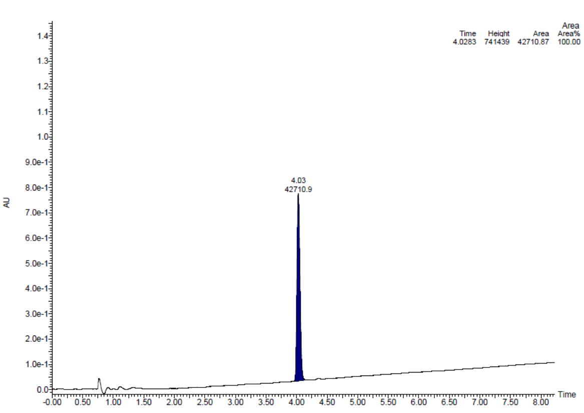

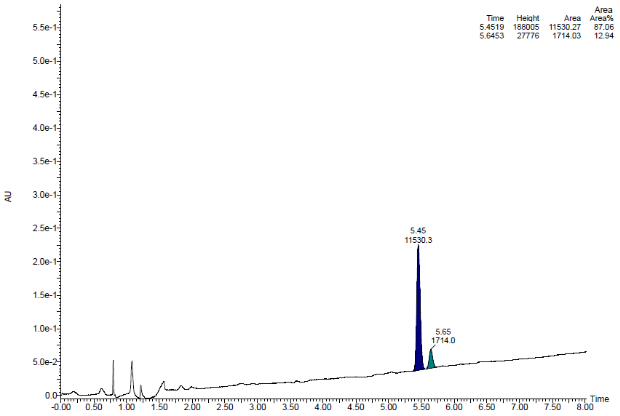

A representative procedure for the simultaneous boronic acid ester hydrolysis and bis-*O*-demethylation is as follows[6, 7]:

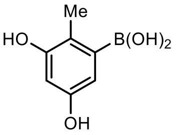

(3,5-dihydroxy-2-methylphenyl)boronic acid ***S4***

N,N-dimethylaniline (123 µL, .0.97 mmol, 9.0 eq) was added to an oven dried microwave vial under inert atmosphere at ambient temperature. Then $AlCl_3$ (129 mg, 0.97 mmol, 9.0 eq) and dry toluene (0.25 mL) were added and the mixture was heated to 80 °C for 30 min to yield a light green solution. Then the Ar-Bpin **S2** (30 mg, 108 µmol, 1.0 eq) and more dry toluene (0.25 mL) were added and the mixture was heated to 160 °C under microwave irradiation for 5 min. A biphasic mixture was obtained. A 1 N HCl solution was added until pH = 1 and the aqueous layer was extracted with EtOAc twice. The organics were combined and the solvent was removed under reduced pressure. The crude yield was determined by qNMR using methyl-3,5-dinitrobenzoate as an internal standard.

A representative procedure for the bis-*O*-demethylation using $S(TMS)_2$ is as follows[10]:

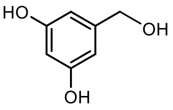

5-(hydroxymethyl)-4-methylbenzene-1,3-diol **S7**

3,5-dimethoxybenzyl alcohol (40 mg, 238 µmol, 1.0 eq) was taken up in dry DMI (0.85 mL) in a heat dried microwave vial under inert atmosphere. NaHMDS (131 µL, 262 µmol, 1.1 eq; 2 M solution in THF) was added and the resulting light brown solution was stirred at ambient temperature for 30 min. The mixture turned dark red during that time. $S(TMS)_2$ (100 µL, 476 µmol, 2.0 eq) was added and the resulting deep blue solution was stirred at ambient temperature for 20 min, before heating to 220 °C under microwave irradiation for 25 min. After cooling, a dark green/brown solution was obtained. The pH was adjusted to 1 with a 1 N HCl solution and the aqueous layer was extracted with EtOAc four times. The organics were combined and the solvent was removed under reduced pressure. The crude yield was determined by qNMR using methyl-3,5-dinitrobenzoate as an internal standard.

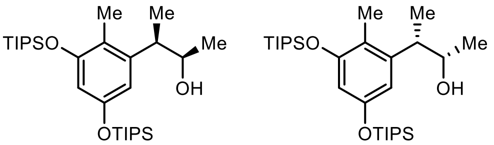

5-((2R,3R)-3-hydroxybutan-2-yl)-4-methylbenzene-1,3-diol and 5-((2S,3S)-3-hydroxybutan-2-yl)-4-methylbenzene-1,3-diol **26**

Following the same procedure as for (+)-**26** the racemic mixture of the stereoisomers (2*S*,3*S*)-**26** and (2*R*,3*R*)-**26** was obtained as a white solid.

^{1}H NMR (400 MHz, Chloroform-*d*) δ 6.34 (d, *J* = 2.3 Hz, 1H), 6.28 (d, *J* = 2.3 Hz, 1H), 3.83 (p, *J* = 6.4 Hz, 1H), 3.03 – 2.93 (h, *J* = 6.8 Hz, 2H), 2.14 (s, 3H), 1.30 – 1.20 (m, 9H), 1.14 – 1.03 (m, 39H).

^{13}C{^{1}H} NMR (101 MHz, Chloroform-*d*) δ 155.0, 154.2, 144.9, 119.3, 111.1, 108.6, 72.3, 42.2, 24.4 (6C), 24.1 (6C), 21.2, 16.1, 13.2 (3C), 12.8 (3C), 12.0.

HRMS (ESI): calcd for $C_{29}H_{57}O_3{}^{28}Si_2^+$ $[M+H]^+$ 509.3841, found: 509.3842.

M$_p$: not obtained.

R$_f$: 0.22 (10% EtOAc in *n*-heptane).

Note: The sample contains ca 1.4 eq of 2,4,6-triisopropylbenzoic acid (TBA).

^{1}H NMR (400 MHz, Chloroform-*d*) δ 6.97 (s, 2H), 2.98 (h, *J* = 6.8 Hz, 2H), 2.82 (h, *J* = 7.0 Hz, 1H), 1.21 (d, *J* = 6.8 Hz, 12H), 1.03 (d, *J* = 8.2 Hz, 6H).

^{13}C NMR (101 MHz, Chloroform-*d*) δ 175.2, 150.6, 145.0 (2C), 129.5, 121.1 (2C), 34.6, 31.7 (2C), 18.2 (2C), 18.0 (4C).

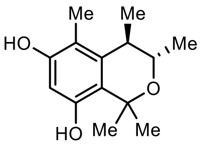

(3S,4R)-1,1,3,4,5-pentamethylisochromane-6,8-diol **S11**

Upon addition of acetone to the (+)-phenol A (**5**) isocromane **S11** was formed. Pure **S11** was obtained after purification on an automated flash system equipped with a silica column and a gradient of 25-45%. The title compound was obtained as a white solid.

^{1}H NMR (400 MHz, Chloroform-*d*) δ 6.12 (s, 1H), 5.12 (bs, 1H), 4.94 (bs, 1H), 3.97 (qd, *J* = 6.5, 3.9 Hz, 1H), 2.71 (qd, *J* = 6.8, 3.9 Hz, 1H), 2.11 (s, 3H), 1.69 (s, 3H), 1.62 (s, 3H), 1.27 (d, *J* = 6.6 Hz, 3H), 1.20 (d, *J* = 6.8 Hz, 3H).

^{13}C{^{1}H} NMR (101 MHz, Chloroform-*d*) δ 152.7, 150.4, 139.6, 121.3, 113.7, 101.8, 73.6, 72.3, 36.8, 29.9, 28.4, 21.8, 20.4, 11.1.

HRMS (ES): calcd for $C_{14}H_{19}O_3^-$ $[M-H]^-$ 235.1340, found: 235.1339.

IR (ATR, ν_{max}/cm^{-1}): 3260, 2974, 2933, 1599, 1428, 1380, 1242, 1171, 1138, 1082, 1056, 1026, 914, 829, 788.

M$_p$: 76-80 °C.

R$_f$: 0.51 (50% EtOAc in heptane).

$[\alpha]_D^{20}$: +38.1 (*c* 1.34, $CHCl_3$).

Chiral SFC: >99.0% ee.

Sample preparation: *n*-heptane:^{i}PrOH:MeOH 1:1:1. Method: Trefoil™ CEL2, 1.2 mL/min, 40 °C, 284 nm, ABPR 2000 psi, solvent A: CO_2, solvent B: EtOH /w 0.2% TFA, isocratic elution for 10 min with 20% B. t_R = 4.33 min (3*S*,4*R*).

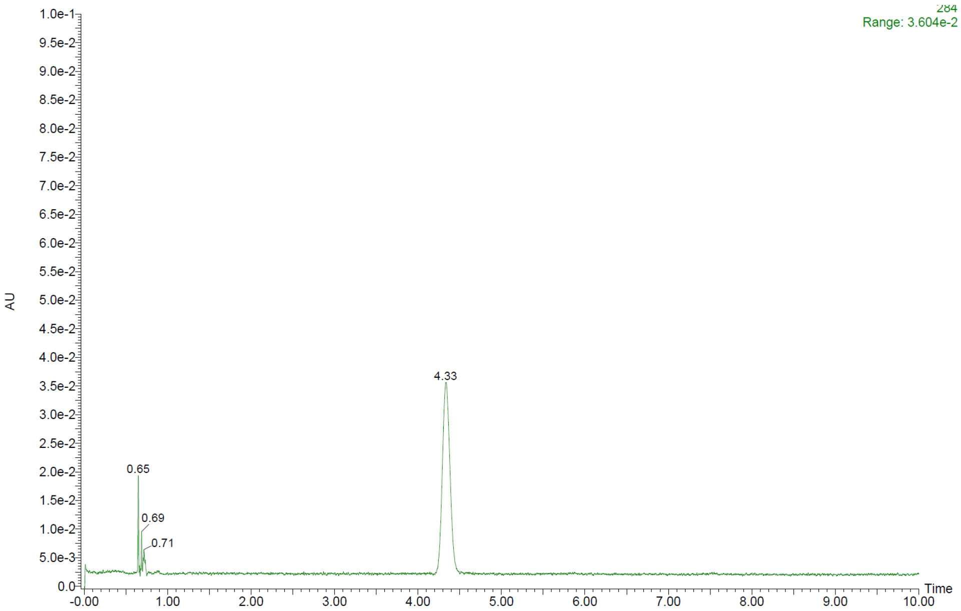

To convert the isochromane back to *ent*-phenol A, the former was taken up in a 2:2:1 mixture of EtOAc:H_2O:TFA (1 mL per 0.1 mmol substrate) and the mixture was stirred at ambient temperature until TLC indicated full consumption of the isochromane. Water and EtOAc were added and the layers were separated. The aq layer was extracted with EtOAc twice more. The combined organics were dried over Na_2SO_4, filtered and the solvent was removed under reduced pressure. The crude was purified on an automated flash system equipped with a silica column and a gradient of 25-45% EtOAc in *n*-heptane to yield pure *ent*-Phenol A.

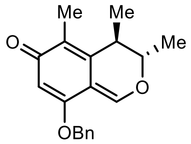

(3S,4R)-8-(benzyloxy)-3,4,5-trimethyl-3,4-dihydro-6H-isochromen-6-one **S12**

From the reaction mixture of (–)-aspergilone (**2**), the title compound (5.8 mg, 19.6 µmol, 9%) was obtained as a white solid.

^{1}H NMR (400 MHz, Chloroform-*d*) δ 7.65 (s, 1H), 7.47 – 7.30 (m, 5H), 5.89 (s, 1H), 4.97 (s, 2H), 4.55 (q, *J* = 6.6 Hz, 1H), 2.88 (q, *J* = 7.1 Hz, 1H), 1.96 (s, 3H), 1.29 (d, *J* = 6.8 Hz, 3H), 1.20 (d, *J* = 7.2 Hz, 3H).

^{13}C{^{1}H} NMR (101 MHz, Chloroform-*d*) δ 187.3, 163.2, 152.6, 135.9, 135.8, 128.8 (2C), 128.5, 127.9 (2C), 127.3, 107.1, 102.4, 79.6, 70.1, 35.0, 18.7, 18.4, 10.0.

HRMS (ES): calcd for $C_{19}H_{21}O_3^+$ $[M+H]^+$ 297.1485 found: 297.1486.

R$_f$: 0.44 (70% EtOAc in *n*-heptane).

4. NMR spectra

^{1}H (400 MHz) and ^{13}C{^{1}H} (101 MHz) NMR spectra of compound **S8** in Chloroform-*d*.[13]

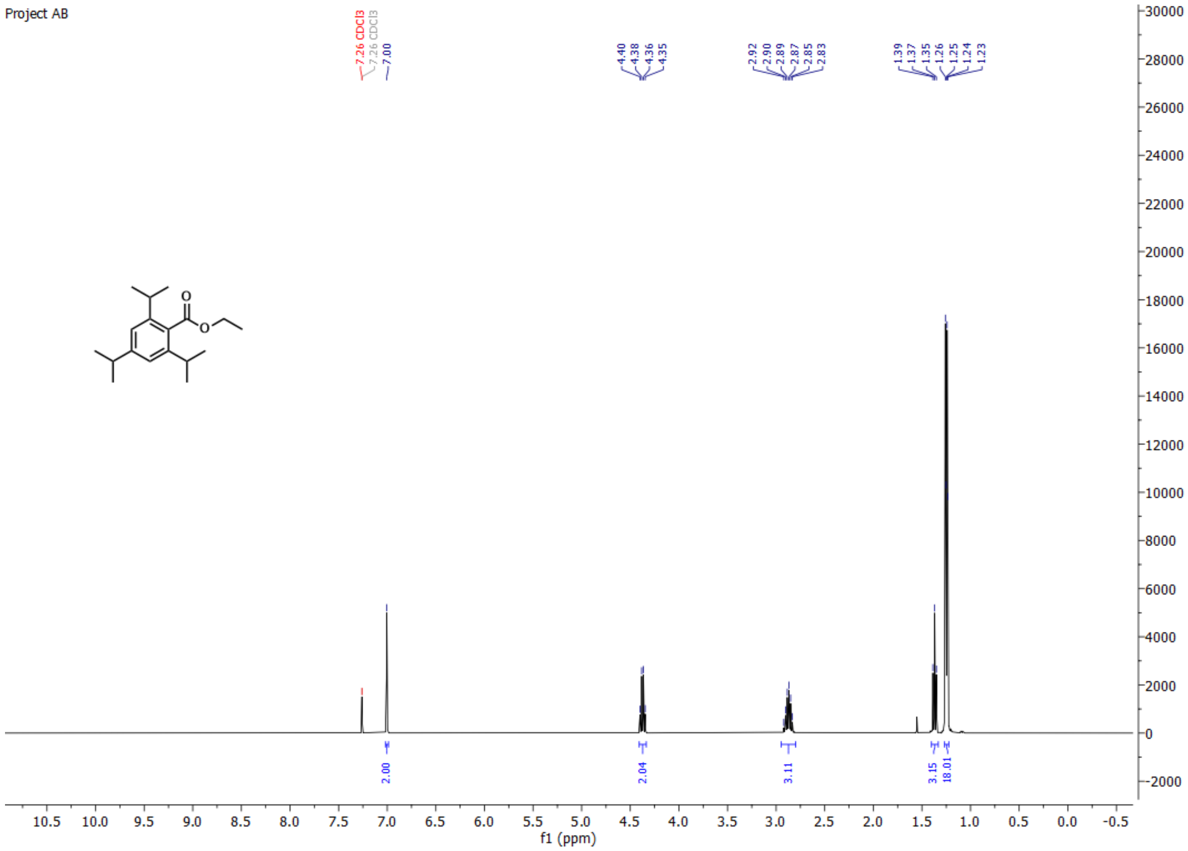

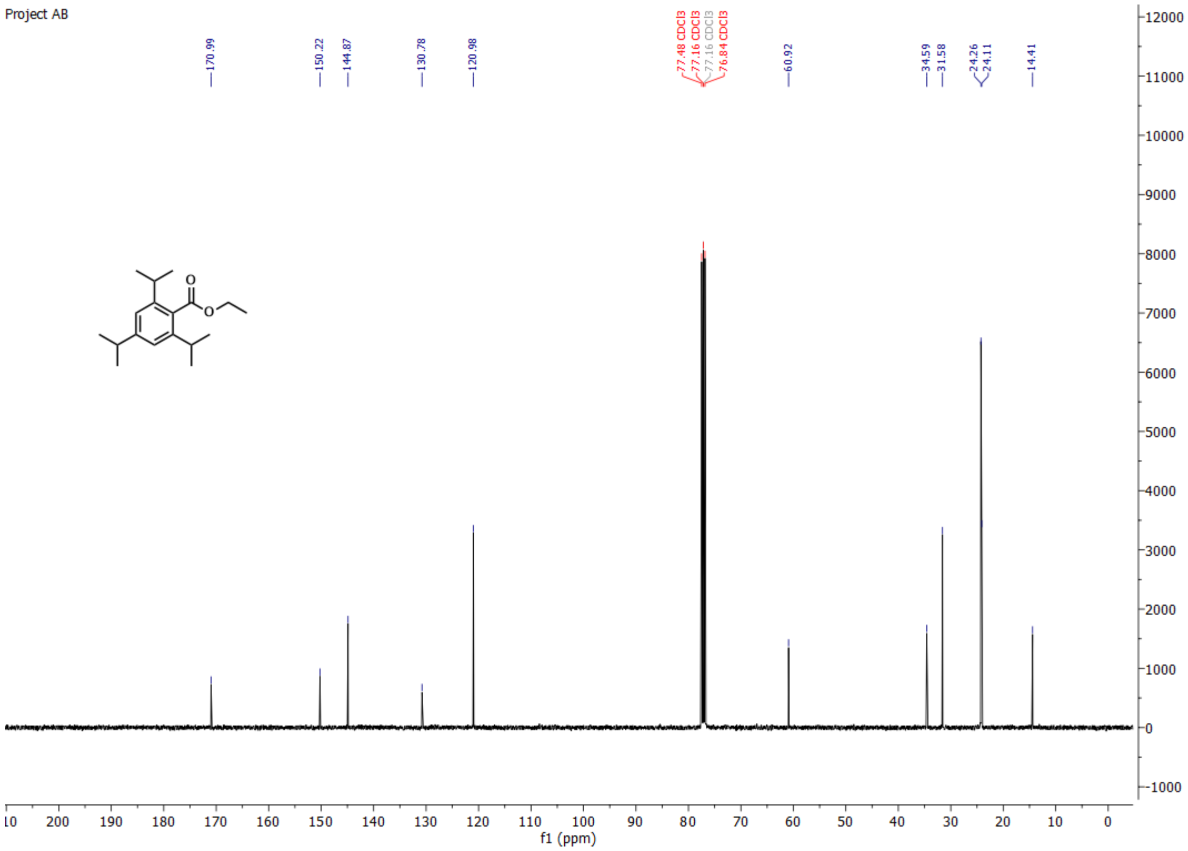

^{1}H (400 MHz) and $^{13}C\{^{1}H\}$ (101 MHz) NMR spectra of compound **21** in Chloroform-*d*.[13]

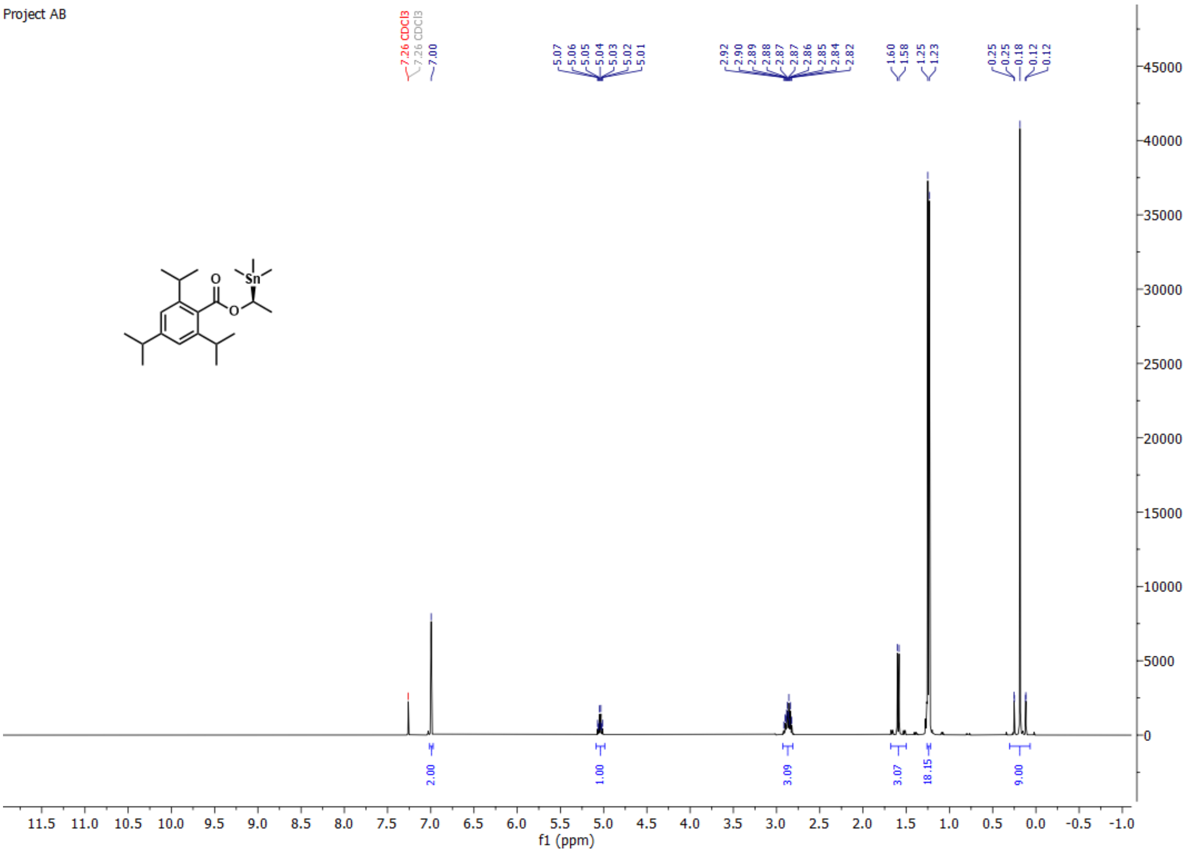

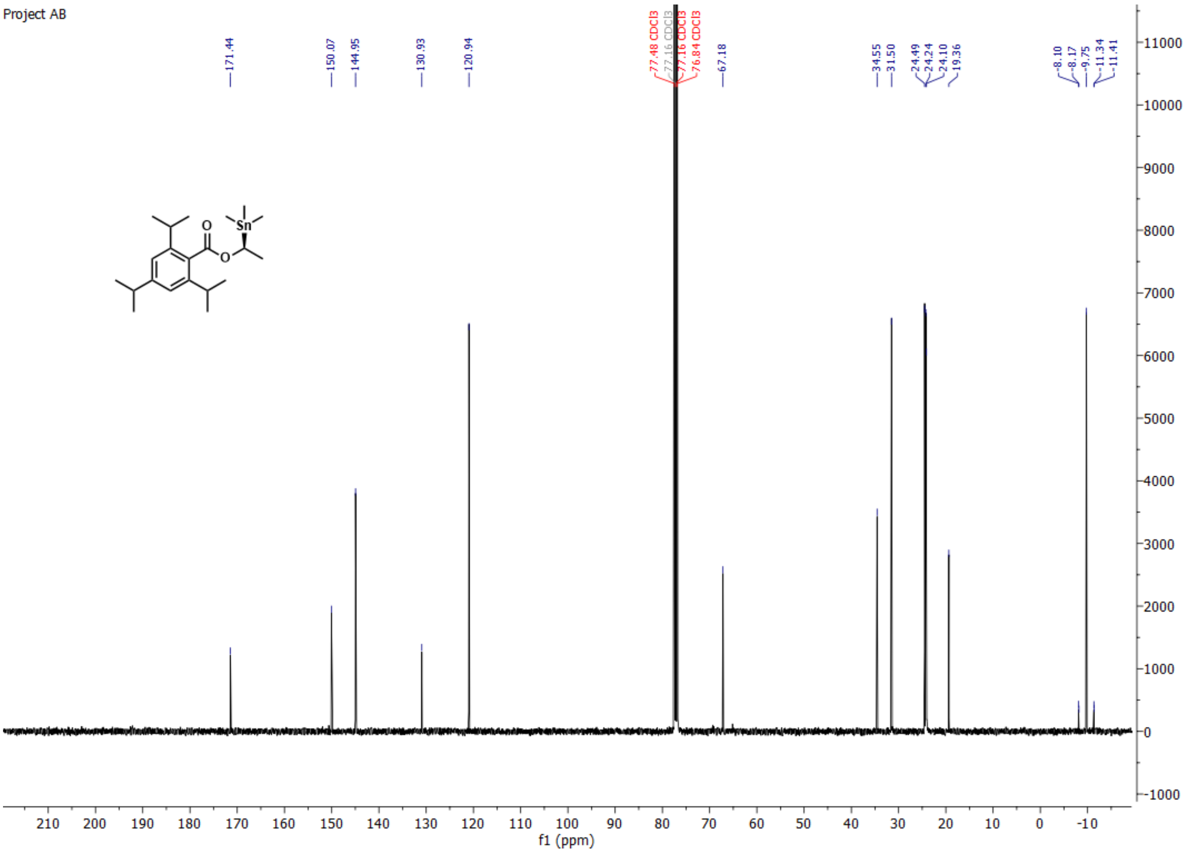

^{1}H (400 MHz) and ^{13}C{^{1}H} (101 MHz) NMR spectra of compound **17** in Chloroform-*d*.

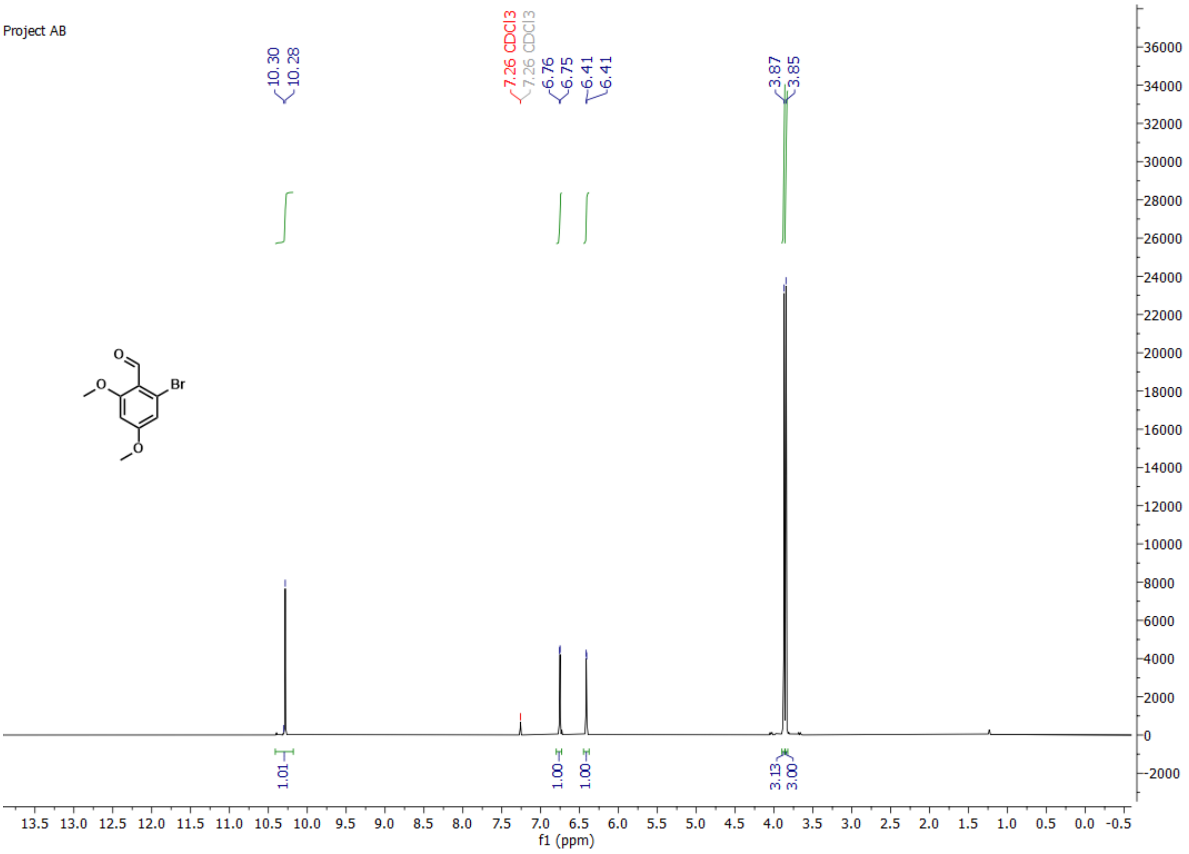

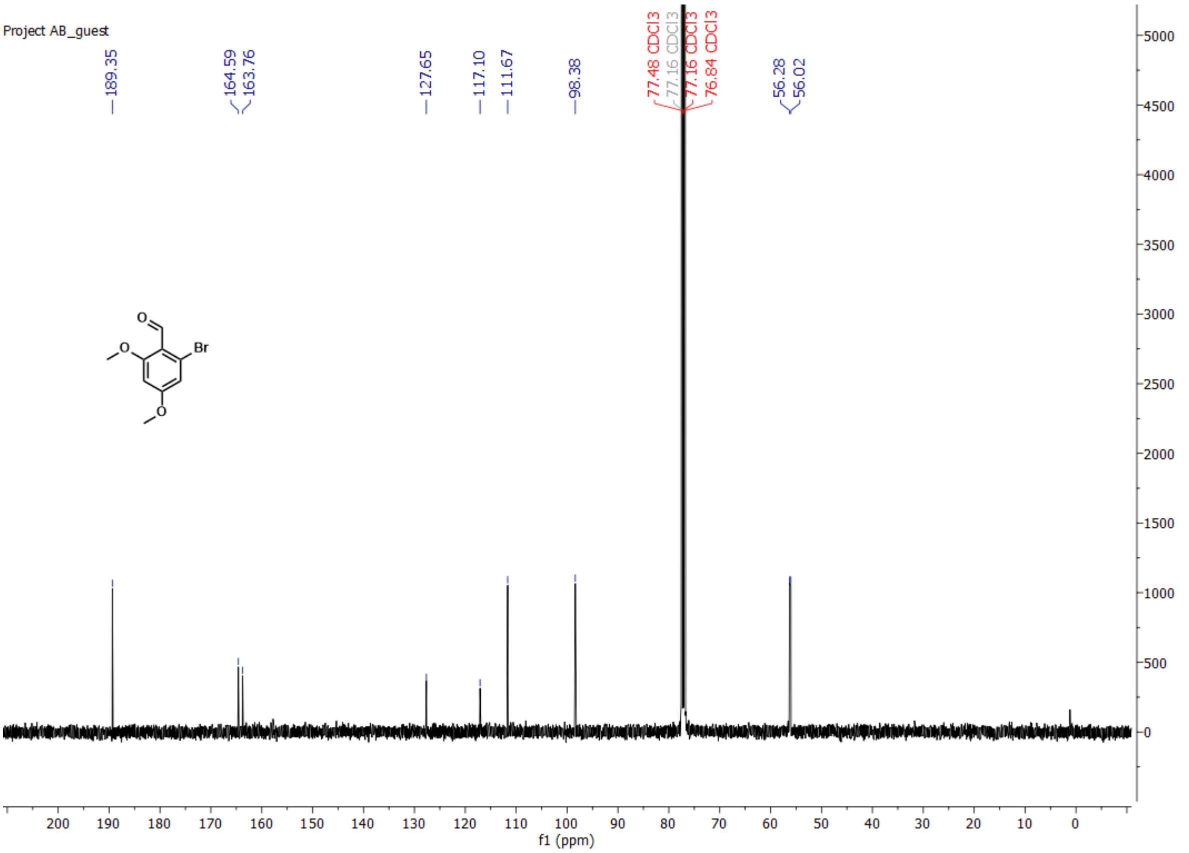

^{1}H (400 MHz) and $^{13}C\{^{1}H\}$ (101 MHz) NMR spectra of compound **18** in Chloroform-*d*.

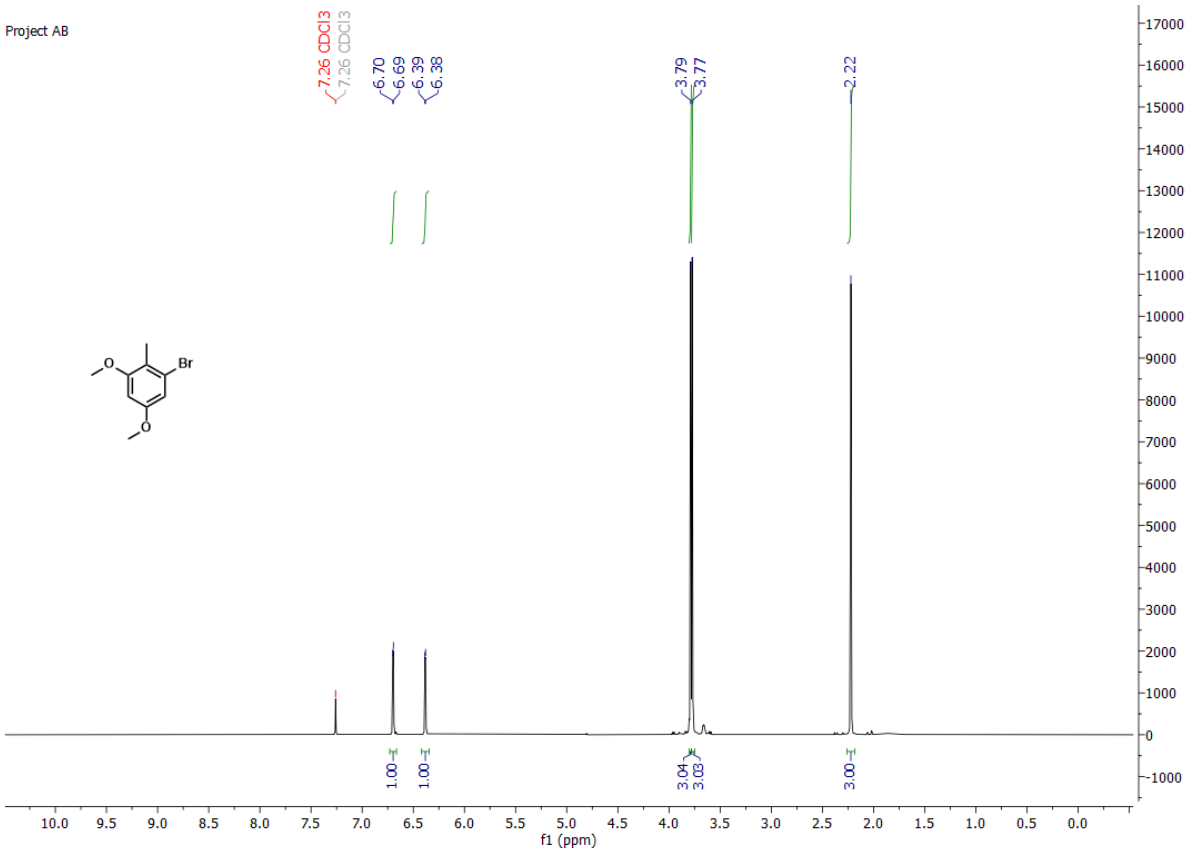

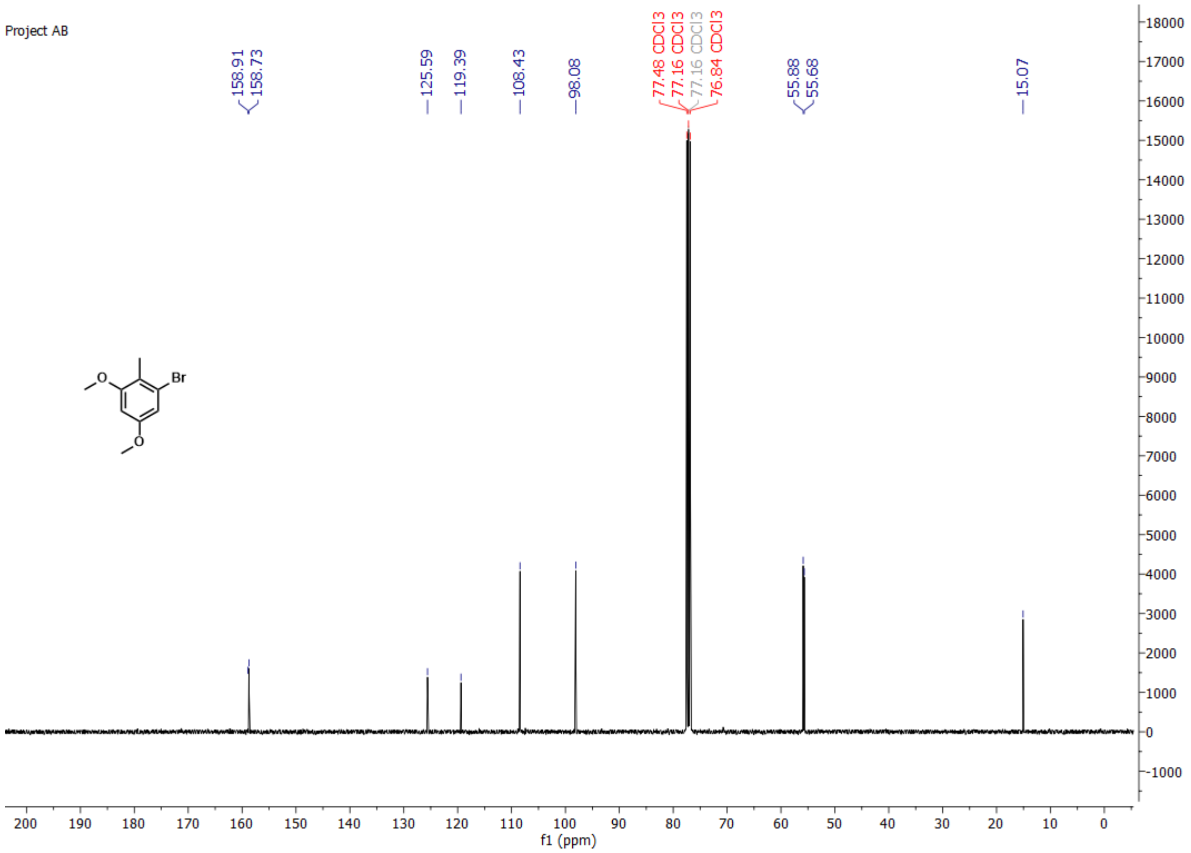

^{1}H (400 MHz) and $^{13}C\{^{1}H\}$ (101 MHz) NMR spectra of compound **19** in Acetone-*d6*.

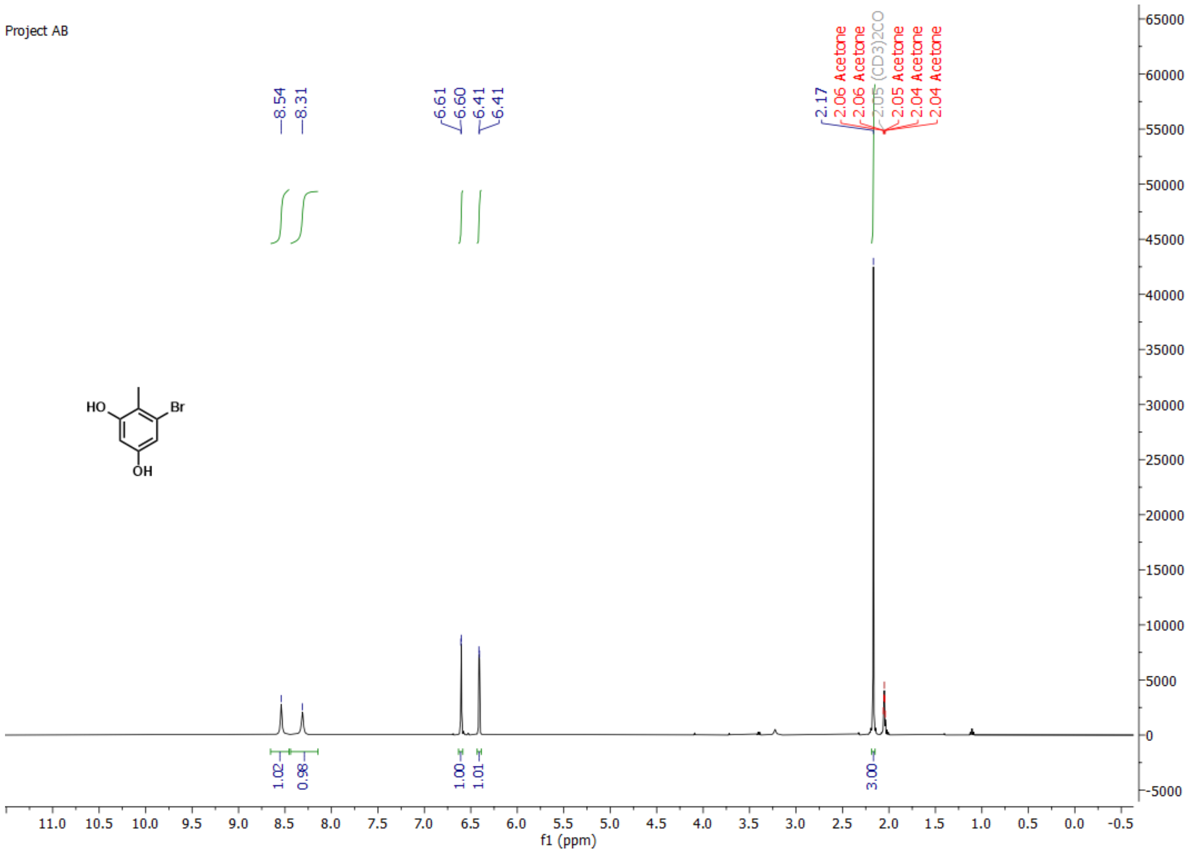

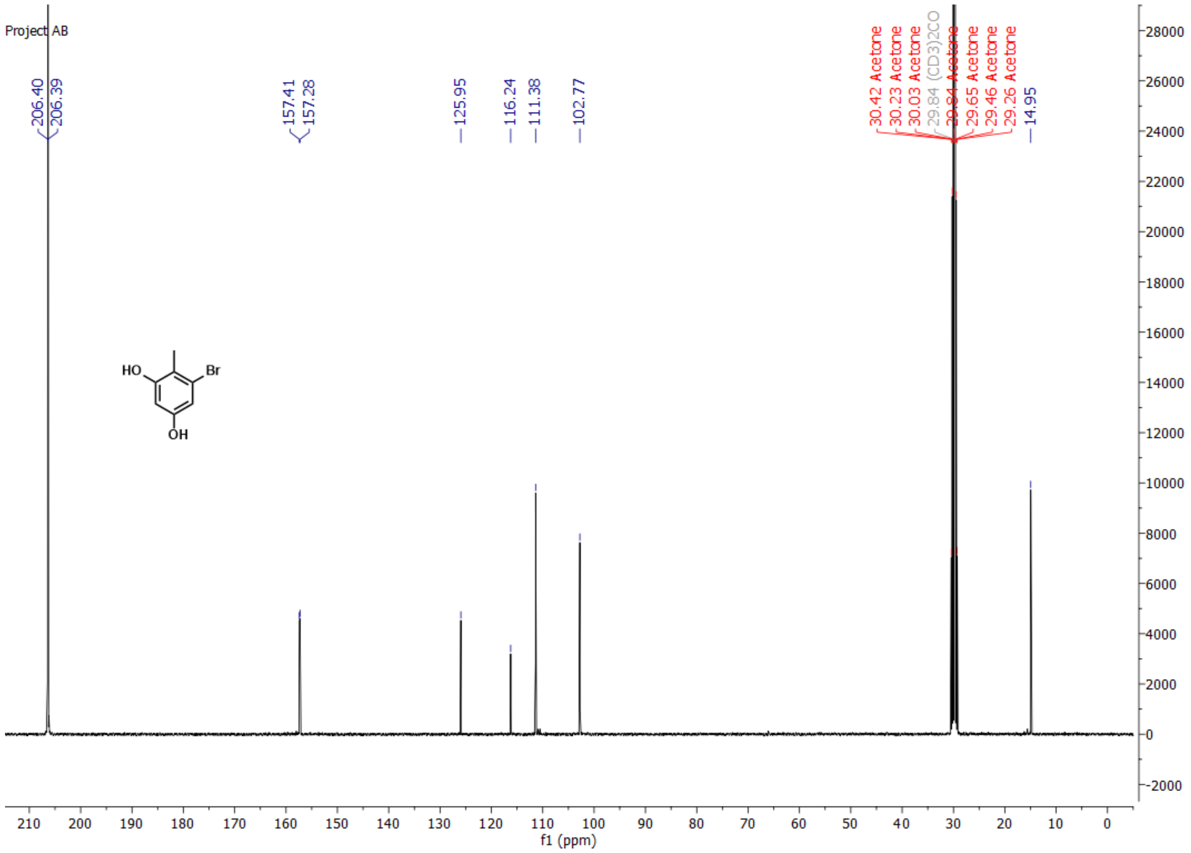

^{1}H (400 MHz) and ^{13}C{^{1}H} (101 MHz) NMR spectra of compound **20** in Chloroform-*d*.

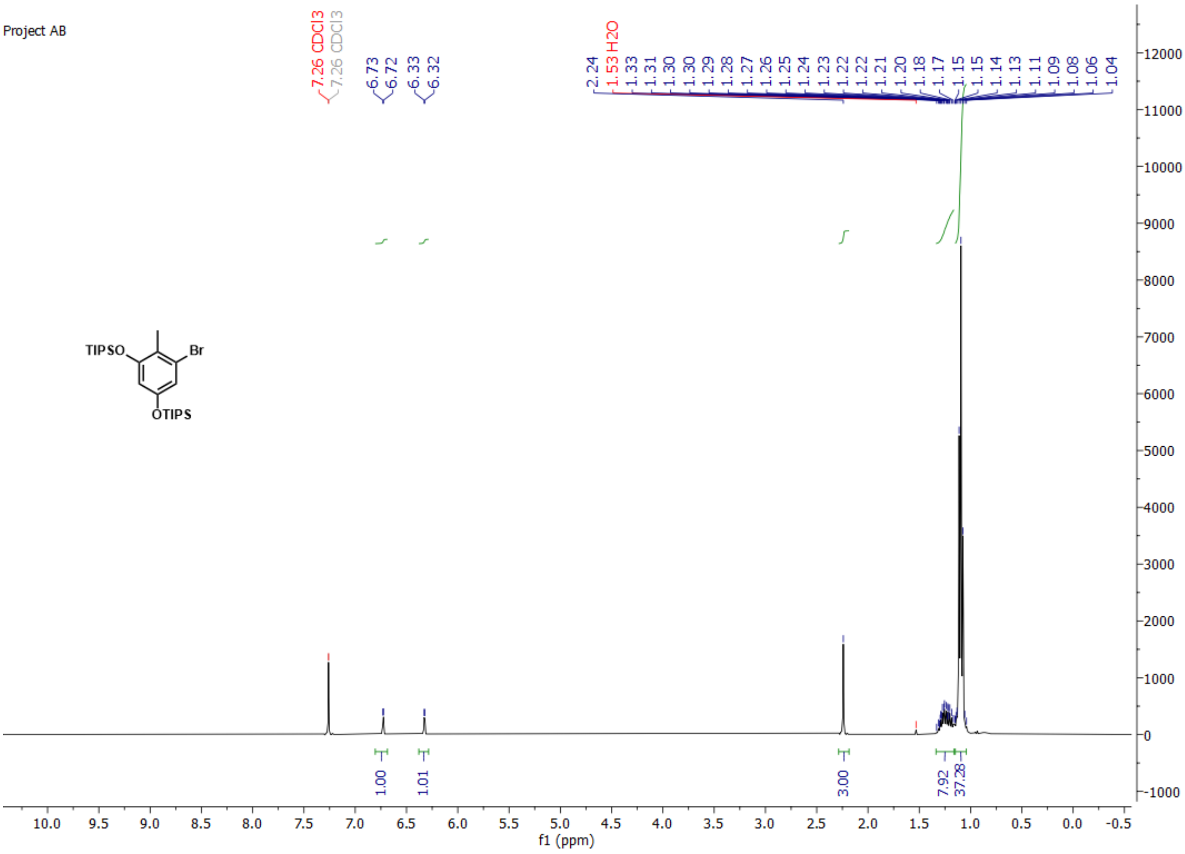

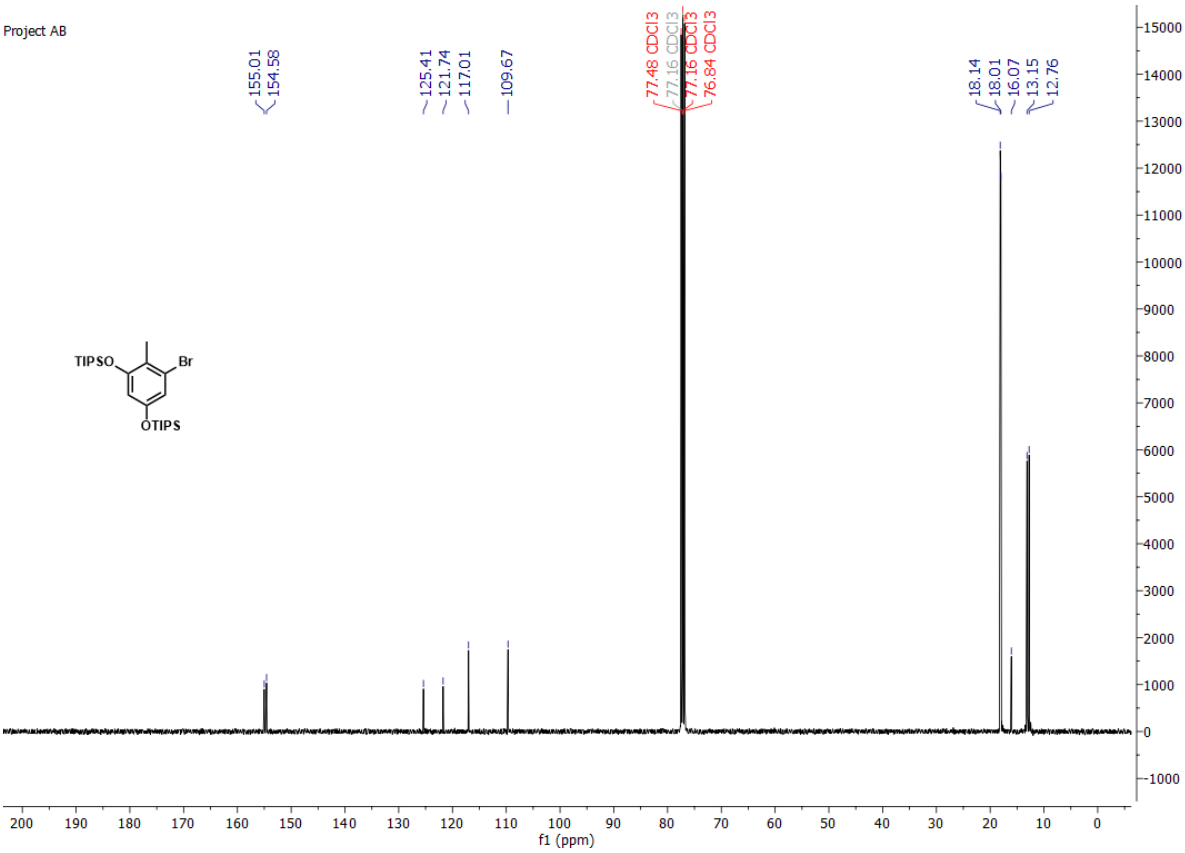

^{1}H (400 MHz) and ^{13}C{^{1}H} (101 MHz) NMR spectra of compound **12** in Chloroform-*d*.

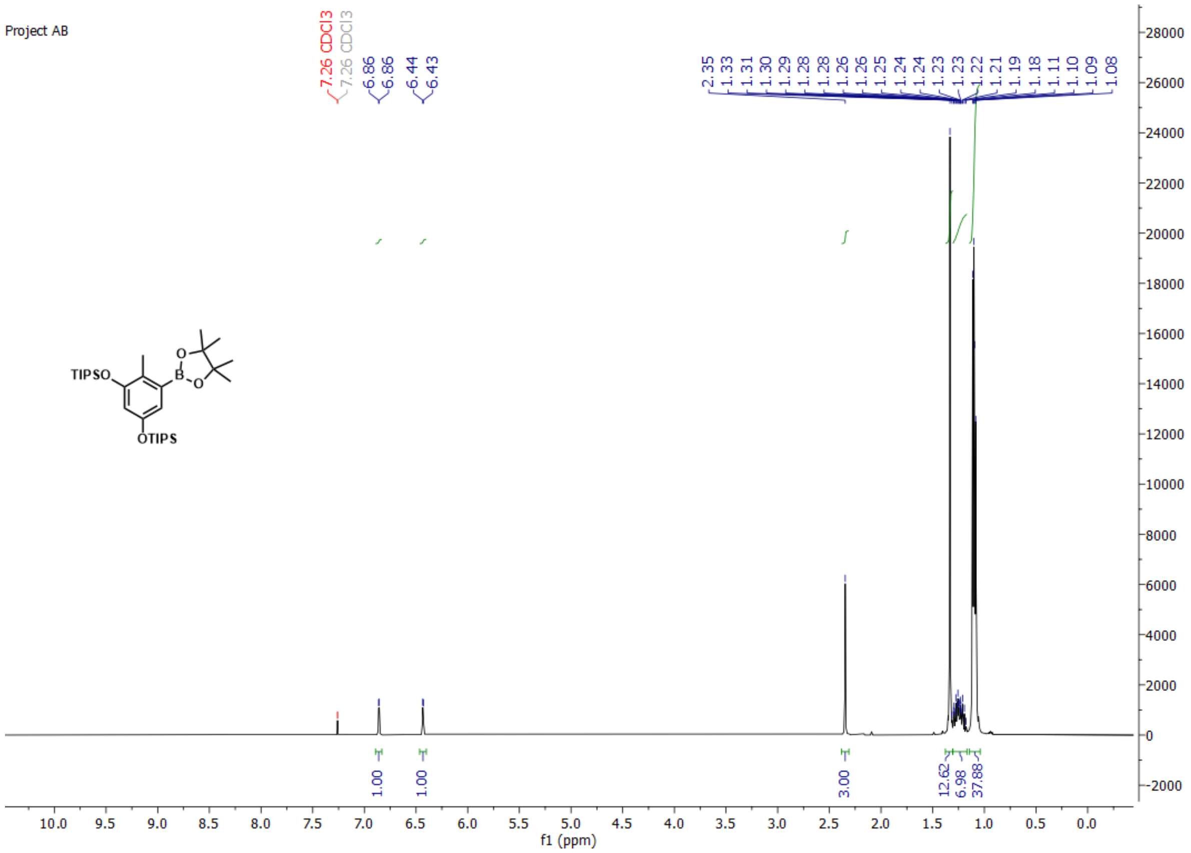

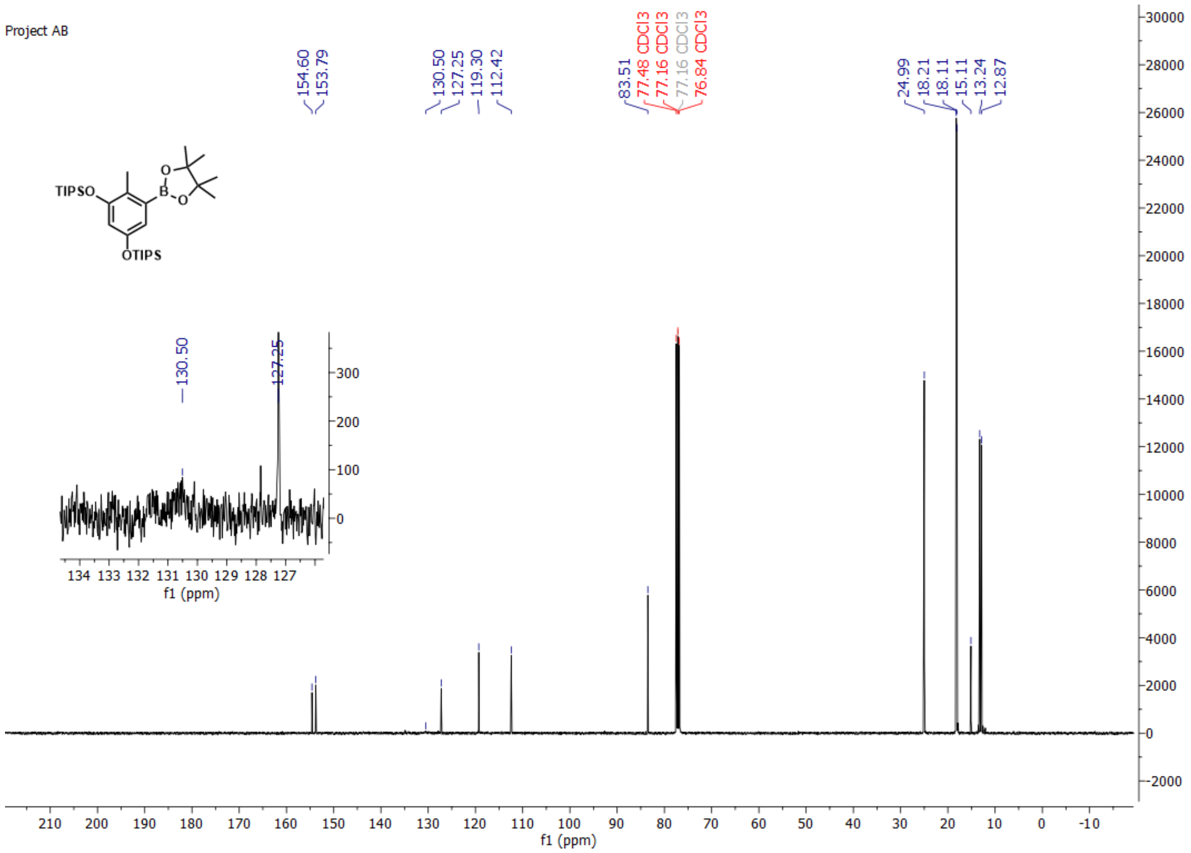

^{1}H (400 MHz) and ^{13}C{^{1}H} (101 MHz) NMR spectra of compound **23** in Chloroform-*d*.

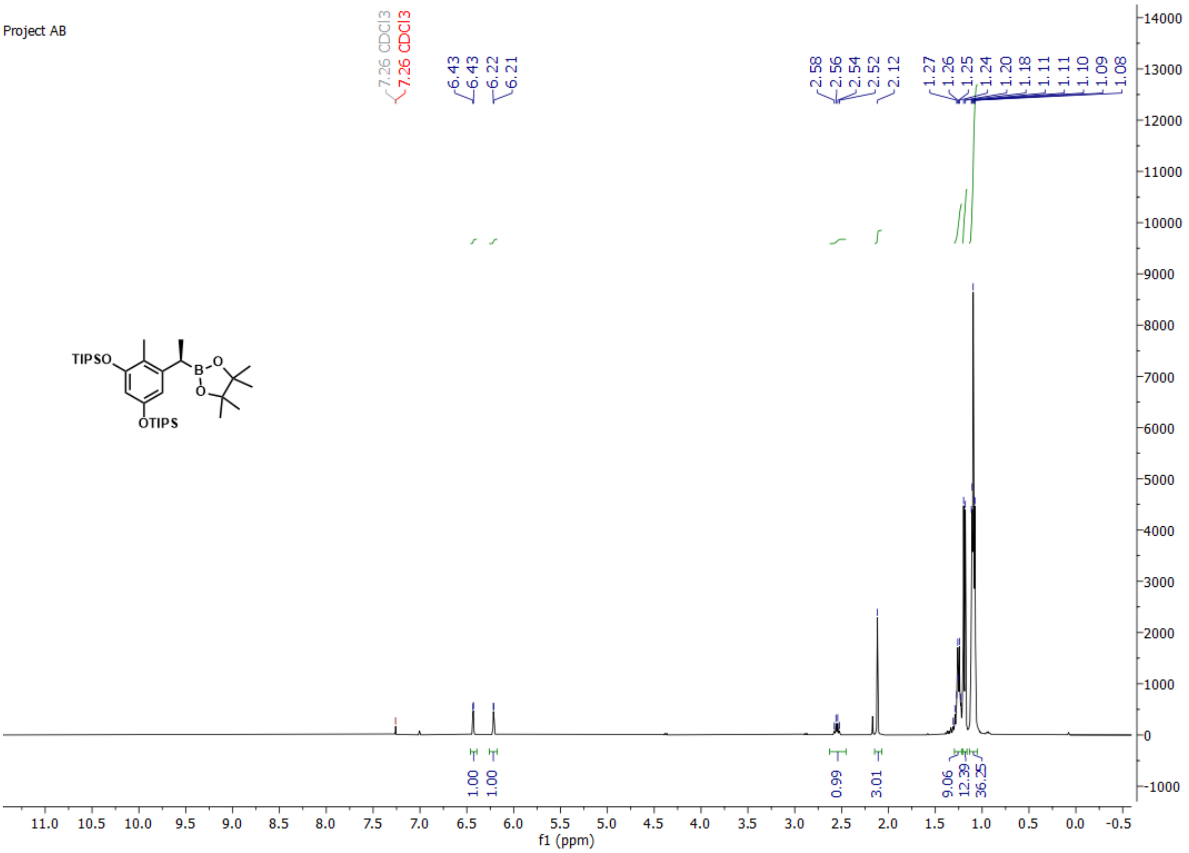

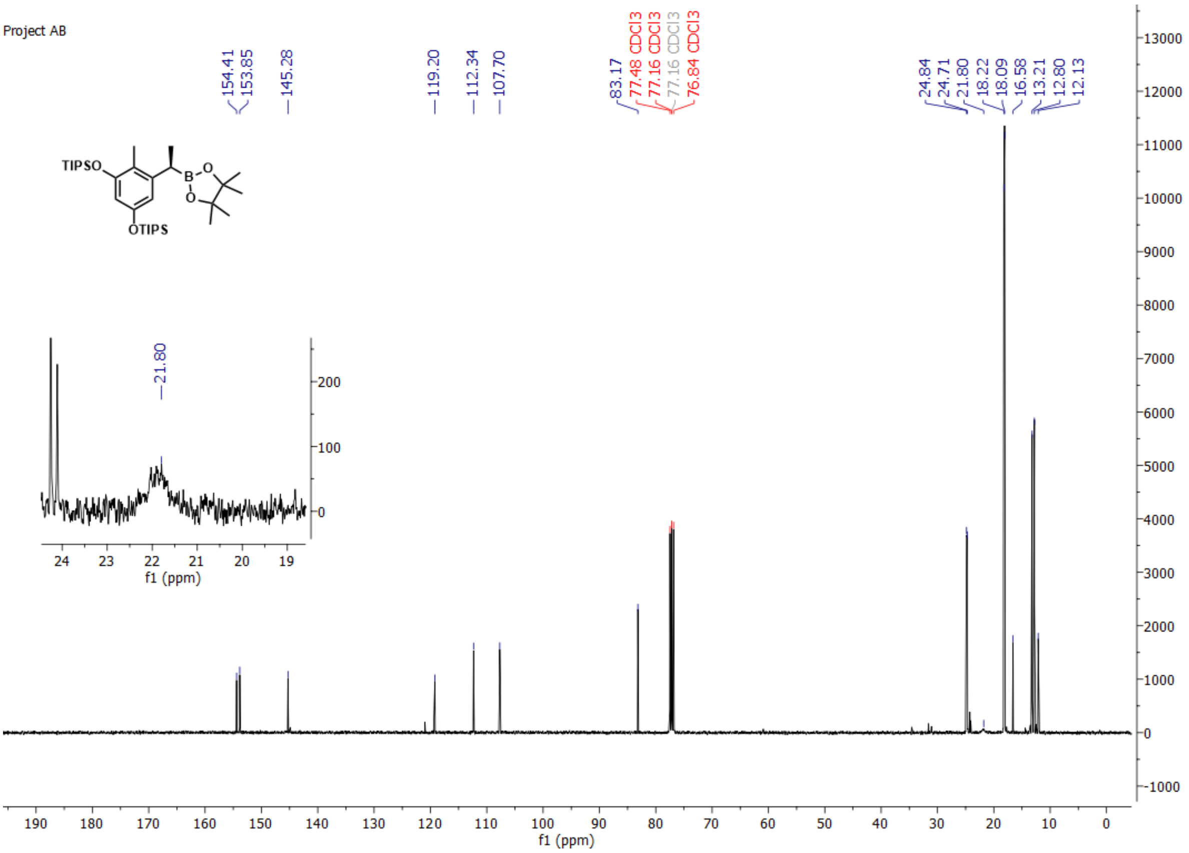

^{1}H (400 MHz) and $^{13}C\{^{1}H\}$ (101 MHz) NMR spectra of compound **S10** in Chloroform-*d*.

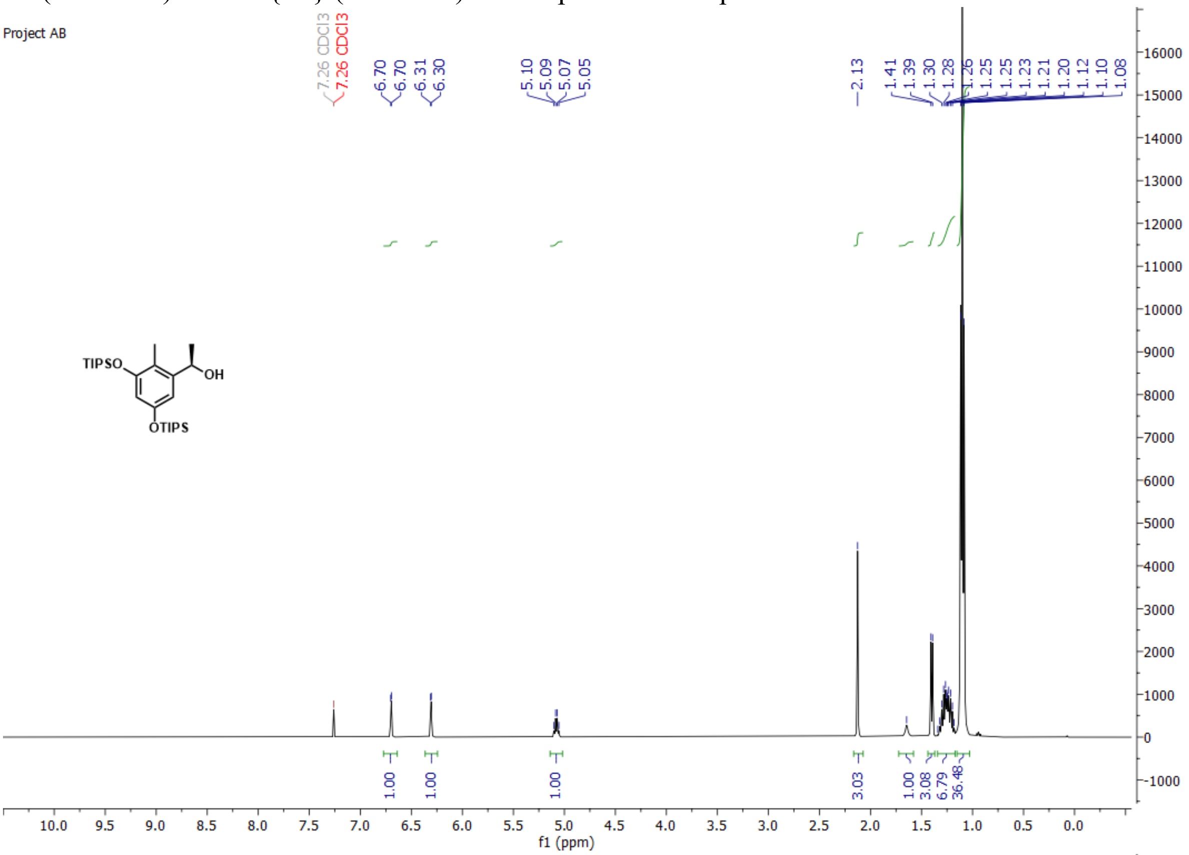

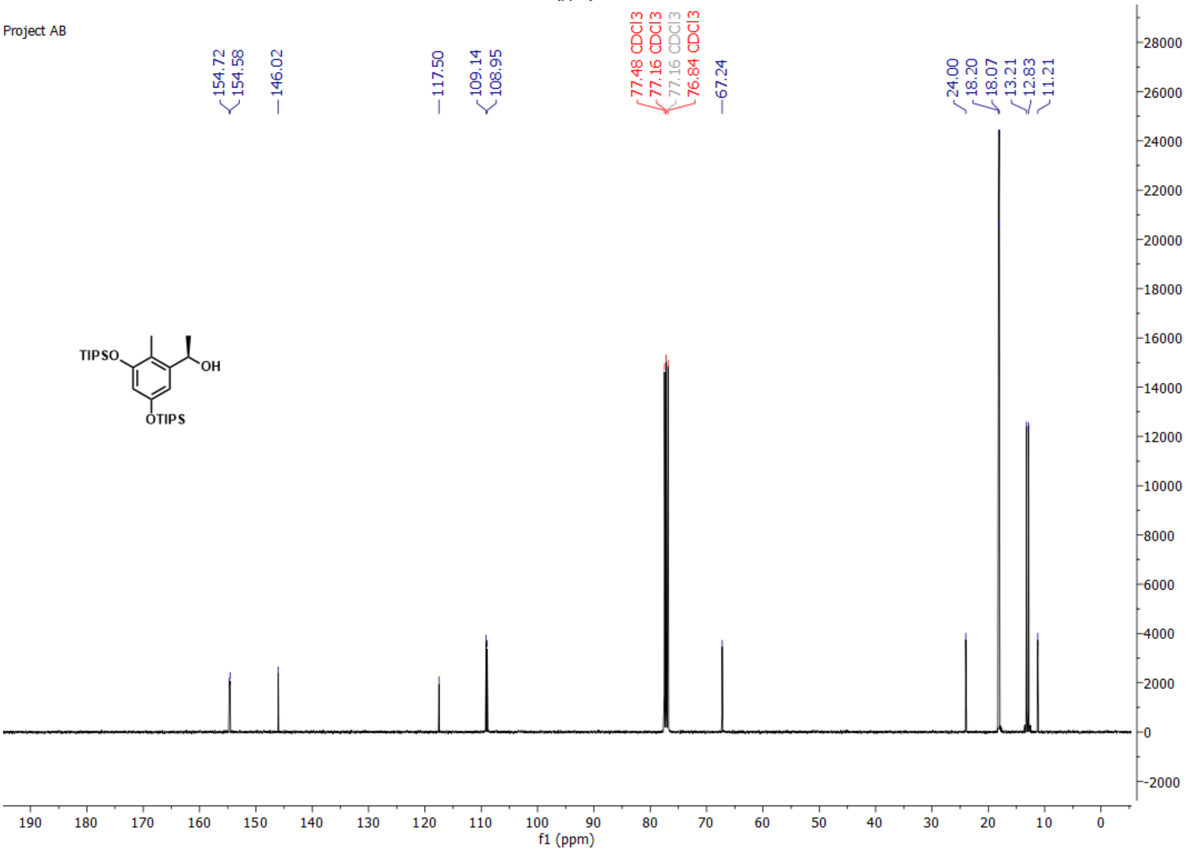

^{1}H (400 MHz) and $^{13}C\{^{1}H\}$ (101 MHz) NMR spectra of compound **25** in Chloroform-*d*.

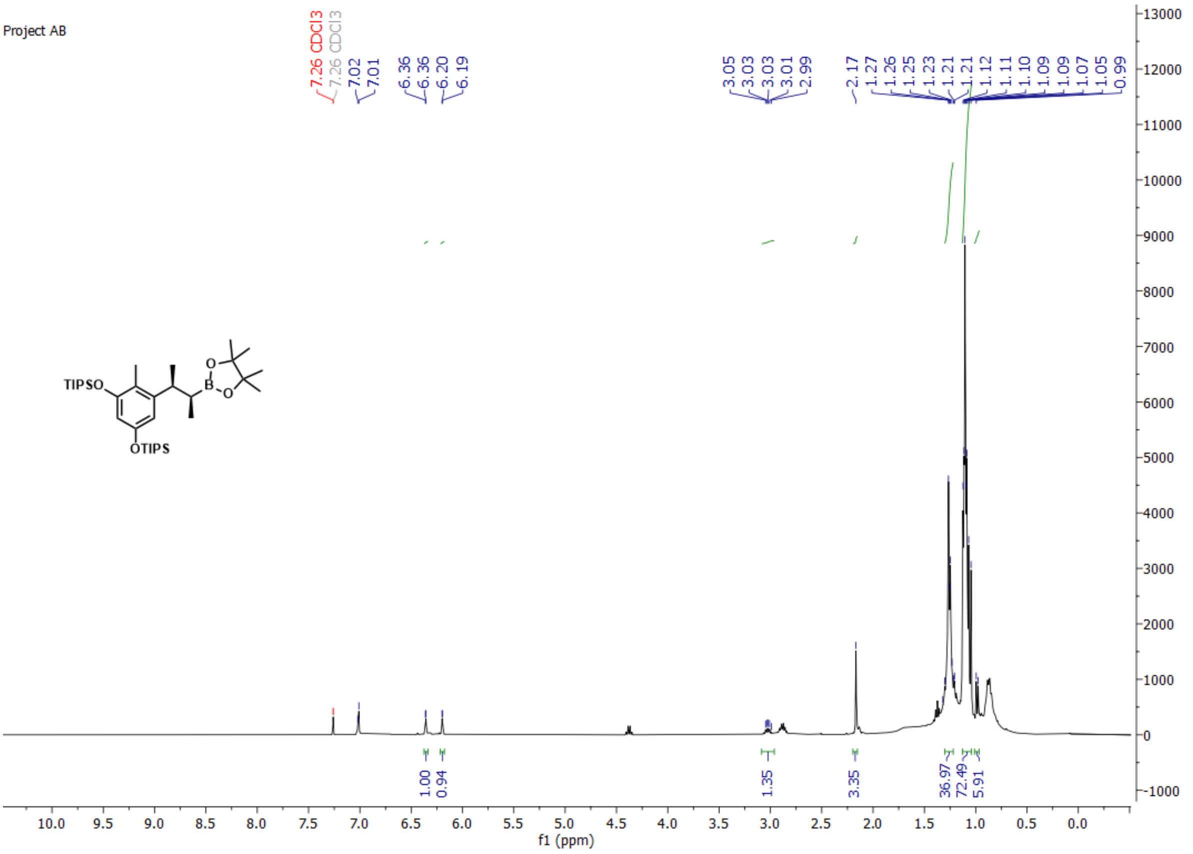

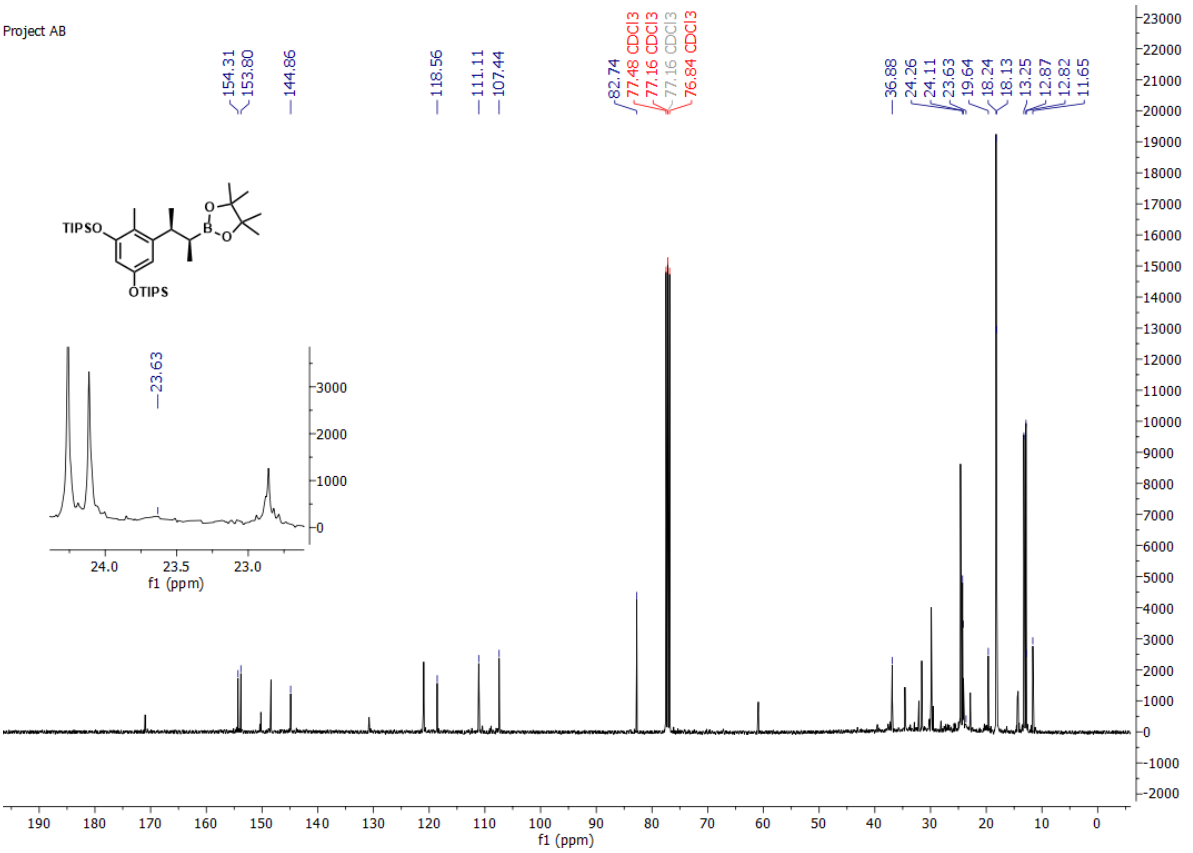

^{1}H (400 MHz) and ^{13}C{^{1}H} (101 MHz) NMR spectra of compound **26** in Chloroform-*d*.

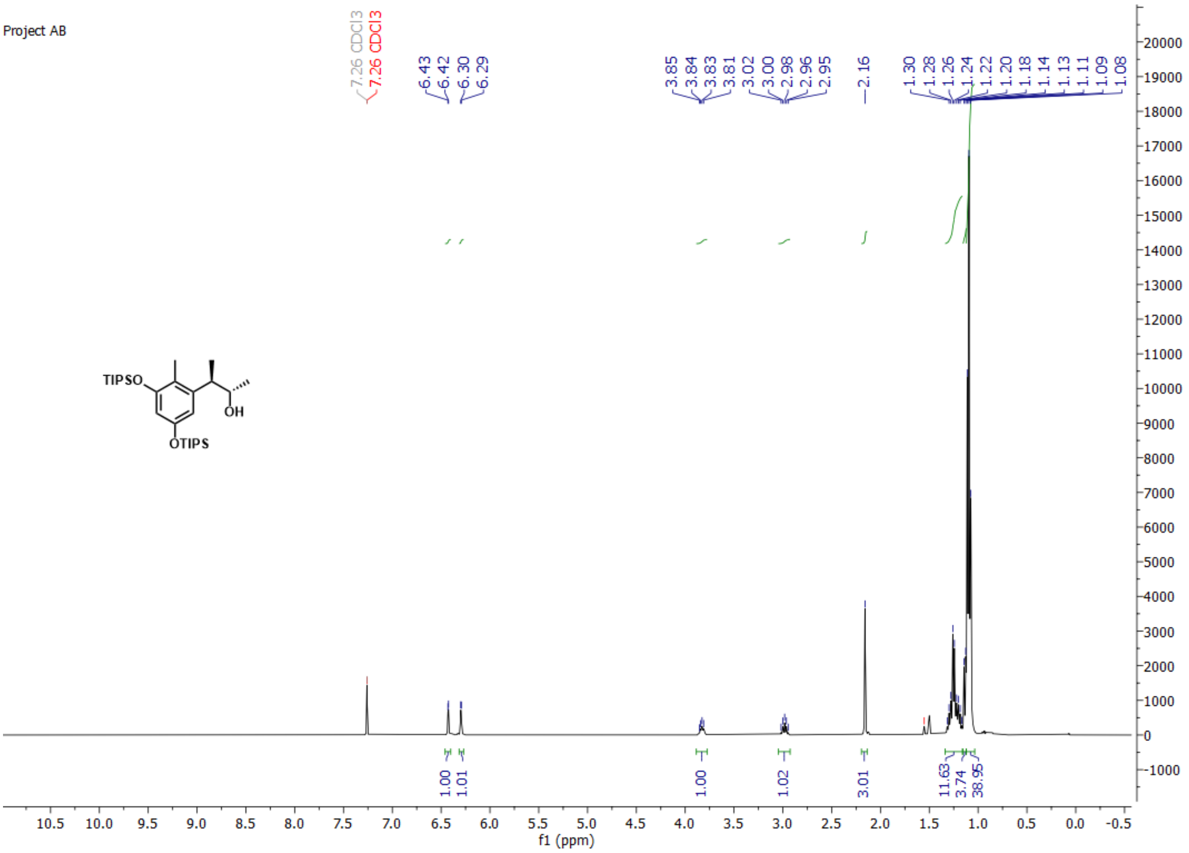

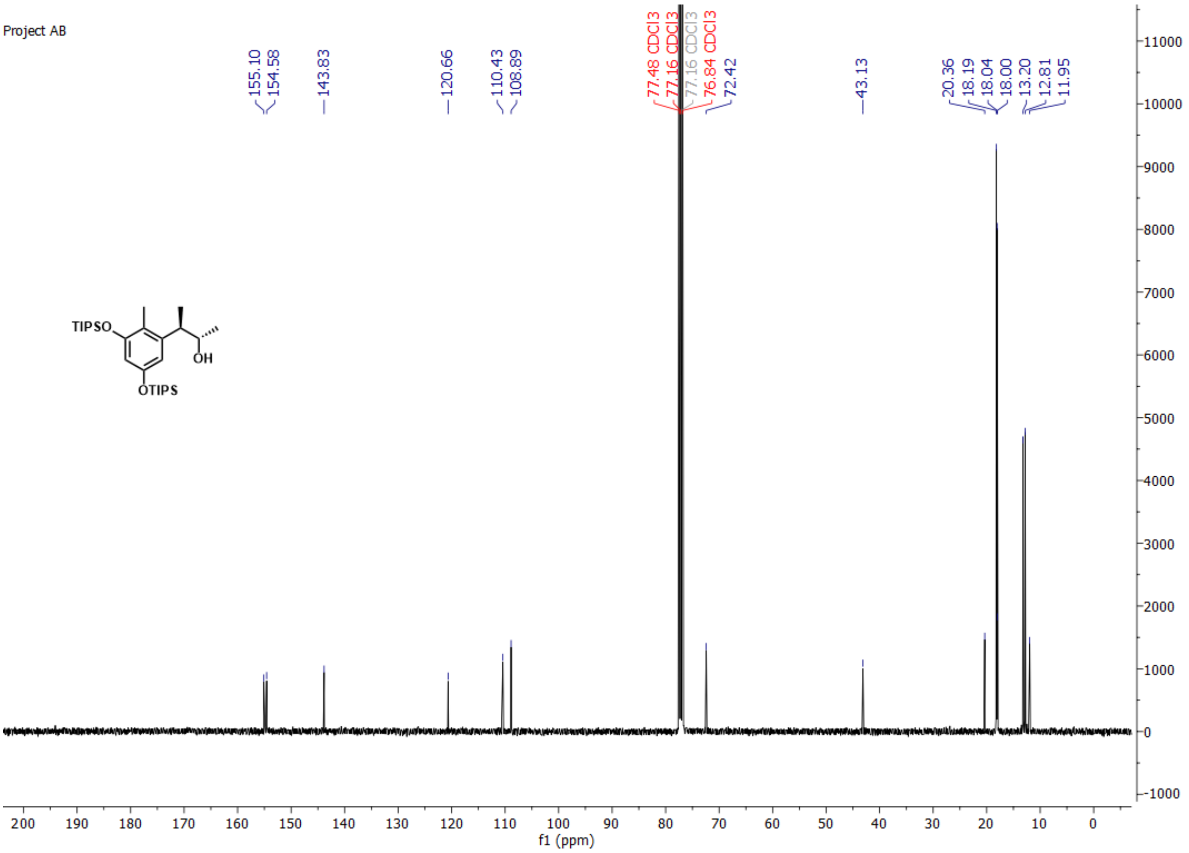

^{1}H (400 MHz) and $^{13}C\{^{1}H\}$ (101 MHz) NMR spectra of compound **(5)** ((+)-phenol A) in Methanol-*d4*.

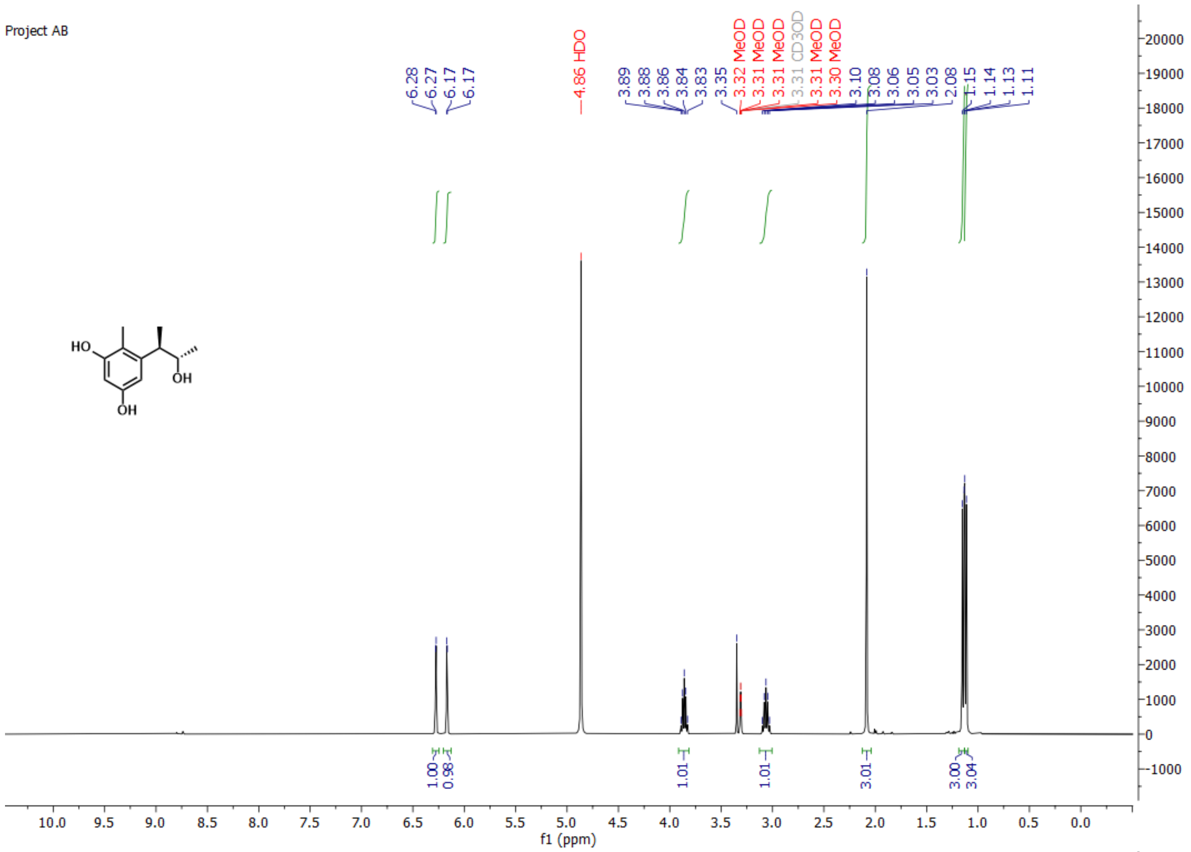

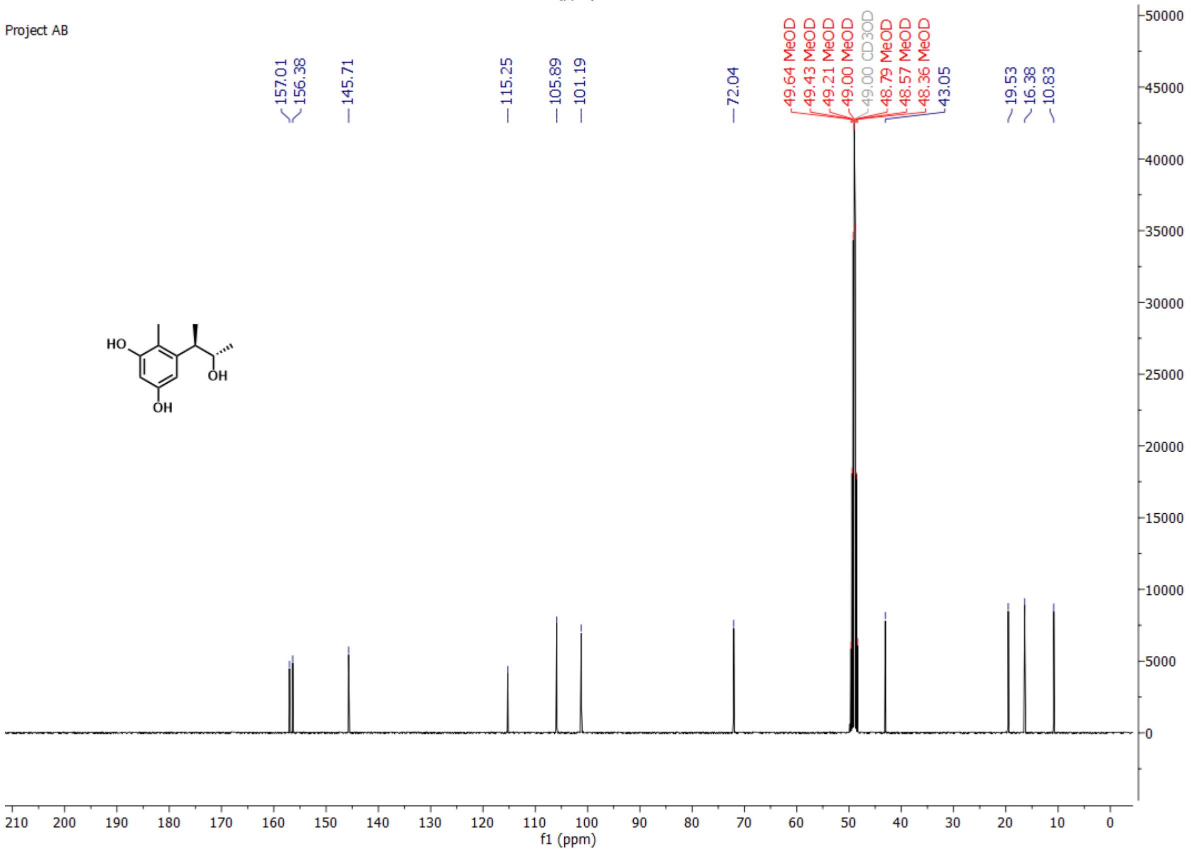

^{1}H (400 MHz) and $^{13}C\{^{1}H\}$ (101 MHz) NMR spectra of compound **27** in Chloroform-*d*.

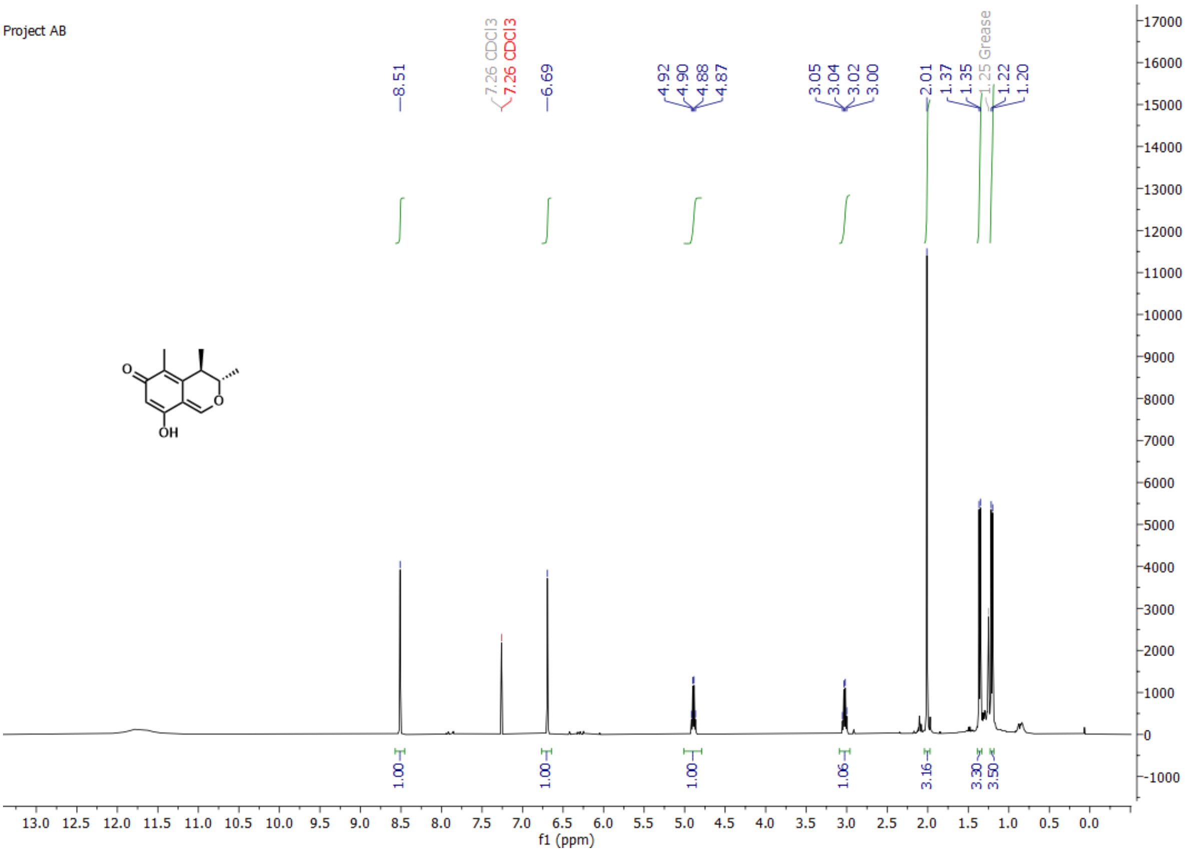

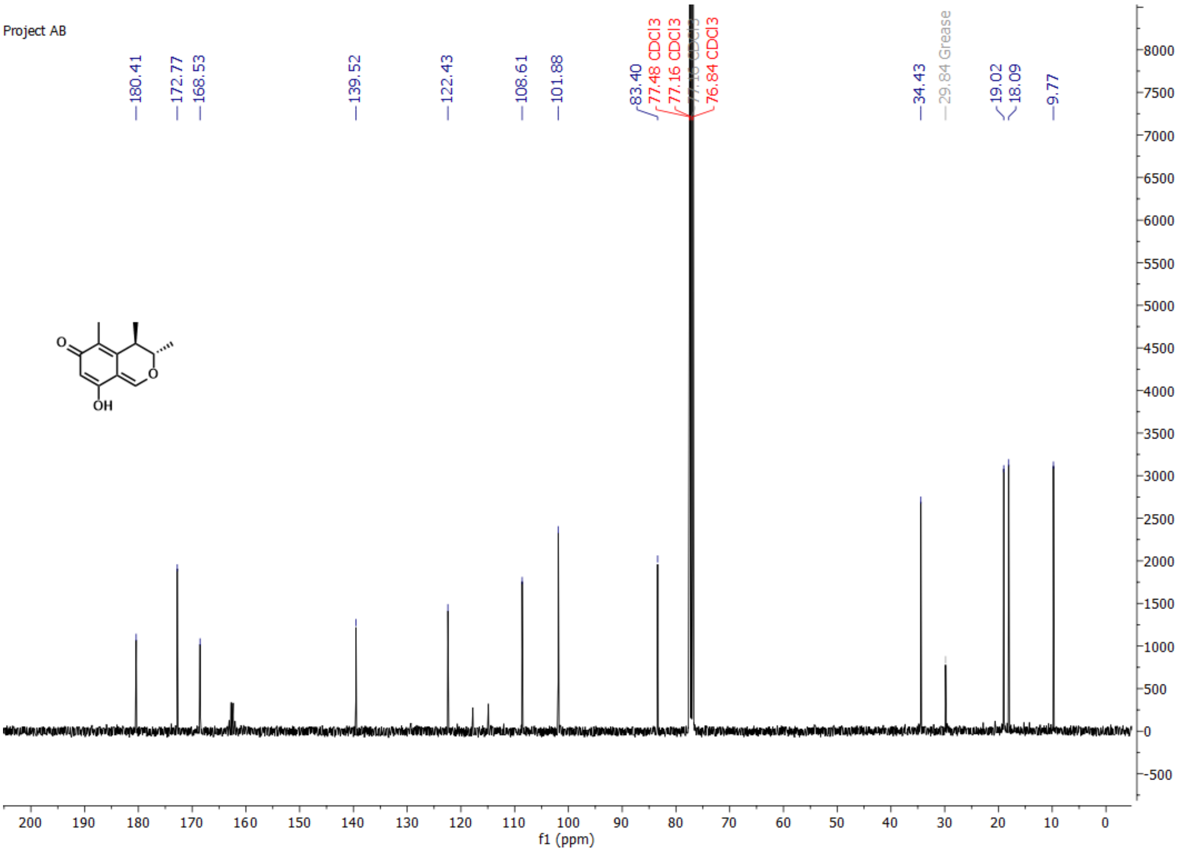

^{1}H (400 MHz) and ^{13}C{^{1}H} (101 MHz) NMR spectra of compound **2** ((–)-aspergilone A) in Chloroform-*d*.

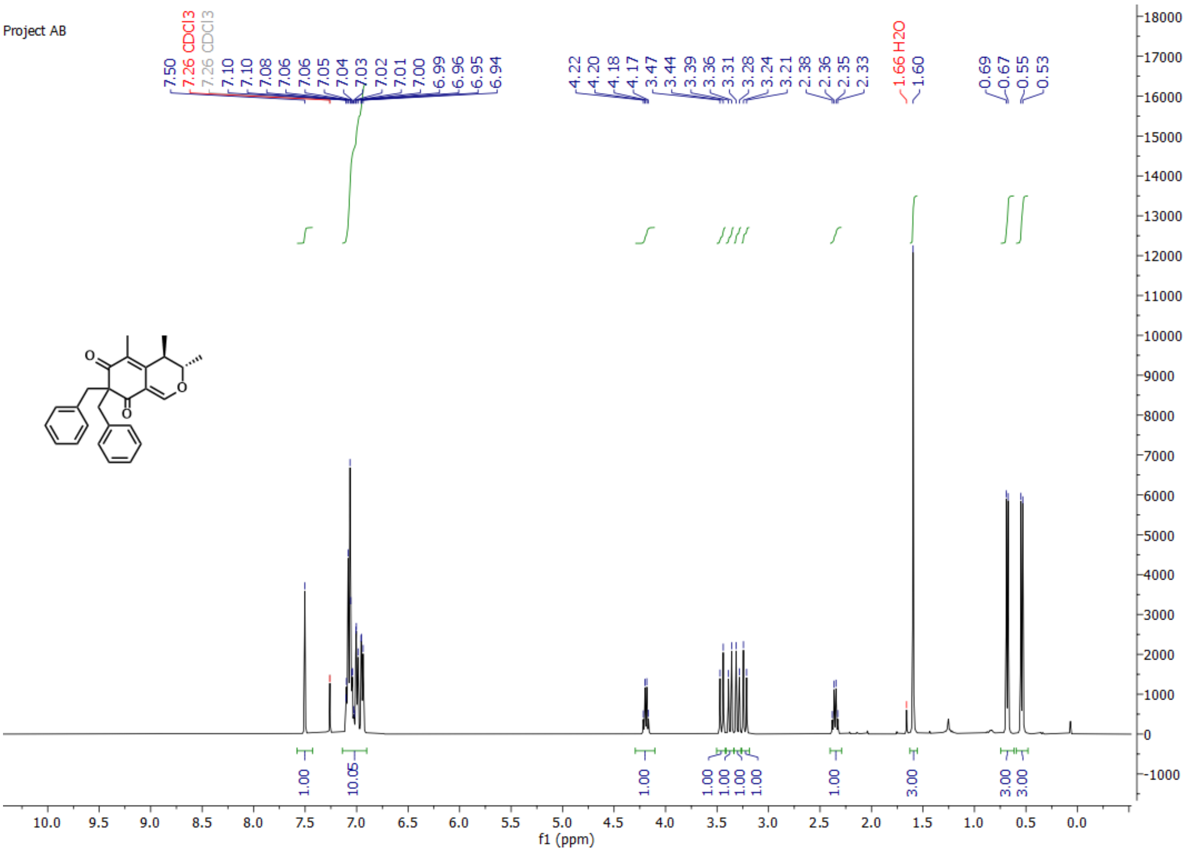

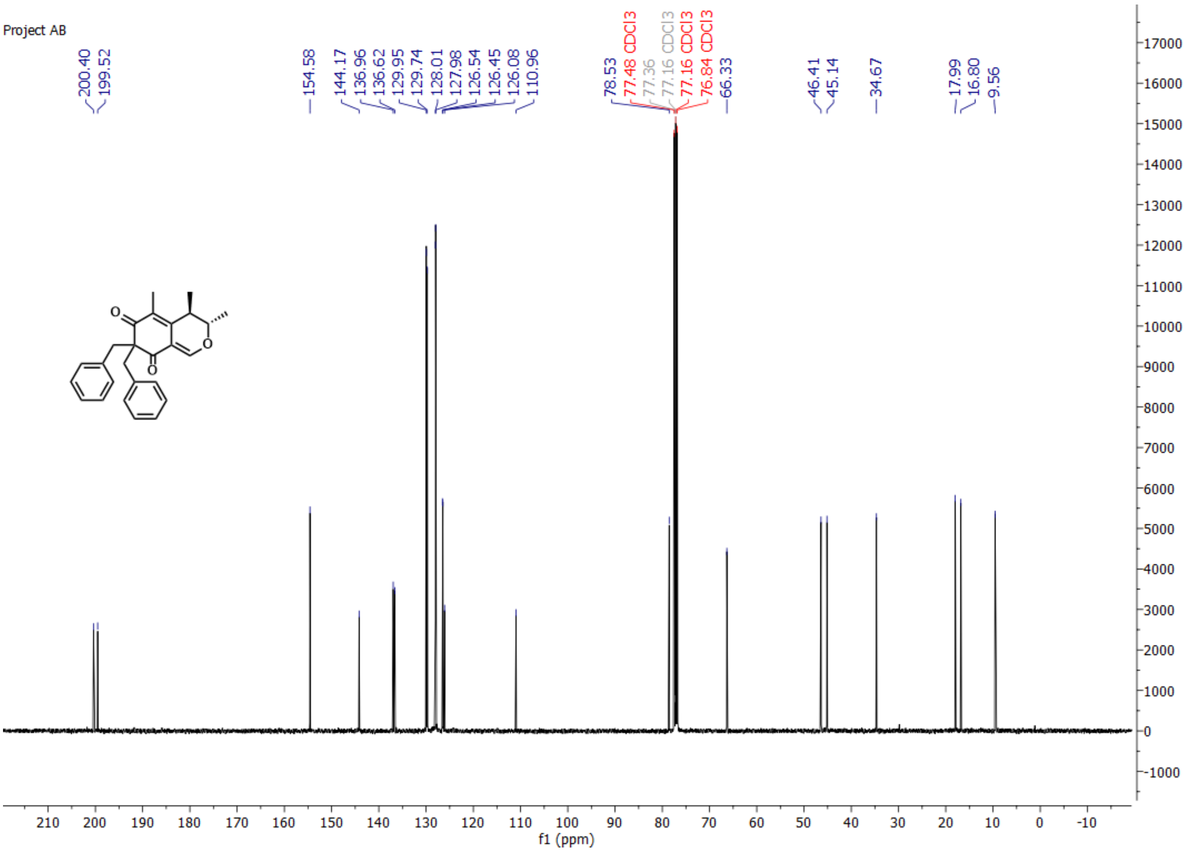

^{1}H (400 MHz) and $^{13}C\{^{1}H\}$ (101 MHz) NMR spectra of compound rac-*erythro*-**26** in Chloroform-*d*.

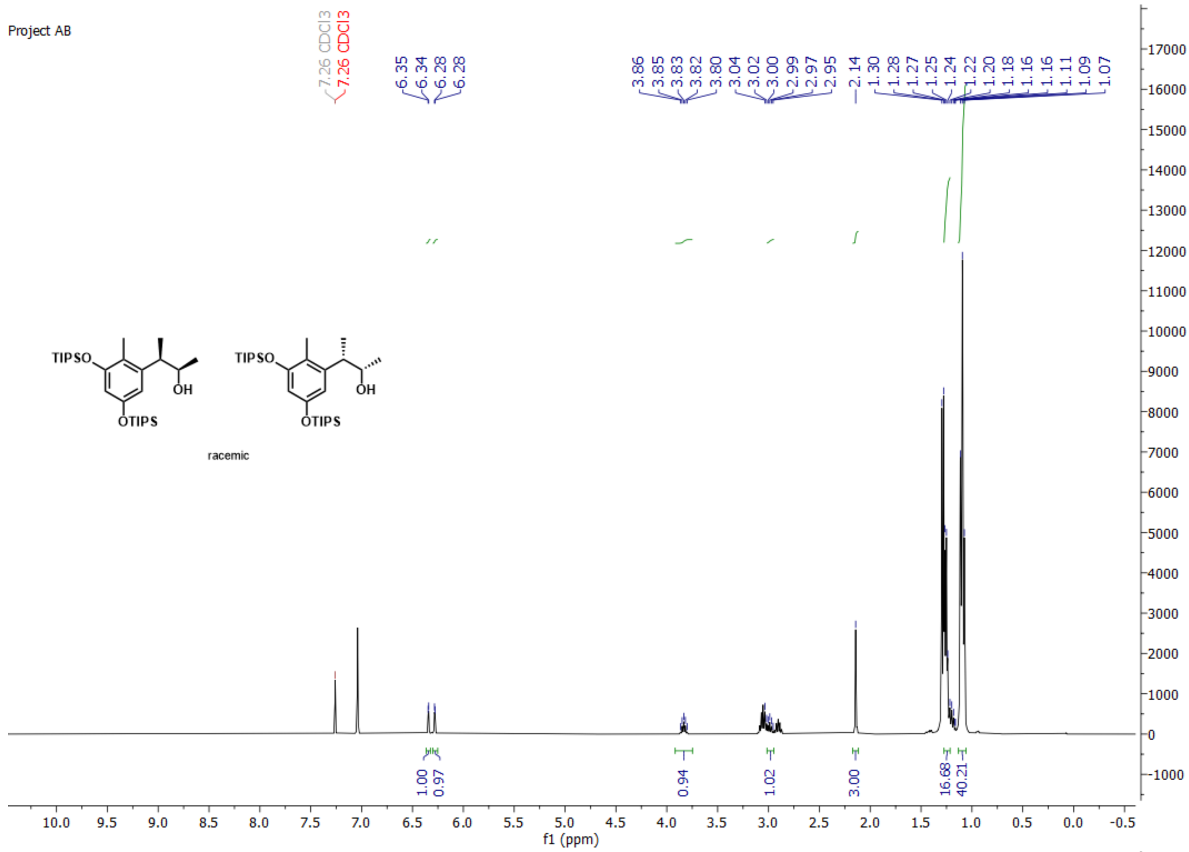

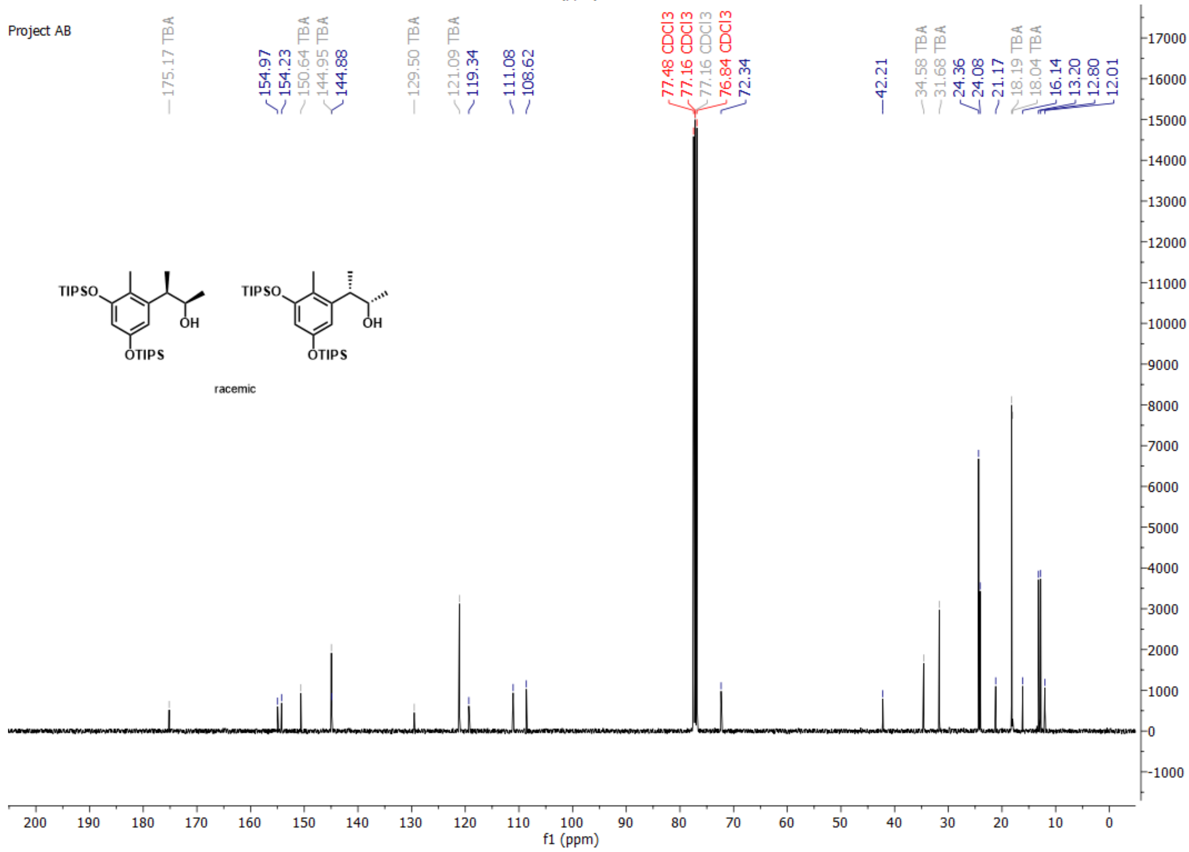

^{1}H (400 MHz) and ^{13}C{^{1}H} (101 MHz) NMR spectra of compound **S11** in Chloroform-*d*.

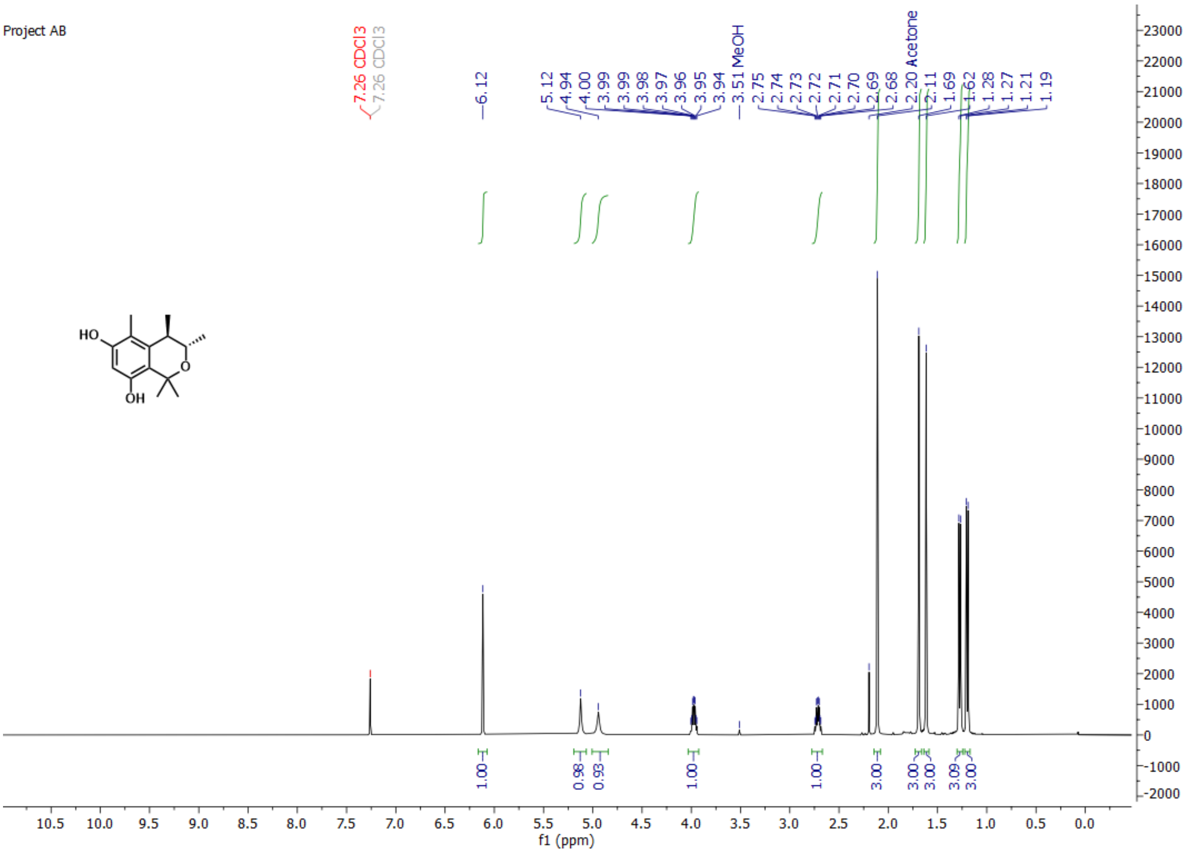

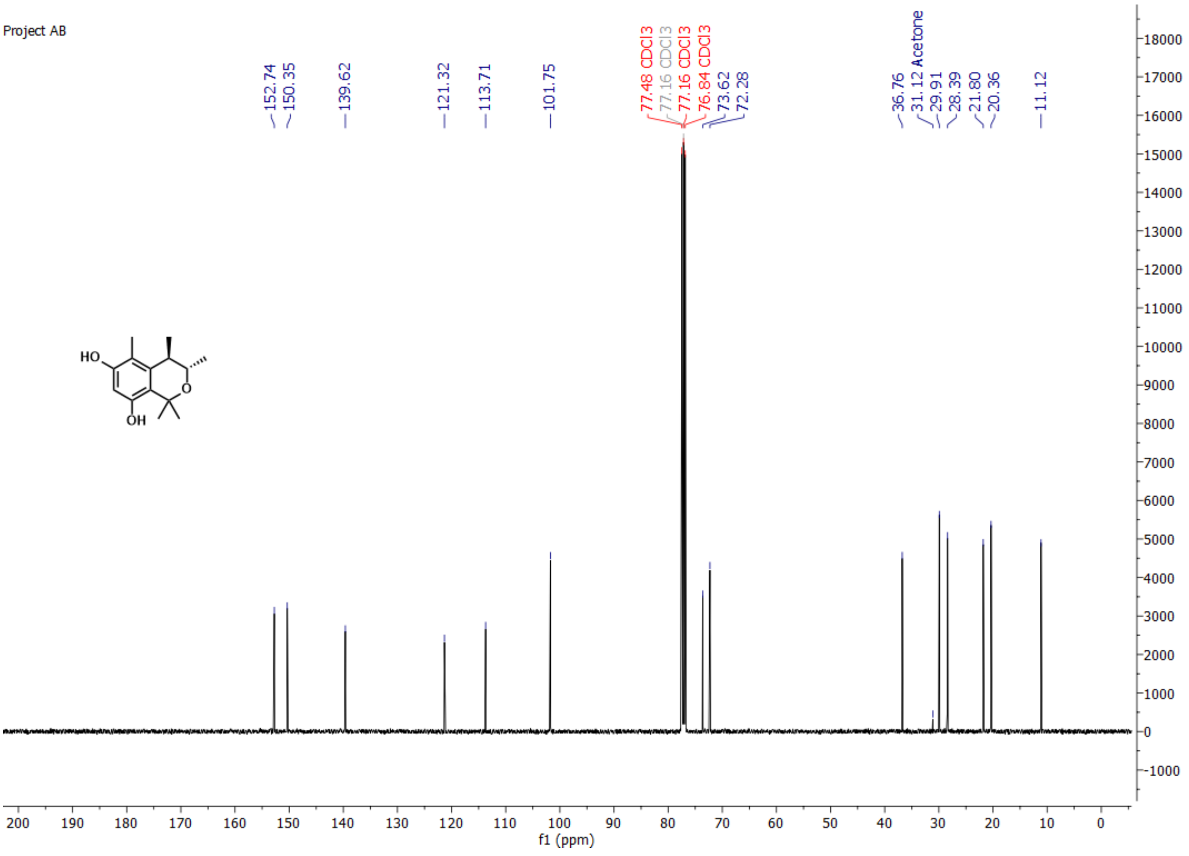

^{1}H (400 MHz) and ^{13}C{^{1}H} (101 MHz) NMR spectra of compound **S12** in Chloroform-*d*.

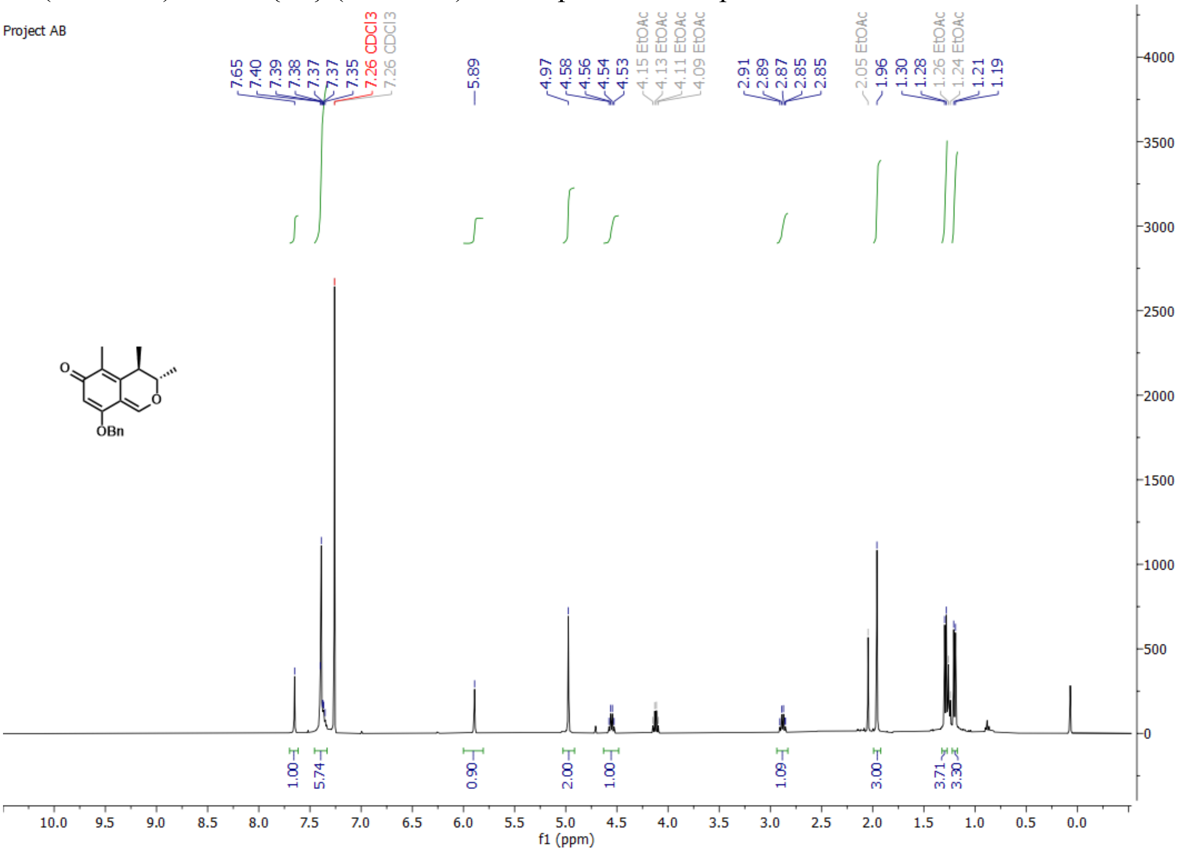

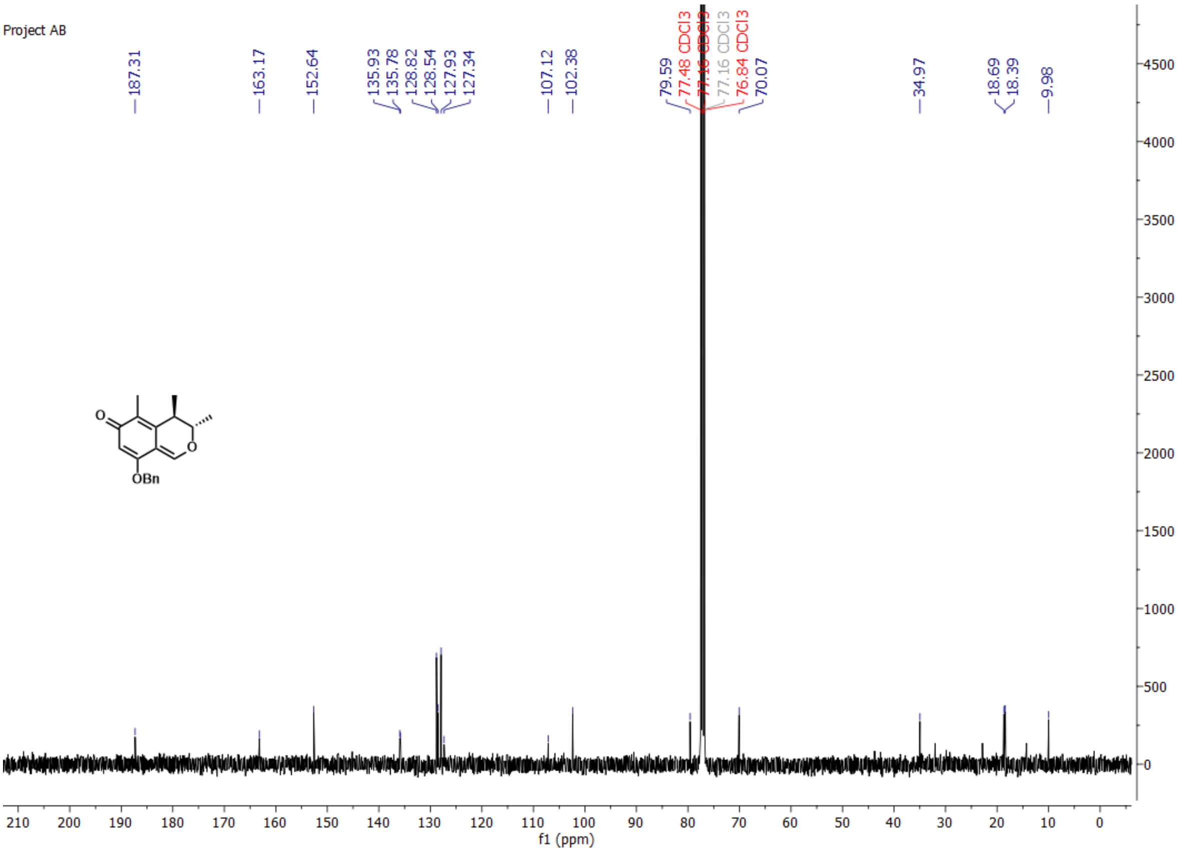

5. IR Spectra

2-bromo-4,6-dimethoxybenzaldehyde **17**.

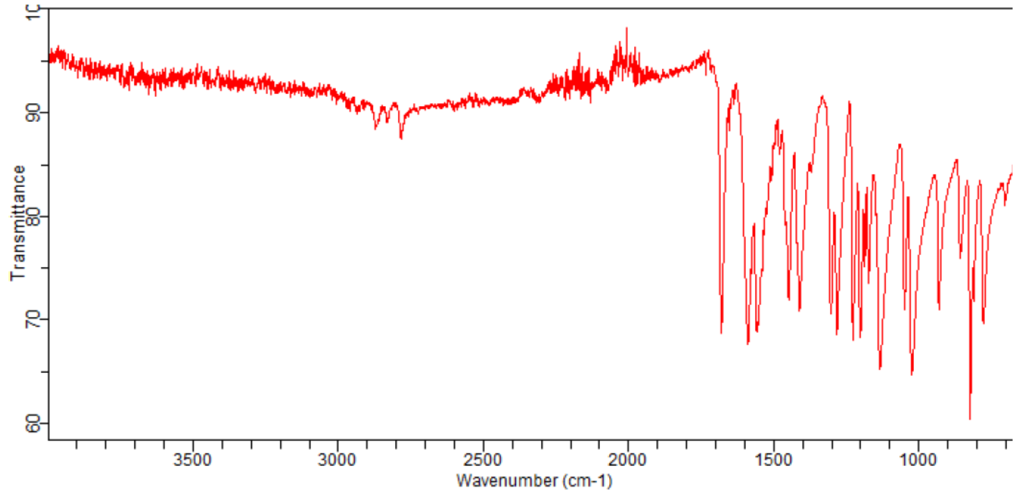

1-bromo-3,5-dimethoxy-2-methylbenzene **18**.

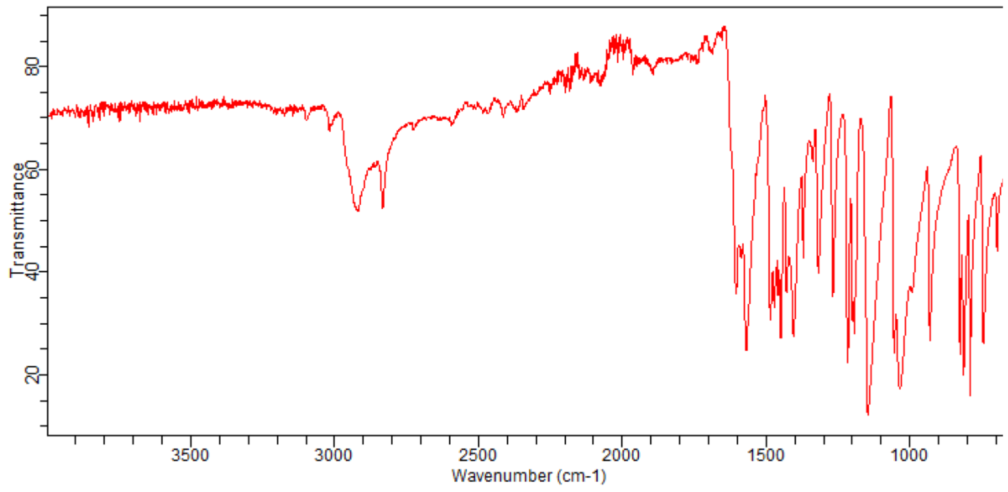

5-bromo-4-methylbenzene-1,3-diol **19**.

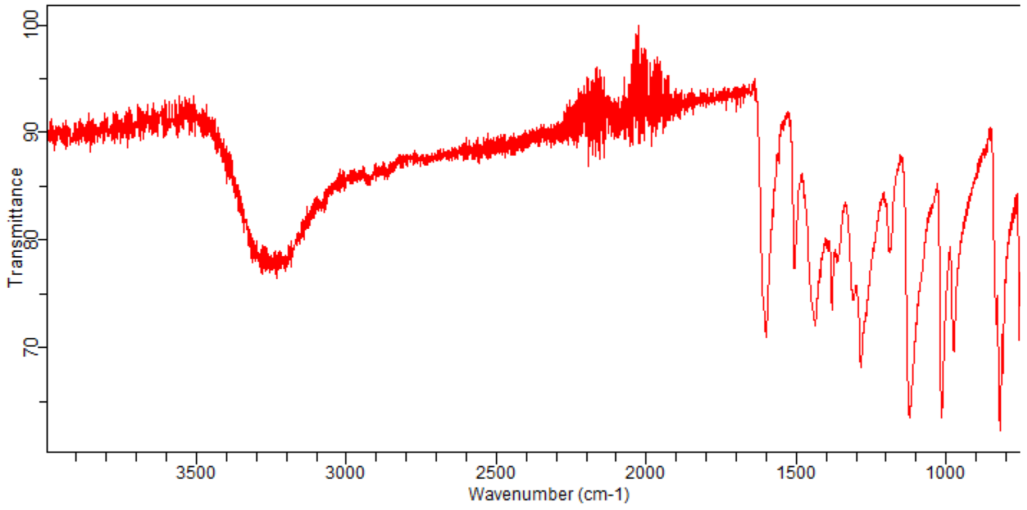

((5-bromo-4-methyl-1,3-phenylene)bis(oxy))bis(triisopropylsilane) **20**.

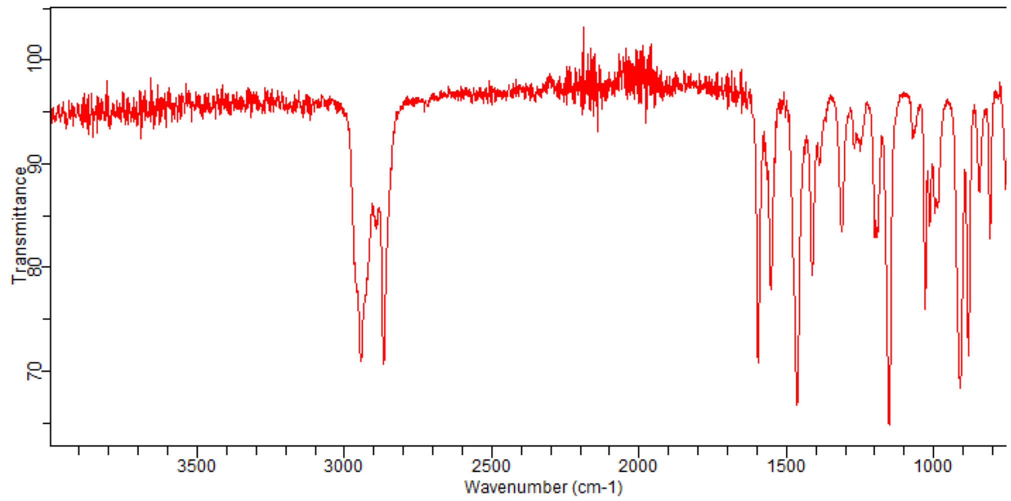

((4-methyl-5-(4,4,5,5-tetramethyl-1,3,2-dioxaborolan-2-yl)-1,3-phenylene)bis(oxy))bis(triisopropylsilane) **12**.

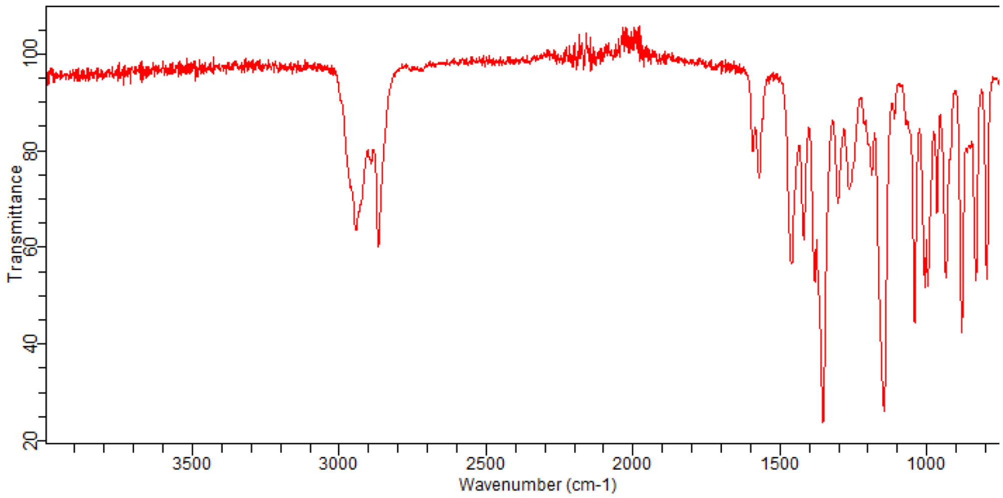

(R)-((4-methyl-5-(1-(4,4,5,5-tetramethyl-1,3,2-dioxaborolan-2-yl)ethyl)-1,3-phenylene)bis(oxy))bis(triisopropylsilane) **23**.

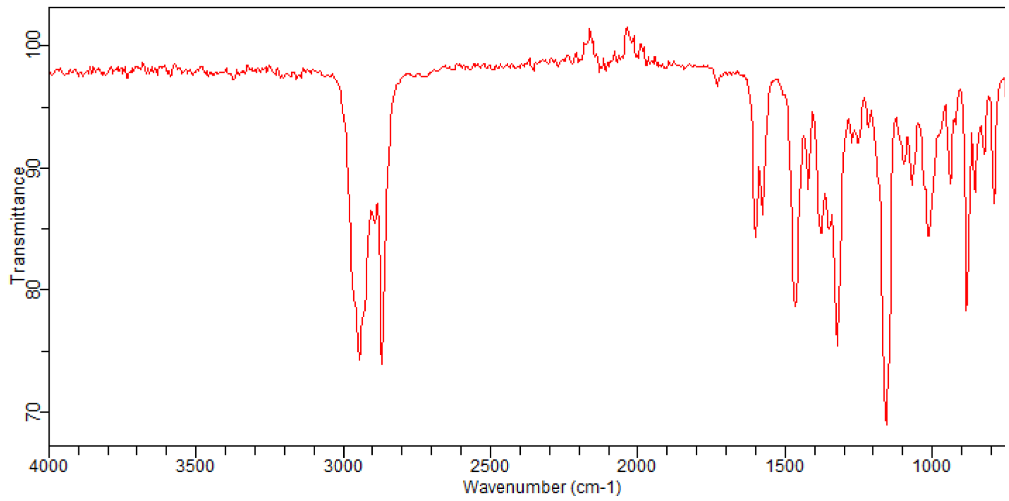

(+)-(R)-1-(2-methyl-3,5-bis((triisopropylsilyl)oxy)phenyl)ethan-1-ol **S10**.

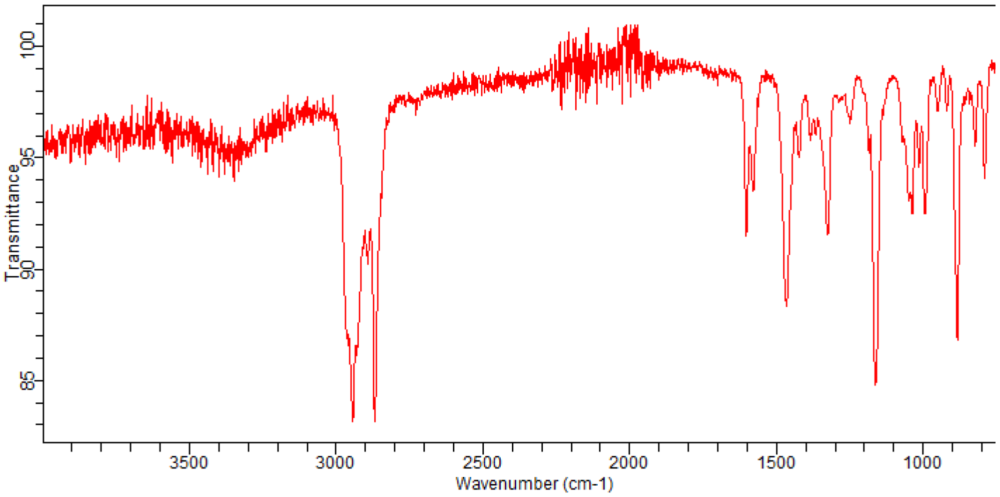

(+)-(2S,3R)-3-(2-methyl-3,5-bis((triisopropylsilyl)oxy)phenyl)butan-2-ol **26**.

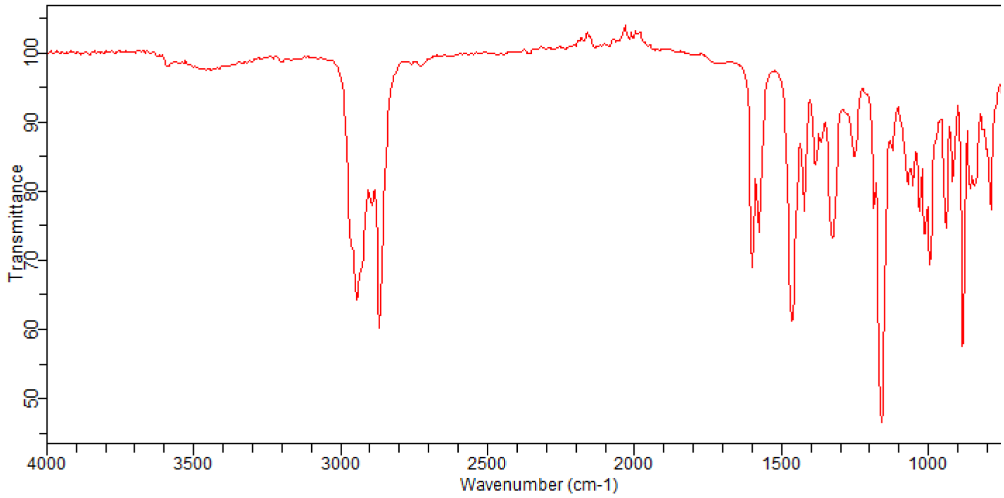

(+)-5-((2R,3S)-3-hydroxybutan-2-yl)-4-methylbenzene-1,3-diol **5** ((+)-phenol A).

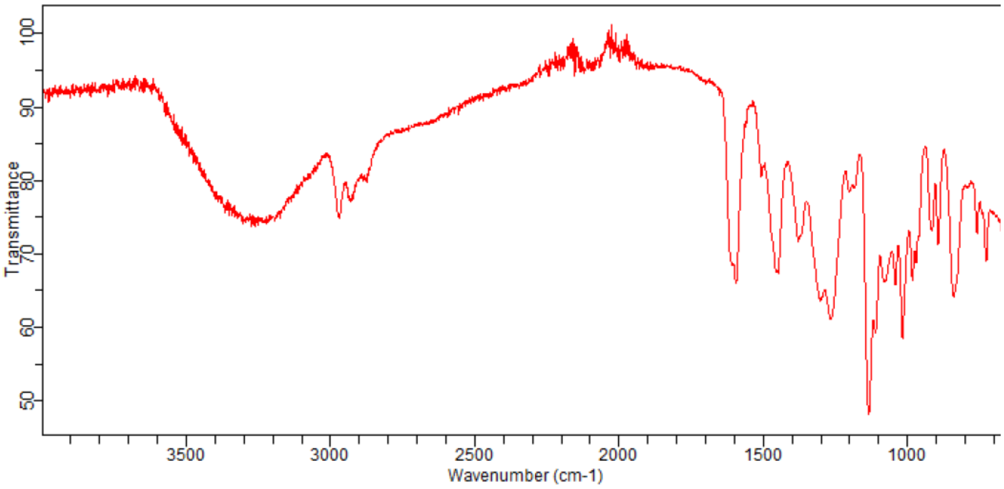

(+)-(3S,4R)-8-hydroxy-3,4,5-trimethyl-3,4-dihydro-6H-isochromen-6-one **27**.

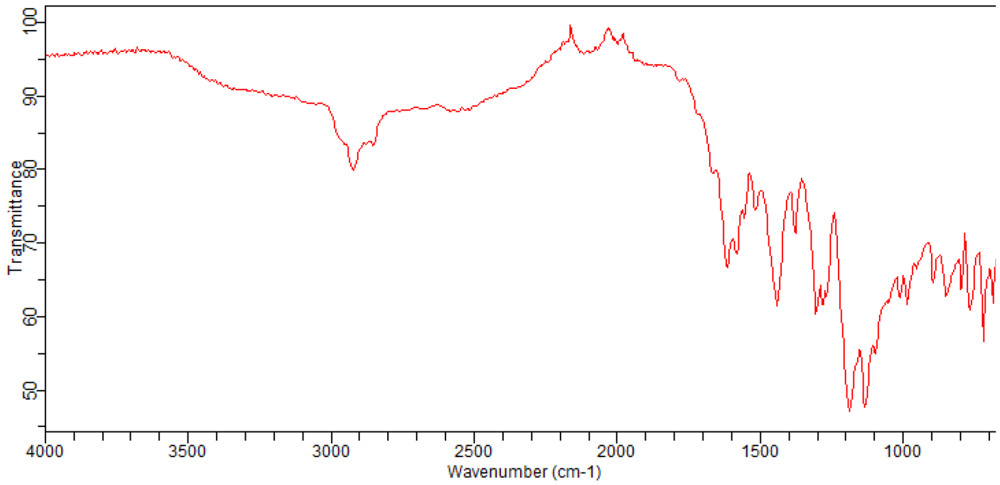

(–)-(3S,4R)-7,7-dibenzyl-3,4,5-trimethyl-3,4-dihydro-6H-isochromene-6,8(7H)-dione **2** ((–)-aspergilone A).

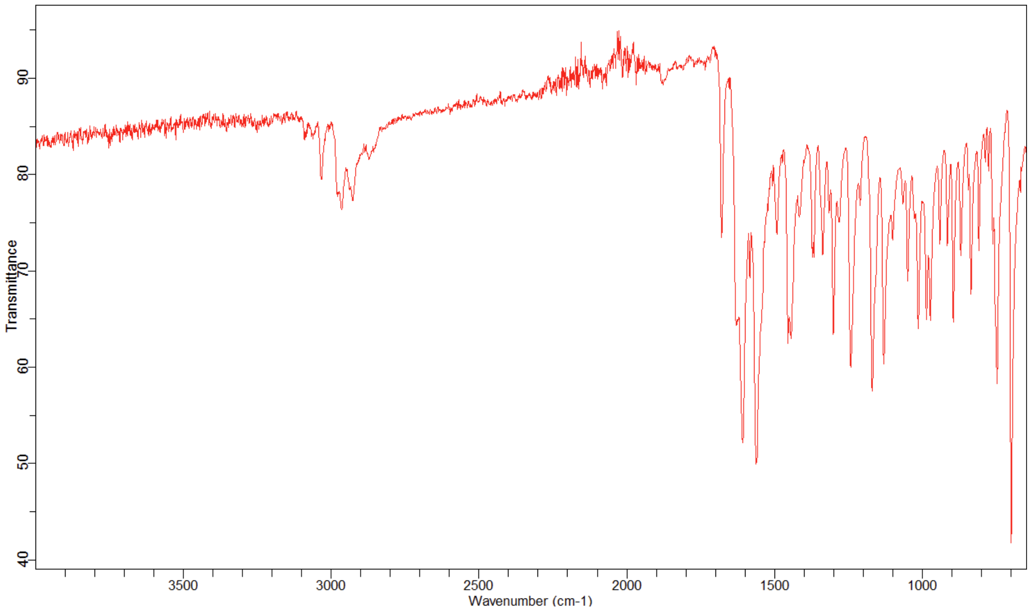

(3S,4R)-1,1,3,4,5-pentamethylisochromane-6,8-diol ***S11***.

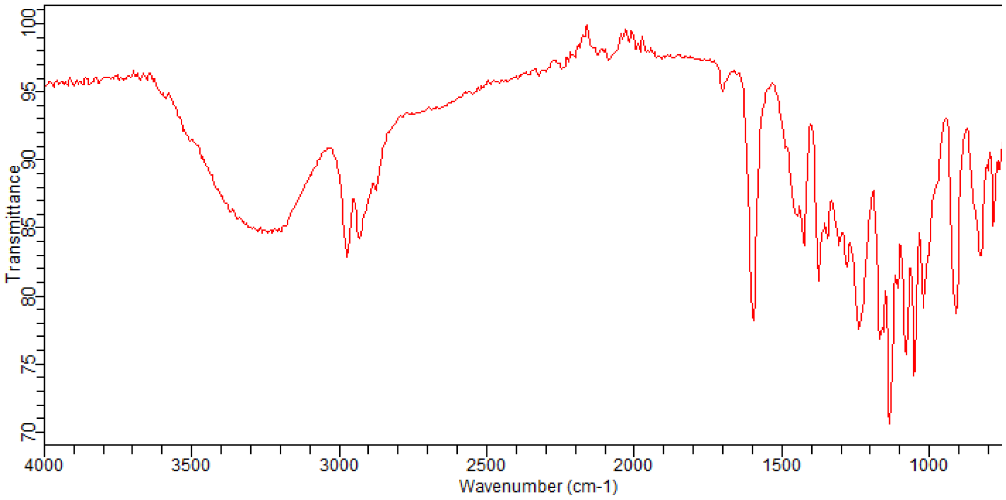

6. References

(1) Shao, C.-L.; Wang, C.-Y.; Wei, M.-Y.; Gu, Y.-C.; She, Z.-G.; Qian, P.-Y.; Lin, Y.-C. Aspergilones A and B, two benzylazaphilones with an unprecedented carbon skeleton from the gorgonian-derived fungus Aspergillus sp. *Bioorg. Med. Chem. Lett.* **2011**, *21* (2), 690-693. DOI: 10.1016/j.bmcl.2010.12.005.
(2) Vickery, E. H.; Pahler, L. F.; Eisenbraun, E. J. Selective O-demethylation of catechol ethers. Comparison of boron tribromide and iodotrimethylsilane. *J. Org. Chem.* **1979**, *44* (24), 4444-4446. DOI: 10.1021/jo01338a043.
(3) Ghiaci, M.; Asghari, J. Dealkylation of Alkyl and Aryl Ethers with $AlCl_3$ - NaI in the Absence of Solvent. *Synth. Commun.* **1999**, *29* (6), 973-979. DOI: 10.1080/00397919908086060.
(4) Hetherington, A. C. R., H. Biochemistry of microorganisms. XIV. Production and chemical constitution of a new yellow coloring matter, citrinin, produced from dextrose by *Penicillium citrinum*. *Trans. Roy. Soc. (London)* **1931**, *B220*, 269-296.
(5) Barber, J. A.; Staunton, J.; Wilkinson, M. R. A diastereoselective synthesis of the polyketide antibiotic citrinin using toluate anion chemistry. *J. Chem. Soc., Perkin Trans. 1* **1986**, 2101-2109. DOI: 10.1039/P19860002101.
(6) Akiyama, T.; Hirofuji, H.; Ozaki, S. $AlCl_3$-N,N-dimethylaniline: A new benzyl and allyl ether cleavage reagent. *Tetrahedron Lett.* **1991**, *32* (10), 1321-1324. DOI: 10.1016/S0040-4039(00)79656-0.
(7) Majeed, M. T., Samuel Manoharan; Nagabhushanam, Kalyanam; Balakrishnan, Sivaprakash Kurumanghat; Prakash, Subbalakshmi. A Process for the Synthesis of Biologically Active Oxygenated Compounds by Dealkylation of the Corresponding Alkylethers. US 2008.
(8) Majetich, G.; Zhang, Y.; Wheless, K. Hydride-promoted demethylation of methyl phenyl ethers. *Tetrahedron Lett.* **1994**, *35* (47), 8727-8730. DOI: 10.1016/S0040-4039(00)78482-6.
(9) Magano, J.; Chen, M. H.; Clark, J. D.; Nussbaumer, T. 2-(Diethylamino)ethanethiol, a New Reagent for the Odorless Deprotection of Aromatic Methyl Ethers. *J. Org. Chem.* **2006**, *71* (18), 7103-7105. DOI: 10.1021/jo0611059.
(10) Hwu, J. R.; Tsay, S. C. Counterattack reagents sodium trimethylsilanethiolate and hexamethyldisilathiane in the bis-O-demethylation of aryl methyl ethers. *J. Org. Chem.* **1990**, *55* (24), 5987-5991. DOI: 10.1021/jo00311a016.
(11) Pangborn, A. B.; Giardello, M. A.; Grubbs, R. H.; Rosen, R. K.; Timmers, F. J. Safe and Convenient Procedure for Solvent Purification. *Organometallics* **1996**, *15* (5), 1518-1520. DOI: 10.1021/om9503712.
(12) Bagutski, V.; French, R. M.; Aggarwal, V. K. Full Chirality Transfer in the Conversion of Secondary Alcohols into Tertiary Boronic Esters and Alcohols Using Lithiation–Borylation Reactions. *Angew. Chem. Int. Ed.* **2010**, *49* (30), 5142-5145. DOI: 10.1002/anie.201001371.
(13) Burns, M.; Essafi, S.; Bame, J. R.; Bull, S. P.; Webster, M. P.; Balieu, S.; Dale, J. W.; Butts, C. P.; Harvey, J. N.; Aggarwal, V. K. Assembly-line synthesis of organic molecules with tailored shapes. *Nature* **2014**, *513* (7517), 183-188. DOI: 10.1038/nature13711.
(14) Sommers, A. H.; Michaels, R. J.; Weston, A. W. Formylation of the Dimethoxygenzenes Using Phosphorus Oxychloride and N-Methylformanilide. *J. Am. Chem. Soc.* **1952**, *74* (21), 5546-5546. DOI: 10.1021/ja01141a533.
(15) Gadhwal, S.; Baruah, M.; Sandhu, J. S. Microwave Induced Synthesis of Hydrazones and Wolff-Kishner Reduction of Carbonyl Compounds. *Synlett* **1999**, *1999* (10), 1573-1574. DOI: 10.1055/s-1999-2901.
(16) Rödel, T.; Gerlach, H. Enantioselective synthesis of the polyketide antibiotic (3*R*,4*S*)-(−)-citrinin. *Liebigs Ann. Chem.* **1995**, *1995* (5), 885-888. DOI: 10.1002/jlac.1995199505129.
(17) Johnson, D. H.; Robertson, A.; Whalley, W. B. 580. The chemistry of fungi. Part XIII. Citrinin. *J. Chem. Soc. (Resumed)* **1950**, 2971-2975. DOI: 10.1039/JR9500002971.
